# Suppressing gain-of-function proteins via CRISPR/Cas9 system in SCA1 cells

**DOI:** 10.1038/s41598-022-24299-y

**Published:** 2022-11-24

**Authors:** Mariangela Pappadà, Ottavia Bonuccelli, Mattia Buratto, Riccardo Fontana, Mariaconcetta Sicurella, Anna Caproni, Silvia Fuselli, Andrea Benazzo, Roberto Bertorelli, Veronica De Sanctis, Paolo Cavallerio, Valentina Simioni, Valeria Tugnoli, Francesca Salvatori, Peggy Marconi

**Affiliations:** 1 Department of Chemical, Pharmaceutical and Agricultural Sciences, University of Ferrara, via Fossato di Mortara 64/B, 44121 Ferrara, Italy; 2 Department of Life Sciences and Biotechnology, University of Ferrara, via L. Borsari 46, 44121 Ferrara, Italy; 3 Department of Environmental Sciences and Prevention, University of Ferrara, via L. Borsari 46, 44121 Ferrara, Italy; 4 Next Generation Sequencing Core Facility, Department of Cellular, Computational and Integrative Biology-CIBIO, University of Trento, via Sommarive 9, 38123 Povo, Trento Italy; 5 Division of Neurology, Department of Neuroscience and Rehabilitation, University Hospital of Ferrara, via A. Moro 8, 44100 Ferrara, Cona Italy

**Keywords:** CRISPR-Cas9 genome editing, Genetic techniques, Genetic engineering

## Abstract

SCAs are autosomal dominant neurodegenerative disorders caused by a gain-of-function protein with toxic activities, containing an expanded polyQ tract in the coding region. There are no treatments available to delay the onset, stop or slow down the progression of these pathologies. In this work we focus our attention on SCA1 which is one of the most common genotypes circulating in Italy. Here, we develop a CRISPR/Cas9-based approach to reduce both forms of the ATXN1 protein, normal and mutated with expanded polyQ. We started with the screening of 10 different sgRNAs able to target Exon 8 of the *ATXN1* gene. The two most promising sgRNAs were validated in fibroblasts isolated from SCA1 patients, following the identification of the best transfection method for this type of cell. Our silencing approach significantly downregulated the expression of ataxin1, due to large deletions and the introduction of small changes in the *ATXN1* gene, evidenced by NGS analysis, without major effects on cell viability. Furthermore, very few significant guide RNA-dependent off-target effects were observed. These preliminary results not only allowed us to identify the best transfection method for SCA1 fibroblasts, but strongly support CRISPR/Cas9 as a promising approach for the treatment of expanded polyQ diseases. Further investigations will be needed to verify the efficacy of our silencing system in SCA1 neurons and animal models.

## Introduction

PolyQ spinocerebellar ataxias (SCA1, SCA2, SCA3, SCA6, SCA7 and SCA17) are a group of autosomal dominant neurodegenerative diseases, caused by the expansion of the CAG triplet repeats present in the coding region of specific genes, which are translated into an expanded polyglutamine tract in their encoded proteins^1^. The size of the triplet expansion varies from individual to individual and is directly related to the severity and age of onset of symptoms. Furthermore, given the strong instability of the repeated trait, SCAs are characterized by the phenomenon of genetic anticipation, which provides for an increase in severity and an advance of the onset of symptoms through the generations^2^.

Proteins with expanded polyQ assume different conformations following an aberrant folding, which increases their stability and induces the acquisition of new toxic functions, such as the aberrant interaction with transcription and splicing factors. They also tend to accumulate in aggregates both in the cytosol and in the nucleus of target cells. The pathogenetic significance of these inclusions has not yet been fully clarified, but a valid hypothesis foresees a double role: protective in the early stages of the disease and pathogenetic in the late stages. A mechanism of neuronal dysfunction caused by nuclear aggregates could be the sequestration of proteins essential for nuclear functions, such as transcription factors, while both nuclear and cytoplasmic inclusions could cause the physical destruction of normal cellular functions, damaging the mitochondria, chaperone and ubiquitin proteosome system^3,4^.

PolyQ SCAs, which have a frequency of 2–3 cases per 100,000 people, are progressive, typically striking in midlife and causing increasing neuronal dysfunction and eventual neuronal loss 10–20 years after onset of symptoms^5^. Patients can lose the ability to breathe in a coordinated fashion, which can be fatal^6^.

The diseases have a bimodal presentation: individuals with typical onset during the fourth decade manifest a pure cerebellar phenotype while individuals with juvenile onset show more rapid progression and more severe disease. Early in the disease, the prominent symptoms are gait and balance disturbance, slurred speech, brisk deep tendon reflexes, hypermetric saccades, nystagmus, and mild dysphagia. Later signs include development of up-gaze palsy, muscle atrophy, frontotemporal dementia (FTD), chorea, dystonia, and bulbar dysfunction. An axonal sensory neuropathy is common; brain imaging typically shows cerebellar and brain stem atrophy. Interval from onset to death varies from 10 to 30 years^7^. In polyQ SCAs, only a certain subset of neurons is vulnerable to dysfunction, despite the widespread expression of the relevant protein throughout the brain and other tissues^8^.

Currently, no treatments are available to prevent or cure, delay the onset, stop, or slow down the progression of these diseases. Several research groups are trying to develop strategies to reduce the levels of mutated proteins, and therefore their neurotoxic effects, using for example antiaggregant agents^5^, molecules capable of activating the ubiquitin–proteasome pathway^9^ and compounds with autophagic effects, as lithium^10^, tensirolimus^11^, thehalose^12^ and Beclin-1^13^.

Potential genetic therapies for polyQ SCAs involve several different nucleic acid-based molecules able to target the RNA of the polyQ-associated genes, resulting in the suppression of the encoded toxic protein. Since methods exploiting the mechanisms involved in the processing of endogenous miRNAs, as siRNAs and shRNAs, have shown potential toxicity^14^, antisense oligonucleotide-mediated (ASO-mediated) RNA suppression approaches have been recently used to reduce gene expression and improve disease symptoms in preclinical rodent models of several neurological diseases^15–17^. The disadvantages of these methods lie in the need for continuous administration throughout the patient's life, to keep toxic protein levels low, and in their great difficulty in passing the blood–brain barrier, which is why in animal models the administration is intra-ventricular^18^.

To address these fundamental limitations, the field of gene editing has emerged to make precise, targeted modifications to genome sequences. Currently, the most popular and used tool for gene editing is the clustered regularly interspaced short palindromic repeat (CRISPR)/Cas9 system^19^, a component of the bacterial RNA-mediated adaptive immune system. It consists of transcribed guide RNAs, easily programmable by the operator, that direct the RNA-guided Cas9 DNA endonuclease to target sequences^19,20^. The site-specific double strand cut determined by the CRISPR/Cas9 system activates the repair mechanisms of DNA double strand break, which can be exploited to correct, introduce, or silence a gene. The CRISPR/Cas9 system from *Streptococcus pyogenes* has already been successfully used in treating genetic disorders^19,21^.

So far, within the polyQ SCAs, two different research groups adopted the CRISPR/Cas9 system to develop a gene silencing strategy in one case^22^ and a polyQ expansion correction approach in the other^23^ as part of their studies on SCA3. A similar procedure to the latter, but using zinc finger nucleases, has also been developed for FRDA^24^. Although these strategies have been validated on a small number of cell samples, they demonstrated the feasibility of using gene editing techniques for the silencing or the correction of a gene with triplet expansion.

Our work aims on the one hand to confirm the possibility of using the CRISPR/Cas9 system for the suppression of proteins with expanded polyQ, this time in the case of SCA1, on the other hand to verify whether the cellular variability, typical of each individual, and the distance between the two cutting sites can affect the efficiency of the gene editing system. To test our gene suppression system on a high number of different patient cells and have a proof-of-concept evaluation of its effectiveness, we decided to use dermal fibroblasts as the cellular model of choice, strengthened by the fact that they had already been used to verify the precise excision of the CAG tract expanded from the huntingtin gene using Cas9 nickases^25^.

## Materials and methods

All the methods were performed in accordance with relevant guidelines and regulations.

### Oligonucleotides

Oligonucleotides used in sgRNA screening, PCR reactions and NGS libraries production were designed using Primer-BLAST and purchased from IDT (INTEGRATED DNA TECHNOLOGIES, Coralville, Iowa, USA). All sequences are reported in Tables S1, S2 and S3, in supplementary Material section.

Each primer used for the NGS libraries production was designed in triplicate to increase the samples complexity; the three variants of these primers were mixed to create isomolar solutions (10 µM) to set up PCR reactions.

### sgRNA design and screening

sgRNAs were designed by CRISPR Design software, developed by Zhang at the MIT Laboratory in 2015^26^, considering exon 8 of the ATXN1 gene. Ten different sgRNAs targeted to sequences upstream and downstream the *ATXN1* polyQ tract were identified, named G3, G4, G5, G6, G7, G8, G9, G10 G11 and G12. To produce and test the efficacy of designed sgRNA, Guide-it In Vitro Transcription and Screening Kit (TAKARA BIO USA, Mountain View, California) was used, according to manufacturer’s protocol. This kit contains the components needed to produce the cutting template, in vitro transcribe the sgRNAs, purify them and test their efficacy against the PCR target fragment using recombinant Cas9 nuclease. The forward primers used for the generation of the sgRNAs transcription templates, and the primers utilized in the PCR reaction to produce the cutting template were designed using the instruction recommended in Guide-it In Vitro Transcription and Screening kit user manual and are reported in Tables S1 and S2, respectively. pDEST27-GST-ATXN1 FL(30Q), a plasmid containing the *ATXN1* coding sequence, was used as template for the PCR reactions, since the two primers (TempForw and TempRev) annealed into different exons. The selected sgRNAs were then purchased from the SYNTHEGO (Menlo Park, California) company.

pDEST27-GST-ATXN1 FL(30Q) was a gift from Huda Zoghbi (Addgene plasmid #21753; http://n2t.net/addgene:21753; RRID:Addgene_21753).

The G3N and G8N sgRNAs were designed using CRISPR design tool from SYNTHEGO (Menlo Park, California).

### Sample-size estimation

The calculation of the sample size was made, assuming a 30% variation in efficacy in the treatment compared to the negative control, a 1st type error α equal to 0.05 and a power β of 0.80. The sample size calculation was equal to 5 cell samples.

### Cell cultures

Cultures of normal human fibroblasts (AG21802, AG21754, AG21805) were provided by the Coriell Biorepository (CORIELL INSTITUTE FOR MEDICAL RESEARCH, New Jersey USA). These cell lines were maintained in DMEM High Glucose Medium (EUROCLONE, Pero, Italy) supplemented with 20% not heat-inactivated fetal bovine serum (FBS) (EUROCLONE, Pero, Italy), 1× of Antibiotic Antimycotic Solution 100× (SIGMA, Saint Louis, Missouri). Since these cell cultures grow in adhesion, before dividing them, they were detached from the flask using trypsin–EDTA 1× (EUROCLONE S.p.A., Pero, Italy). The cultures were incubated at 37 °C in humidified atmosphere, containing 5% CO_2_, in a specific incubator.

U266 human multiple myeloma cell line was provided by ATCC (Virginia, USA). This cell line was maintained in RPMI 1640 supplemented with 10% FBS, 2 mM glutamine and 100 U/ml of penicillin and 100 µg/ml of streptomycin. Cells grow partially (loosely) adherent and can be removed with a bent Pasteur pipette (trypsin is not necessary). The cell culture was incubated at 37 °C with 5% CO_2_ saturation.

### U266 cells nucleofection using AMAXA nucleofector

To perform transfection using RNP complex on U266 cells, the first step was to complex 80 pmol of TrueCutCas9 protein V2 (which is supplied at 5 mg/ml) (INVITROGEN, Waltham, Massachusetts) with 240 pmol of sgRNA (which is supplied at 100 μM) (SYNTHEGO), incubating at room temperature for 20 min. At the same time, adequate numbers of 6-well cell culture plates were prepared to receive cells after nucleofection. 2 mL of pre-warmed culture media (RPMI 1640 supplemented with 10% FBS, 2 Mm glutamine and 100 U/ml of penicillin and 100 μg/ml of streptomycin) were added to the wells, and the plates were kept in a cell culture incubator (37 °C, 5% CO_2_). The U266 cells were mixed to obtain single cells in suspension and collected in sterile tubes. With the aim to transfect 500,000 cells per well, U266 were counted and transferred to sterile tubes. Cells were centrifuged at 161 g for 5 min at room temperature and the supernatant was removed. Pellets were resuspended in 1 ml of sterile 1× PBS, and the resuspensions were centrifuged at the same conditions above. After discarding supernatant, cells were resuspended in 100 μl of Ingenio Solution, rebalanced at room temperature before use, supplied in the Ingenio Electroporation Kit (MIRUS BIO, Madison, WI). The previously formed ribonucleoprotein complexes were added to the cell suspension, which was transferred to a 0.2 cm sterile cuvette and placed into the Amaxa Nucleofector II device (AMAXA BIOSYSTEMS, Lonza, Basel Switzerland) using the X-005 program according to manufacturer’s protocol. The content of the cuvettes was then transferred to the plates previously set up and placed in the incubator.

### Human studies and subjects

The study (#171191), which involved skin biopsy of adult and consenting SCA1 patients, was approved on the 14th of December 2017 by Comitato Etico Unico della Provincia di Ferrara (Single Ethics Committee of the Province of Ferrara, Italy), vouching that it conforms to the European Medicines Agency Guidelines for Good Clinical Practice.

Patients have received an information sheet and were able to ask the researchers for any information about the study; finally, before undergoing the biopsy, they signed the informed consent.

### Skin biopsy

Patients underwent skin biopsy at the distal leg, 10 cm above the lateral malleolus, using a disposable 4-mm punch under sterile condition after local anaesthesia with lidocaine. The procedure does not need sutures. Specimens were transferred into 15 ml tubes containing DMEM High Glucose Medium supplemented with 20% not heat-inactivated fetal bovine serum (FBS), Antibiotic Antimycotic Solution 100×, and then taken to the laboratory to be processed.

### Fibroblasts’ isolation and culture

The skin fragments were transferred to a 10 cm diameter cell plate and further fragmented with a disposable scalpel after 30 min at 37 °C. The skin fragments were then moved into two T25 flasks with 1 ml of DMEM High Glucose Medium supplemented with 20% FBS and Antibiotic Antimycotic Solution in a cell culture incubator (37 °C, 5% CO_2_). Fresh medium was added after 48 h and at regular intervals until it reached 3–4 ml in total, after which it was replaced twice a week. After 20–25 days, fibroblasts were detached with Trypsin–EDTA 1X (EUROCLONE, Pero, Italy) and moved to a new flask. New medium was added to the plate with skin fragments and fibroblasts were periodically transferred to new flasks for at least a month. Once amplified, SCA1 patient-derived fibroblasts were frozen as passage 1.

### Transfection tests

#### Plasmid

We used pcDNA3-EGFP (pcDNA3 backbone vector expressing Enhanced Green Fluorescent Protein), that was a gift from Doug Golenbock (Addgene plasmid #13031; http://n2t.net/addgene:13031; RRID: Addgene_13031) and pcDNA3 (INVITROGEN).

#### Lipofection

Lipofection methods are described in Supplementary Materials section.

#### Nucleofection

Skin fibroblasts were subcultured 2–3 days before nucleofection, resulting in a confluency of 80–90% on the day of treatment. The nucleofection was performed as previously described, but using in this case 100 μl of NHDF Nucleofector Solution to resuspend the fibroblasts, rebalanced at room temperature before use, supplied in the Amaxa NHDF Nucleofector Kit (AMAXA BIOSYSTEMS, Lonza, Basel Switzerland). 1 μl of Alt-R Cas 9 Electroporation Enhancer 100 µM (IDT, Coralville, Iowa) and 1 μg of pcDNA3 or pcDNA3-EGFP were added to cell suspensions. The mixture was transferred to a 0.2 cm sterile cuvette and placed into the Amaxa Nucleofector II device (AMAXA BIOSYSTEMS) using the P-022 and the U-023 program according to manufacturer’s protocol, which guarantees respectively high cell viability (P-022) and high transfection efficiency (U-023). The content of the cuvettes was then transferred to the plates previously set up and placed in the incubator.

### FACS analysis

24 h after transfection or nucleofection, the fibroblasts were detached and transferred to the appropriate FACS tubes, then centrifuged at 60 g for 10 min at room temperature. After removing the supernatant, the cells were washed with 1× PBS and centrifuged again at 60 g for 10 min. Pellets were resuspended with 250 µl of PBS 1× and the samples were stored on ice until the analysis was carried out with FACScanto II (FLOW-ACTIVATED CELL SORTING, BECTON–DICKINSON, San Jose, CA), using the Cell Quest software Pro (BECTON DICKINSON). The channels that have been chosen for data collection were: FSC (Forward Scatter), SSC (Sideward Scatter) and FL1 (FL-1 Height); the latter receives data regarding the green fluorescence emitted by the GFP fluorophore (Green Fluorescent Protein).

The fluorescence histograms were set in such a way that the peak, representing not treated cells, was within the fluorescence threshold of 10^1^. In the dot plot obtained with the physical parameters FSC and SSC, a gate was drawn selecting the central portion of the cell population to exclude the dead or dying cells. Furthermore, in the dot plot having FSC on the abscissa axis and FL1 on the ordinate axis, a gate was performed to highlight the cells expressing GFP. By determining the percentage of green fluorescent cells, it was possible to evaluate the transfection efficiency in each experiment.

### Treatment of normal and SCA1 fibroblasts with RNP complex

Nucleofection was carried out as previously described, adding 100 μl of NHDF Nucleofector Solution, the Alt-R Cas9 Electroporation Enhancer 100 µM and the previously formed ribonucleoprotein complexes to the cell pellets (5 × 10^5^ cells). For nucleofection of normal and SCA1 patients the U-23 and P-022 program, respectively, were used. The cells were then incubated at 37 °C with 5% CO_2_ saturation and amplified until about 4 × 10^6^ cells, with the aim to extract total proteins, genomic DNA and RNA.

### Total proteins extraction

After detaching and counting fibroblasts, the cells were centrifuged at 60 g for 10 min at 4 °C. Cells were then washed with sterile phosphate buffer and centrifuged again at the same conditions described above. The pellets were resuspended in an adequate volume of extraction buffer (50 mM Tris–HCl pH 8.0, 1% V/V Triton X-100, 0.5% V/V Nonidet P-40, 10 mM mercaptoethanol, 4% V/V glycerol and Complete Mini Protease inhibitor cocktail tablets (ROCHE, Basel Switzerland), 1 tablet in 10 mL). The volume of the extraction buffer was related to the number of cells to be lysed (1 ml of buffer for 25 × 10^6^ cells). Five freezing cycles (in dry ice or at − 80 °C) and five thawing cycles at room temperature were alternated to promote cell lysis. The samples were centrifuged at 17,530 g for 3 min at 4 °C; the supernatants were stored at − 20 °C. Supernatants were quantified by Pierce BCA Protein Assay Kit (THERMO FISHER SCIENTIFIC, Waltham, Massachusetts). Protein extracts were used for Western blotting analysis.

### Western Blotting analyses

To detect ATXN1 proteins in U266 lines and normal fibroblasts, the anti-Ataxin 1 antibody (ABCAM, Cambridge, United Kingdom) was used, while antibodies against α tubulin (R&D SYSTEMS, Minneapolis, USA), β actin (ORIGENE TECHNOLOGIES INC., Rockville, Maryland) and GAPDH (NOVUS BIOLOGICALS, Denver, Colorado) were used as housekeeping controls; for SCA1 fibroblasts, instead, the antiserum 12NQ, kindly donated by Dr. Orr^27^ was used as primary antibody, while the normalization of the data obtained was carried out using the quantification of total proteins. 30 µg of protein extracts were denatured for 5 min at 98 °C in 1× SDS sample buffer (62.5 mM Tris–HCl pH 6.8, 2% SDS, 50 mM Dithiotreithol (DTT), 0.01% bromophenol blue, 10% glycerol), resolved by SDS-PAGE and transferred to nitrocellulose or LF-PVDF membranes (BIORAD LABORATORIES INC., Hercules, California). The U266 lines and normal fibroblasts membranes were then blocked using 1% skim milk/TBS, while the SCA1 membranes were blocked in EveryBlot Blocking Buffer (BIORAD LABORATORIES INC., Hercules, California). The membranes were then incubated with primary antibodies in their respective buffers (same used for blocking) overnight at 4 °C. After washing in 0.05% Tween 20/TBS, membranes were incubated with the corresponding secondary antibody conjugated with HRP for U266 lines and normal fibroblasts membranes, and the secondary antibody Goat Anti-Rabbit IgG StarBright Blue 700 (BIORAD LABORATORIES INC., Hercules, California) for SCA1 membranes for 1 h at room temperature and washed again.

For U266 lines and normal fibroblasts membranes, chemiluminescence signals were detected using the Clarity Western ECL Substrate (BIORAD LABORATORIES INC., Hercules, California) according to the manufacturer’s protocol. For SCA1 membranes, instead, fluorescent signal was detected using the StarBright B700 program.

The signal intensity was determined using the ChemiDoc MP Imaging System (BIORAD LABORATORIES INC., Hercules, California) for both types of membranes.

### Phenol/chloroform extraction of genomic DNA

Cell pellets were resuspended with a lysis solution (10 mM Tris HCl pH 8.0, 400 mM NaCl, 2 mM EDTA pH 8.0, 0.45% p/V SDS and 0.45 mg/mL proteinase K) and incubated at 56 °C for 2 h. One volume of phenol:chloroform:isoamyl alcohol (25:24:1) was added and mixed by inversion for 5 min. Samples were centrifuged at room temperature for 5 min at 12,879 g and then the aqueous phase was transferred to a clean tube. After a second extraction to optimize DNA purification, an equal volume of chloroform:isoamyl alcohol was added to the aqueous phase and samples were centrifuged under the same conditions described above. 2.5 volumes of 96% ethanol were added to each tube, which were placed at − 20 °C overnight to precipitate DNA. Samples were centrifuged at 4 °C for 5 min at 12,879 g, washed with 1 ml of 70% ethanol and then centrifuged again under the same conditions. The dry pellets were finally resuspended in water and the DNA quantified using the Biospectrometer (EPPENDORF, Hamburg, Germany).

### PCR

PCR reactions were set up in a final volume of 50 μl, using 400 ng of genomic DNA from each patient, 3 units of AmpliTaq Gold polymerase (THERMO FISHER SCIENTIFIC, Waltham, Massachusetts), 0.2 µM of each primer, and 200 µM of each dNTP. The annealing temperature of the amplification cycles was determined based on the melting temperatures of the primers, while the elongation time was calculated based on the length of the fragment to be amplified and the processivity of the Taq polymerase. The primers used for PCR and their melting temperatures are reported in Table S2. PCR products were purified using NucleoSpin^®^ Gel and PCR Clean-up (MACHEREY–NAGEL, Düren, Germany), according to manufacturer’s protocol.

### NGS analyses

To detect on-target modifications, the genomic regions flanking sgRNAs target sites were amplified and barcoded employing two successive rounds of PCR in modified nested PCR approach^28–30^.

A total of 400 ng of genomic DNA were amplified as previously described. PCR conditions were 95 °C for 10 min; 30 cycles of 95 °C for 15 s, 57 °C for 30 s, 72 °C for 45 s, and a final extension at 72 °C for 5 min. The primers used in the first step were designed with standard Illumina overhang adapter sequences, specific stagger sequences (to ensure sequence diversity for an accurate base calling), and locus-specific primers (Table S3). Each forward or reverse primers mix contains three different primers which differ in the stagger sequence.

PCR products were purified with NucleoSpin Gel and PCR Clean-up (MACHEREY–NAGEL, Düren, Germany), then quantified by Biospectrometer (EPPENDORF, Hamburg, Germany), and checked on agarose gel.

Barcodes for NGS libraries are reported in Table S5, in supplementary Material section. About 2.8 × 10^11^ molecules from purified PCR product were barcoded in a limited cycle amplification step using Nextera XT Index Kit primers (ILLUMINA, San Diego, California). Reactions were carried out in a 50 μl reaction: 3 units of AmpliTaq Gold polymerase (THERMO FISHER SCIENTIFIC, Waltham, Massachusetts), 1 µM of each adapter (Table S5), and 200 µM of each dNTP. Samples were barcoded using the following program: 95 °C for 30 s; 8 cycles of 95 °C for 10 s, 55 °C for 30 s, 72 °C for 30 s, and a final extension at 72 °C for 5 min.

After purification and quality control, libraries were normalized and pooled. The pool was quantified using qPCR (KAPA Library Quantification Kit Illumina Platforms) and sequenced at 10–12 pM and 10% PhiX with Illumina MiSeq using MiSeq Reagent Kit PE250 on a MICRO flowcell.

The amplicon of *BCOR*, *INNP5A*, *ZNRF1*, *COL3A1*, *KCNQ1* and *MEIS1* genes were prepared by PCR using the primers shown in Table S2, while the libraries preparation was performed by GENEWIZ Europe (Leipzig, Germany). A maximum of 500 nt of PCR products were sent to the company and the raw data were processed by bioinformatic analysis.

### Statistical analysis

Statistical analyses were performed using GraphPad Prism 7.00 software. The statistical tests applied were unpaired t test with two-tailed P value and alpha level P < 0.05; F test to compare variances with alpha level P < 0.05.

### Software

For this study the following software and web tools were used: CRISPR Design software, developed by Zhang at the MIT Laboratory in 2015^26^, and CRISPR design tool from SYNTHEGO, for the design of sgRNAs; Primer-BLAST (NCBI), for the design of primers to be used in PCR reactions; Verify Guide Design (SYNTHEGO) for the identification of off-target sites of G3 and G8 sgRNAs; Galaxy platform^31^ and Integrative Genomics Viewer (IGV) tool^32^ for on-target NGS analysis; GraphPad Prism 7.00, for statistical analysis.

## Results

### Design and in vitro screening of sgRNAs

**Figure 1:**
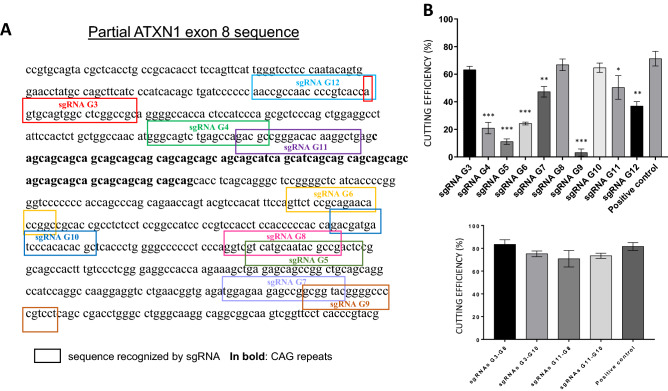
Design and in vitro screening of sgRNAs for *ATXN1* gene. (**A**) sgRNAs were designed using the CRISPR design software by Zhang lab^19^. Ten sgRNAs capable of cutting upstream and downstream of the polyQ tract were identified. (**B**) In vitro screening tests were performed to evaluate the sgRNAs cutting efficiency, using Guide-it sgRNA In Vitro Transcription and Screening System (CLONTECH). (**C**) The most efficient sgRNAs were then tested in pairs. The positive control is supplied by the kit and is designed to achieve high efficiency DNA double helix cleavage. For this reason, it was used as a comparison for the relative quantification of the cleavage efficiency of the designed sgRNAs. Values are mean ± s.e.m. from three independent experiments. *p < 0,05; **p < 0,005; ***p < 0,001. Samples G3, G8 and G10 confirm the null hypothesis that there is no significant difference in cutting efficiency compared to the positive control provided by the kit.

To design specific and efficient sgRNAs, several tools have been developed over the last few years, capable, once the target region has been inserted, to identify sgRNAs with great on-target cutting efficiency and low off-target cutting capacity^26^. Using the CRISPR design software developed by the Zhang lab^26^, we designed ten different sgRNAs able to recognize sequences upstream and downstream the *ATXN1* polyQ tract (Fig. 1A). Considering that the theoretical efficiency of the sgRNA, determined by bioinformatics analyses, does not always coincide with the real capacity to recognise and mediate the cutting by Cas9, we decided to produce our designed sgRNAs by in vitro transcription and test them both setting up in vitro target template cleavage reactions using Cas9-sgRNA ribonucleoprotein complexes and in immortalized U266 cell line, as a cellular model. These cells were chosen because they express ATXN1 at high level with respect to other immortalized cell lines, as reported by The Human Protein Atlas (https://www.proteinatlas.org/ENSG00000124788-ATXN1/celltype) (Fig. S1).

The in vitro screening of sgRNA involves the production of a template of about 2000 bp, containing the protospacers of the sgRNAs we designed, which were produced by in vitro transcription and once complexed with Cas9 used to mediate the cleavage of the template DNA. The efficiencies shown in Fig. 1B were determined as a percentage of the fragments obtained from the Cas9 cut related to the total amount of the target template (Fig. S2A). Four sgRNAs, two capable of recognizing sequences upstream and two downstream of the polyQ tract, show a high capacity to mediate the template cleavage (G3, G8, G10, G11). Since a multiple cut of the *ATXN1* gene can determine loss of genetic material and therefore a more efficient silencing of the gene itself^33^, we set up four pairs of sgRNAs, whose recognition and cutting efficiency was always very high (Figs. 1B; S2B). After having transfected the nucleoprotein complexes in a U266 cell line, in which we evaluated the cutting efficiency of six different sgRNA pairs (G3-G8, G3-G7, G3-G10, G11-G8, G11-G7, G11-G10) setting up Western Blotting analysis of ATXN1 expression, the G3–G8 and G3–G10 sgRNA pairs confirmed their ability to effectively suppress *ATXN1* gene expression (Fig. S3), while the G11–G7 and G11–G10 pairs seem to increase the expression of the protein. Regarding the result with these last two combinations of sgRNA we have no explanation as to why they increase the expression of the protein and further experiments will have to be done to clarify this point.The choice of sgRNAs to be used depended on the position of their protospacers on the sequence, leading us to select the pair with the most distant targets (G3–G8) (Fig. 1A).

### Isolation and characterization of SCA1 fibroblasts

We have decided to use, as a cellular model, dermal fibroblasts of SCA1 patients for the ex vivo evaluation of our silencing approach. In this way we were able to evaluate the effectiveness of our CRISPR/Cas9 system in the presence of different expansions of the polyQ tract, from 42 to 67 nucleotides, trying to understand if its length could also affect the efficiency of the system. Furthermore, since different cells can respond differently to the same treatment, this has allowed us to evaluate how cellular variability can affect our gene silencing approach. Fibroblasts were isolated from a 4 mm skin fragment, obtained, after the signature of the informed consent, from 12 patients with different polyQ expansion in mutated *ATXN1* gene: SCA1N1 (50/28 repeats), SCA1N5 (45/28), SCA1N6 (42/28), SCA1N8 (60/30), SCA1N9 (51/28), SCA1N10 (46/30), SCA1N11 (48/28), SCA1N12 (43/30), SCA1N14 (58/32), SCA1N16 (53/34), SCA1N17 (67/25) and SCA1N19 (45/26).

**Figure 2:**
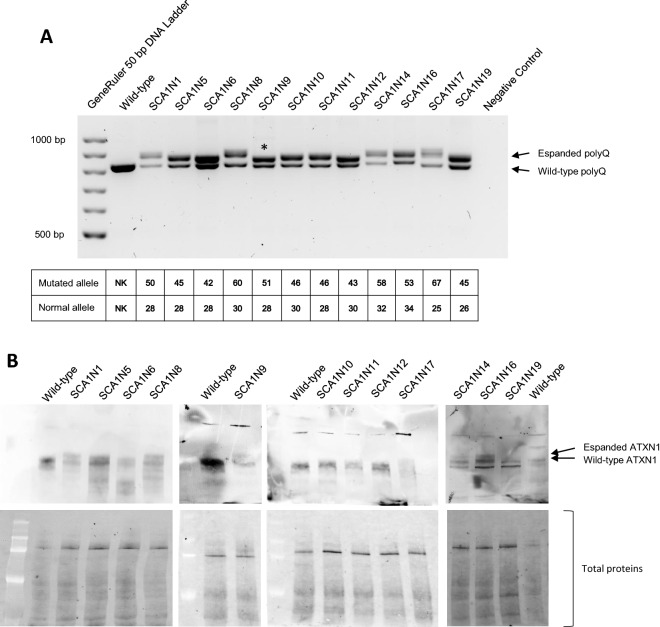
Characterization of SCA1 fibroblasts. (**A**) Amplification by PCR of the *ATXN1* exon 8 containing the polyQ tract, using the genomic DNA extracted from the five SCA1 patients’ fibroblasts as template. The negative control consists of a PCR reaction in the absence of template DNA, replaced by water. In the table are reported the triplet numbers of the two alleles, mutated and normal, obtained from the patients’ genetic analysis. (**B**) The upper membranes are related to the ATXN1 expression in SCA1 fibroblasts, using 12NQ antiserum as anti-ATXN1 antibody. The bottom membranes are related to the total amount of proteins for each sample. * Discrepancy between the data we obtained, and the result of the genetic analysis carried out on the patient SCA1N9, not due to the methods we used to verify the presence of the two alleles. DNA ladder: GeneRuler 50 bp DNA Ladder (50 bp, 100 bp, 150 bp, 200 bp, 250 bp, 300 bp, 400 bp, 500 bp, 600 bp, 700 bp, 800 bp, 900 bp, 1000 bp).

To confirm the presence of an *ATXN1* allele with pathological increase in CAG triplets we amplified by PCR the exon 8 of the *ATXN1* gene and verified, by electrophoresis on agarose gel, the presence of two PCR fragments, corresponding to the healthy and the mutated allele (Fig. 2A). At the same time, to verify the production of the normal and expanded polyQ ataxin 1, a Western Blotting experiment was set up, where, using anti-ATXN1 antiserum 12NQ, it was possible to highlight the presence of both ATXN1 proteins (Fig. 2B).

### Identification of the best method of transfection for normal and SCA1 fibroblasts

Several studies demonstrated that, in cultured mammalian cells, the delivery of ribonucleoprotein complexes composed of sgRNA and Cas9 have numerous advantages, including very fast editing, capable of reaching a plateau within 24 h, and a rapid elimination of Cas9, resulting in reduced off-target effects^34–36^. Fibroblast primary cultures are difficult to transfect, and no protocol has been validated so far. Some groups have already evaluated the different transfection methods for wild-type fibroblasts by comparing lipofection with nucleofection, demonstrating that the former did not give appreciable results while the latter is very efficient^37,38^. Therefore, we decided to confirm these data on wild-type cells and to verify them on SCA1 fibroblasts, evaluating the transfection efficiency of several lipofection reagents, including Lipofectamine 2000 (INVITROGEN), TransIT-X2 (MIRUS), Effectene (QIAGEN), jetPRIME (POLYPLUS), X-tremeGENE (ROCHE), NanoJuice (MILLIPORE) and Fail-Safe L5000 (LEGENE BIOSCIENCES), and the Nucleofector Technology.

Although we chose to deliver the CRISPR/Cas9 system in the form of a ribonucleoprotein complex, we decided to evaluate the transfection efficiency of SCA1 fibroblasts using a GFP-expressing plasmid for convenience, as our aim was to identify the best method of transfection. For this reason, it was sufficient to always use the same molecule with all transfection systems to be tested.

We used a plasmid containing the *GFP* gene under the control of the CMV promoter and we initially transfected it into normal fibroblasts (AG21802, AG21754, AG21805) using the lipofection reagents shown in Table S4, with the relative DNA-reagent ratios indicated therein, to test more than one experimental condition. Following FACS analysis of the fluorescence emitted by the cells 24 h after transfection, we calculated a very low transfection efficiency for all the lipofection reagents used, in all cases much less than 10% (Fig. S4). The most efficient reagents were Trans-IT X2 (5.7%) and Effectene (5.5%), while JetPRIME and X-TREME have had no effect. The same reagents used with SCA1 fibroblasts (SCA1N1, SCA1N5 and SCA1N6) were found to be either too toxic or totally ineffective (data not shown).

**Figure 3:**
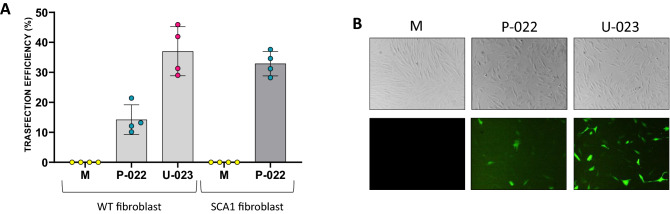
Evaluation of nucleofection efficiency in normal and SCA1 fibroblasts. The GFP-expressing plasmid was transfected into normal (AG21802, AG21754, AG21805) and SCA1 fibroblasts (SCA1N1, SCA1N5 and SCA1N6) by nucleofection using two different Amaxa Nucleofector Program, one with high viability (P-022) and the other with high efficiency (U-023). The latter proved to be too toxic for the SCA1 fibroblasts which after 24 h had a mortality greater than 90%. (**A**) The data, obtained by FACS analysis, represent the average of the experiments carried out on three cell lines of normal fibroblasts (AG21802, AG21754, AG21805) and on three lines of SCA1 fibroblasts (SCA1N1, SCA1N5, SCA1N6). (**B**) GFP expression in normal fibroblasts after 24 h from nucleofection, evaluated under fluorescence microscope. Values are mean ± s.e.m. from three independent experiments.

The Amaxa Nucleofector has different types of nucleofection programs according both to the type of cell to be transfected and to the desired efficiency or cell viability. As for human dermal fibroblasts there are two programs, one with high viability (P-022) and one with high transfection efficiency (U-023), we tested both programs on three normal and SCA1 fibroblasts (both above mentioned), obtaining significantly better transfection efficiencies compared to lipofection, after FACS analysis (Fig. 3A,B). SCA1 fibroblasts were found to be much more sensitive to nucleofection so that with the high-efficiency program they had a mortality higher than 90%, while with the high-viability program the transfection efficiency (32.3%) was similar to that obtained with the U-023 program from normal fibroblasts (37%).

### Validation of CRISPR/Cas9-based therapeutic strategy in normal and SCA1 fibroblasts

Given the results of the transfection tests, we decided to use the high efficiency and the high viability program for the nucleofection of the ribonucleoprotein complex into normal and SCA1 fibroblasts, respectively. Fibroblasts were transfected simultaneously with G3 and G8 sgRNA, which recognize sequences upstream and downstream of the polyQ tract respectively, and with a negative control sgRNA (scramble), as nucleofection control, previously associated with the endonuclease Cas9 to obtain ribonucleoproteins (RNPs). ATXN1 expression in fibroblasts from three different normal subjects (AG21802, AG21754, AG21805) treated with RNP complexes was evaluated by Western Blotting using the anti-ATXN1 monoclonal antibody and normalized against GAPDH protein amount (Fig. S5A). Following densitometric analysis of the bands obtained, it was possible to determine the percentage of suppression of the ATXN1 protein in the treated compared to the untreated cells, which consists of 47% for AG21802, 61% for AG21754 and 57.6% for AG21805 (Fig. S5B,C).

**Figure 4:**
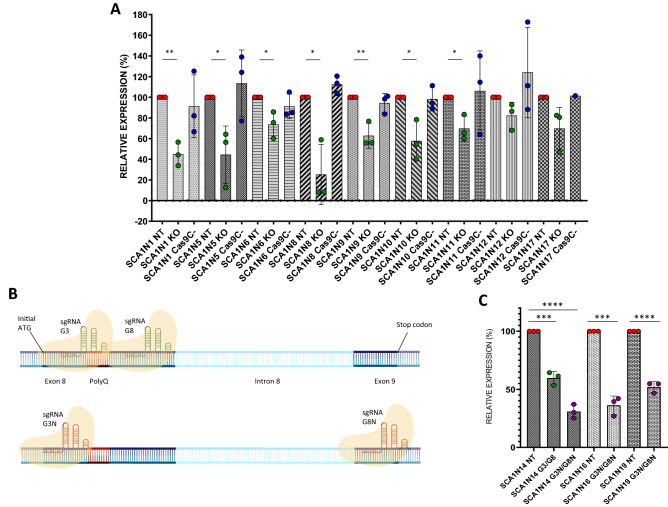
Effects of CRISPR/Cas9 systems in SCA1 fibroblasts. (**A–C**) ATXN1 expression in SCA1 fibroblasts. (**A**) Fibroblasts from nine SCA1 patients were treated using sgRNAs G3 and G8 complexed with Cas9 endonucleases. (**B**) Mapping of the sgRNAs’ target sequences: the sgRNAs G3 and G8 recognize target sequences respectively upstream and downstream of the polyQ tract, while the targets of G3N and G8N lie at the beginning of exon 8 and exon 9, respectively (created with BioRender.com). (**C**) SCA1N14 fibroblasts were treated using both sgRNAs G3 and G8 and sgRNAs G3N and G8N complexed with Cas9 endonucleases. SCA1N16 and SCA1N19 fibroblasts were treated using sgRNAs G3N and G8N complexed with Cas9 endonucleases. The ATXN1 expression was determined by Western Blotting. ATXN1 abundances were expressed relative to total proteins, determined by densitometry. Values are mean ± s.e.m. from three independent experiments. The statistical test used was unpaired t test with two-tailed P value and alpha level P < 0.05. *p < 0.05; **p < 0.005; ***p < 0.0005; ****p < 0.0001.

Different SCA1 fibroblasts were then treated with RNP complexes G3sgRNA/Cas9, G8sgRNA/Cas9 and scramble sgRNA/Cas9 (Cas9 C-), as negative control. Since the anti-ATXN1 monoclonal antibody previously used was not able to bind mutated ATXN1 proteins, the Western Blotting analysis was set up using anti-ATXN1 antiserum 12NQ, kindly donated to us by Harry Orr, and total protein amount for normalization (Fig. S6). The data obtained showed significant reduction of the ATXN1 expression in seven out of nine cases (from 25.1 to 82.3% of expression compared to not treated cells) (Fig. 4A). The antiserum 12NQ, raised against the 200 amino acid N-terminal portion of ATXN1, can recognize truncated forms of the protein obtained after gene editing treatment. The absence of lower molecular weight bands in treated compared to untreated samples allows us to state that our CRISPR/Cas9 system does not cause the production of truncated proteins. Only in the case of the fibroblasts of the SCA1N10 patient it is possible to notice, following the treatment, the increase of an isoform of lower molecular weight, present however also in the untreated samples (Fig. S6M).

To understand if the distance between the two cutting sites can affect the silencing efficiency, we decided to design two new sgRNAs using Synthego’s CRISPR Design Tool: the G3N and G8N sgRNAs recognize the *ATXN1* gene respectively in 5′ of exon 8 and 5′ of exon 9, as shown in Fig. 4B.

To verify whether the two new RNA guides possessed greater suppression efficiency, we treated patient SCA1N14’s fibroblasts in parallel with G3/G8 and G3N/G8N sgRNAs (Fig. S7A,B). Comparing the expression of the ATXN1 protein in the transfected SCA1N14 fibroblasts with both the old and the new RNPs, it is evident how the sgRNAs G3N and G8N determine a marked increase in the suppression of this protein (from 59.7 to 30.8% of expression compared to not treated cells) (Fig. 4C).

Once we verified that the new sgRNAs were more efficient in silencing the *ATXN1* gene, we decided to treat two other different SCA1 fibroblasts with RNP complexes G3NsgRNA/Cas9 and G8NsgRNA/Cas9 (Fig. S7C–F). Data obtained after treatment of SCA1N16 and SCA1N19 fibroblasts show a significant suppression of ATXN1 protein expression of 63.8% and 48% respectively compared to untreated cells (Fig. 4C).

**Figure 5:**
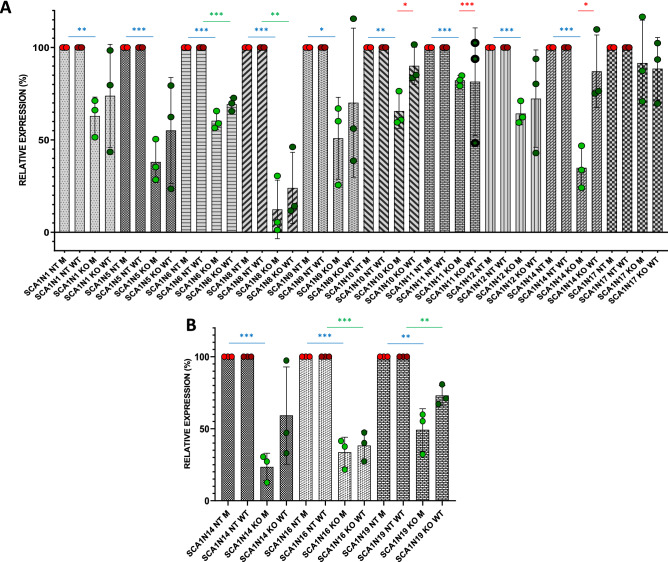
Effects of the CRISPR/Cas9 system on the differential expression of healthy and mutated ataxin 1 protein. Healthy and mutated ATXN1 protein expression was determined in SCA1 patient cells treated with both G3-G8 (**A**) and G3N-G8N (**B**) sgRNAs complexed with Cas9 endonucleases. The bands produced by the two forms of ataxin 1 obtained following Western Blotting analysis were separately quantified by densitometry and both normalized on the total amount of proteins. Values are mean ± s.e.m. from three independent experiments. The statistical test used was unpaired t test with two-tailed P value and alpha level P < 0.05. *p < 0.05; **p < 0.005; ***p < 0.0005.

To understand if the spacer pairs were able to recognize the mutated and the healthy allele with different efficiency, we decided to separately analyse the bands related to proteins with expanded polyQ from those not mutated, by means of densitometric quantification. The data in Figs. 5A and S8A show a propensity of the G3sgRNA/Cas9 and G8sgRNA/Cas9 complexes to preferentially silence the mutated allele compared to the healthy one, resulting in a high significance of silencing of the protein with expanded polyQ in all samples except SCA1N17, as evidenced by the previous analysis. As for the healthy protein, the significance is present only in two samples (SCA1N6 and SCA1N8). On the contrary, the complexes with the two new sgRNAs G3N and G8N, although they proved to be more efficient in suppressing both proteins, do not seem to show greater selectivity for the mutated allele (Figs. 5B and S8B).

### Evaluation of on-target gene modifications

To verify which genetic alterations were introduced by our CRISPR/Cas9 systems, we decided to perform NGS analyses. We thus produced libraries of DNA fragments obtained from the amplification of the regions containing the cleavage sites of the RNP complexes (Fig. S9), using genomic DNA isolated from the transfected fibroblasts of five patients, for one of which we considered two treatments with G3sgRNA/Cas9 and G8sgRNA/Cas9 and one with G3NsgRNA/Cas9 and G8NsgRNA/Cas9, for a total of seven samples (SCA1N5, SCA1N10, SCA1N14 1 T and SCA1N14 2 T for G3 and G8 sgRNAs, and SCA1N14, SCA1N16 and SCA1N19 for G3N and G8N). The analysis of the double treatment of SCA1N14 cells with G3-G8 sgRNA/Cas9 RNPs aimed to verify the reproducibility of the results. The region containing the protospacers of the G3, G8 and G3N sgRNAs was amplified using two different pairs of primers (pG3Nv2_F/pG8_R and pG3_F/pG8B_R), while the pG8N_F/pG8N_R pair was used for the region containing the G8N protospacer. Furthermore, to understand if the treatment with the G3N/G8N-Cas9 complexes had caused the loss of the DNA fragment between the two cleavage sites of about 21 kb, we set up a PCR using the primers pG3Nv2_F and pG8N_R, which anneal respectively upstream of the G3N protospacer and downstream of the G8N one, obtaining an amplicon of 274 bp if fragment loss occurs between the two protospacers.

**Figure 6:**
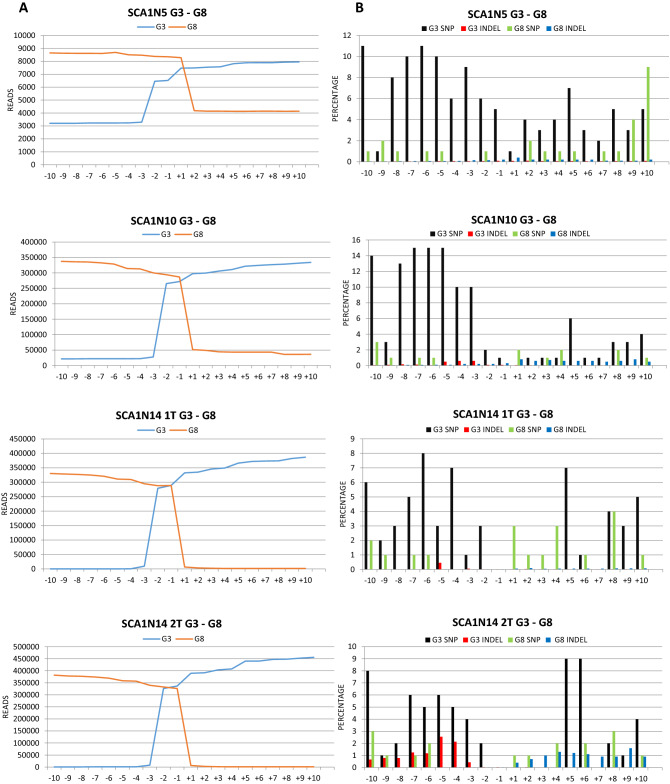

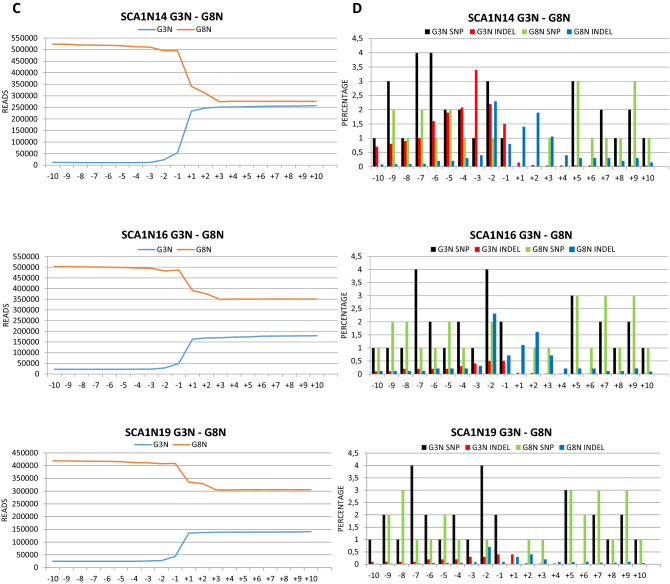
On-target NGS analysis. (**A**,**B**) represent the total reads count (**A**) and SNPs and indels percentages (**B**) related to the region between 10 nt upstream and 10 nt downstream of the cutting sites in fibroblasts (SCA1N5, SCA1N10, SCA1N14 1 T and SCA1N14 2 T) treated with G3/G8sgRNA-Cas9. (**C**,**D**) represent the total reads count (**C**) and SNPs and indels percentages (**D**) in fibroblasts (SCA1N14, SCA1N16 and SCA1N19) treated with G3N/G8NsgRNA-Cas9.

In three of the four samples treated with the G3sgRNA/Cas9 and G8sgRNA/Cas9 RNPs the PCRs carried out to produce the amplicons generated, in addition to the expected amplicons of 762 bp (pG3Nv2_F/pG8_R) and 603 bp (pG3_F/pG8N_R), two smaller fragments, 381 bp and 222 bp long, respectively, such as it would be expected in the case of loss of genetic material between the two cleavage sites (Fig. S10). This was confirmed by the NGS analysis which highlighted a strong reduction in the total read count between the two cleavage sites in SCA1N10, SCA1N14 1 T and SCA1N14 2 T samples (Figs. 6A and S11C–H), although the NGS shows a clear reads reduction also in the SCA1N5 sample, whose smaller bands were not visible in Fig. S10, probably due to the more limited number of amplicons produced (Fig. S11A,B). However, it was not possible to determine an exact percentage of the DNA fragment loss between the two protospacers, as during the Nested PCR required to produce the libraries, the smaller fragment was amplified more efficiently then the larger one (Fig. S10). As expected, in the case of the samples treated with G3NsgRNA/Cas9 and G8NsgRNA/Cas9, the amplification with pG3Nv2_F/pG8_R did not lead to the production of small DNA fragments, since only the G3N protospacer is present in the region amplified by these primers. On the contrary, the use of pG3Nv2_F/pG8N_R primers led to the generation of the fragment of 274 bp, expected in the case of loss of the region of about 21 kb between the two cutting sites, in all three analysed samples (Fig. S10). This conclusion has been amply confirmed by the NGS analysis (Figs. 6C and S11I–P). Also in this case, it was not possible to determine a percentage of deletion of this region, since the areas containing the G3N and G8N protospacers were amplified using different pairs of primers. The same number of molecules of all the obtained amplicons was then mixed in the pool. To determine a percentage, we would have had to amplify the whole region of the gene starting from the G3N cleavage site to that of G8N, with the consequent production of two fragments, one of about 21 kb and the other 274 bp, containing the deletion. However, this is not feasible with this type of NGS analysis, which allows the evaluation of only small amplicons.

Beyond these large deletions, numerous SNPs, in some cases up to 15%, and a very few small indels, with a maximum of 3.4% in the case of the cut mediated by G3N, were introduced in all samples, in the region between 10 nucleotides upstream and 10 downstream of the cleavage sites (Fig. 6B,D). Analyses to identify variant regions were performed on the cloud, using the Galaxy platform^31^. In particular, the raw reads of the single samples were aligned to the reference genome (GRCh38) using the BWA-MEM algorithm^39^. Subsequently, the variants were called with the FreeBayes algorithm^40^. Both algorithms were used with the default parameters.

### Evaluation of off-target effects

One potential complication of gene editing is that the engineered nuclease could create other, unintended genomic changes. This off-target activity of the nuclease occurs fundamentally because the Cas9 lacks perfect specificity. Numerous tools have been developed to identify potential off-target editing sites of CRISPR/Cas9 systems, even if none of them seem to be sufficiently accurate in predicting the low-frequency off-target effects^41^. The exclusive use of these tools is not sufficient to make an accurate prediction of the off-target effects of a possible RNP complex. Therefore, it is possible to associate this analysis with an amplicon-based next-generation sequencing, to evaluate the true off-target effects at predicted candidate sites.

In this work we therefore wanted to confirm whether by coupling an in silico tool with an experimental analysis it was possible to effectively identify potential off-target effects. As this is proof-of-principle work, we decided to carry out this evaluation only on G3 and G8 sgRNAs.

**Figure 7:**
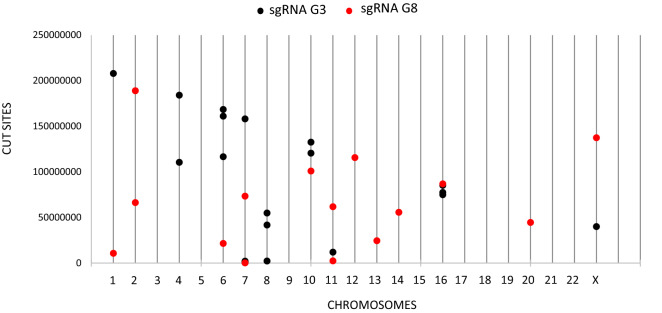
Predictable G3 and G8 sgRNA off-targets. The predictable off-targets were identified using the guide design verification tool available on the SYNTHEGO website. Their chromosomal distribution is shown in the figure.

To identify the predictable off-targets of G3 and G8 sgRNAs, we used the guide design verification tool available on the SYNTHEGO website, which identified seven possible off-targets for G3 sgRNA that differ by only three mismatches from the target sequence, of which six are contained in genes, and 142 off-targets that differ by four mismatches; while for the G8 guide only 15 off-targets with four mismatches were identified. Some of these non-specific cutting sites are shown in Fig. 7.

To verify if the RNP complexes were able to recognize and significantly cut at the level of off-target sites, we decided to analyse their cutting efficiency in three genetic *loci*, different from the *ATXN1* gene, for each sgRNA, by amplicon NGS, in three out of four genes for each sgRNA: *BCOR*, *INNP5A* and *ZNRF1* genes for sgRNA G3 (Fig. S12A–F) and *COL3A1*, *KCNQ1* and *MEIS1* genes for G8 (Fig. S12G–N). Analyses to identify variant regions were performed as previously described.

**Figure 8:**
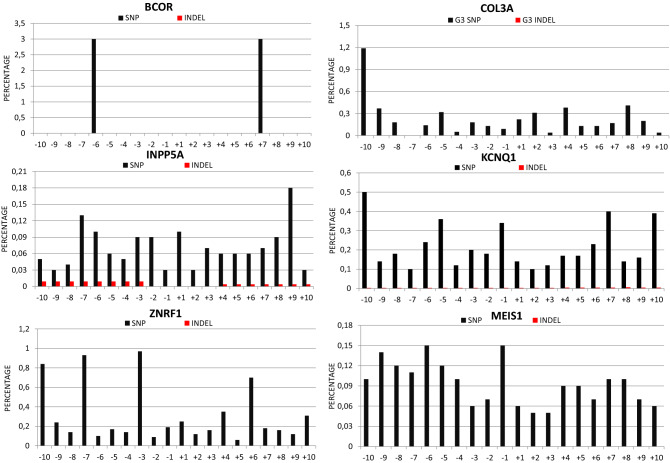
Off-target NGS analysis**.** Graphs represent the SNPs and indels percentages related to the region between 10 nt upstream and 10 nt downstream of the predicted G3 and G8 off-target cutting sites.

For all genes, the results obtained by NGS confirm the absence of off-target effects, except for *BCOR*, for which the number of target reads was too low for a significant analysis (Figs. 8; S12A,B).

## Discussion

SCA1 is one of the two spinocerebellar ataxias with the highest incidence in Italy, especially in the North, with a frequency of about 21%^42^. There is no cure for SCA1, and current therapies only provide symptomatic relief. Although several research teams are trying to develop therapeutic strategies that can silence the *ATXN1* gene and block the production of the toxic protein, none of these approaches have entered clinical trials, neither in Europe (https://www.clinicaltrialsregister.eu/) nor in the United States (https://clinicaltrials.gov).

**Figure 9:**
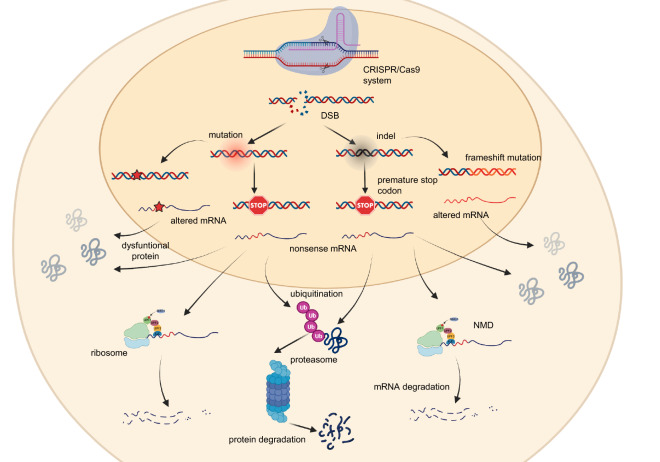
Cellular mechanisms activated following DSB. Following the creation of the DSB in the cleavage site by the RNP complex, the cell activates two possible mechanisms in order to repair the double helix: homologous recombination (HR) and non-homologous end-joining (NHEJ). The latter is activated more frequently than HR, and, being an error-prone mechanism, can cause the introduction of indels or mutations in the target gene. In the first case, if the indels are multiples of three, an in-frame mutation is created with the addition of one or more aminoacids that can alter the characteristics of the protein, such as reducing its functionality or its stability. Conversely, if the indels are not multiples of three, a frameshift mutation is introduced with the establishment of a premature stop codon. The nonsense transcript produced can face two fates: if it is recognized by the nonsense mediated decay (NMD) it is degraded, otherwise it is translated into a truncated protein, which in most cases is ubiquitinated and degraded by proteasome. In the case of the introduction of point mutations, these can be silent, for example in the case they concern the third nucleotide of a codon—nonsense, when they establish a premature stop codon—or missense, in the case they determine the change of an aminoacid. Nonsense mutations can be recognized by NMD with consequent degradation of the transcript or give rise to truncated proteins, while missense mutations can cause the production of an altered protein (created with BioRender.com).

Several studies have shown that inhibition of *ATXN1* mRNA translation using siRNA and ASO leads to the recovery from SCA1 symptoms, although not permanently^15^, therefore, genetic approaches aimed at permanently silencing the *ATXN1* gene seem to be the most promising strategies for the treatment of SCA1. Since it has already been shown that specific CRISPR/Cas9-mediated gene editing could be used to permanently eliminate polyglutamine expansion-mediated neuronal toxicity^22,43^, we successfully developed a CRISPR/Cas9-based approach to efficiently reduce the production of both healthy and mutated ATXN1 protein as shown in Fig. 9. Following the DSB, if an indel involving a few nucleotides not multiple of 3 is inserted, a frameshift mutation is generated with the establishment of a premature stop codon. Based on the location of this codon, the transcript can be recognized as nonsense and therefore degraded by nonsense mediated decay (NMD). If not, the mRNA is translated into a truncated protein, which undergoes ubiquitination and degradation by the proteasome. In the case of indels that are multiples of 3, the protein produced could be less functional or more unstable. If, on the other hand, the cutting of the double helix causes the introduction of point mutations, these can either generate altered proteins or introduce a premature stop codon, which can follow the same pathways described above.

Considering that our first approach involves the use of two different sgRNAs targeting exon 8 of the *ATXN1* gene^33^, one of our goals was to identify the best transfection method for SCA1 fibroblasts, as so far no data has been published on this topic. Although our aim was to deliver the ribonucleoprotein complex, we decided to use a plasmid expressing GFP, for ease of use, greater signal detection and reduced costs. Furthermore, using a Cas9 fused with a GFP would not have provided us with more information on the transfection efficiency of our RNP complexes due to its greater steric hindrance. Even though various methods of RNP complexes delivery have been developed in recent years, such as Lipofectamine CRISPRMAX^44^, these are always based on chemical methods of transfection (e.g. lipofection) to which fibroblasts, in particular SCA1 fibroblasts, are refractory, which is why we excluded their use from our experiments. Nucleofection has proved to be the most efficient transfection method for our cells and, despite it is not applicable in vivo, it allows us to obtain very realistic data relating to the treatment with our CRISPR/Cas9 system, both in terms of efficiency and specificity, especially in anticipation of an in vivo delivery of ribonucleoprotein complexes using non-viral vectors. In recent years, many research groups have developed numerous Cas9 RNP delivery systems, to be used in future clinical trials^45–47^. This would avoid the use of viral vectors, that although might seem the most efficient delivery method both ex vivo and in vivo^34^, present numerous disadvantages, including the difficulty of construction and production, the high and continuous expression of the RNP complex, with a consequent high risk of off-target effects^35,36^, and, last but not least, the potential risk of insertion into the genome, based on the type of viral vector used.

After choosing nucleofection as the transfection method, we delivered our RNP complexes (G3 sgRNA/Cas9 and G8 sgRNA/Cas9) in normal and SCA1 fibroblasts, deciding to evaluate the effects of our approach on the entire cell population, without making any selection of the transfected cells. While this choice does not allow us to have precise information on the actual gene silencing efficiency of our CRISPR/Cas9 approach, it does give us a more precise idea of what could happen in vivo.

Treatment with the G3 and G8 sgRNA/Cas9 complexes resulted in significant suppression of the ATXN1 protein in normal and in seven out of nine SCA1 fibroblasts, although it is possible to notice a reduction of the expression also in the remaining samples. By sequencing the G3 and G8 protospacers, we excluded that the differences in efficiency were due to single nucleotide polymorphisms or other silent gene alterations present in the patients' genome (Fig. S13). The different capacity for gene silencing reinforces the concept of variability between cells, which confirms on the one hand the importance of carrying out these types of studies in samples isolated from different individuals and, on the other hand, the need to develop a personalized gene editing approach.

Despite having obtained good ATXN1 protein suppressions, we decided to evaluate if increasing the distance between the two RNPs could increase the silencing efficiency to understand if the last one was influenced by the distance between the two cutting sites. Therefore, we designed two new RNA guides capable of recognizing sequences more distant from each other, to minimize the steric hindrance of the two sgRNA/Cas9 complexes (Fig. 4B). To verify our hypothesis, we first tested G3-G8 and G3N-G8N pairs in SCA1N14 fibroblasts, to compare their efficiency, and then we treated two SCA1 fibroblasts isolated from patient SCA1N16 and SCA1N19 with the new sgRNAs only (Fig. S7). From the densitometric analysis, it emerges that the new pair of sgRNAs determines a significantly higher percentage of protein suppression, confirming that a correct distance between the RNP complexes can reduce the competition between them and therefore allow greater cutting efficiency (Fig. 4C).

Although previous studies have shown that the total loss of healthy ataxin 1 protein in mouse models Atxn1^−/−^ leads to complications such as the reduction of the transcription factor Capicua^48^, the increase of the expression of beta-secretase 1 (*BACE1*)^49^ and the reduction of hippocampal neurogenesis^50^, it has recently been shown that such problems do not occur when the suppression of the protein is only partial^51^. Even if our sgRNAs have been designed to recognize both *ATXN1* alleles, we have found that they appear to favour the suppression of the mutated allele (Fig. 5). Although these findings require further validation, they not only lay the basis for the use of the CRISPR/Cas9 system for the treatment of SCA1, but also represent a starting point for the development of a personalized therapeutic strategy.

Our CRISPR/Cas9 systems determined the deletion of the gene sequence covered between the cleavage sites, as evidenced by the NGS analysis and the data presented in Fig. 6A,C. Furthermore, several small gene modifications occurred close to the cleavage sites, with a greater frequency at the level of the G3 (Fig. 6B) and G3N (Fig. 6D). The high rates of protein suppression are likely due to the large deletions that result in the loss of larger fragments of the *ATXN1* gene. These deletions cannot lead to the formation of stable truncated proteins, as confirmed by our Western Blotting experiments. Since the double-cut of the *ATXN1* gene, with both pairs of sgRNAs, leads to an in-frame deletion (of 127 triplets for G3 and G8 and of 538 triplets for G3N and G8N), the results obtained indicate whether the repair of the DNA double-strand break by the NHEJ was precise, without further loss of genetic material, or not, with the establishment of a frameshift mutation, the loss of the fragment of DNA between the two cleavage sites led to the production of truncated proteins recognized and degraded by the ubiquitin–proteasome system. A comparable result to this was obtained by Dabrowska and colleagues in their study on the huntingtin gene^25^.

Although the CRISPR/Cas9 system has a minimal incidence of off-target effects^52^, variable levels of the latter have been observed^53–55^. The Cas9 endonucleases produced in recent times are engineered to increase their efficiency towards the target sequence, minimizing the off-target effects. Furthermore, accurate design of sgRNAs and the direct delivery of the ribonucleoprotein complex, whose short half-life considerably reduces the exposure time of the cell genome to the action of the CRISPR/Cas9 system, can significantly reduce any off-target effects^35^.

From the analysis of amplicon NGS, it emerged that the off-target effects of our G3/Cas9 and G8/Cas9 RNPs are minimal and not significant.

In conclusion, in this study we demonstrate to successfully reduce protein expression from SCA1 patient-specific cells using the CRISPR/Cas9 system, obtaining suppression efficiencies that agree with those obtained by Friedrich et al. when treating Atxn154Q/2Q mice with the antisense oligonucleotide ATXN1 ASO353^15^, but with the advantage of permanent suppression, without the need of continuous administration. We have also shown how cellular variability, and not just genetic variability, can affect the efficiency of gene editing. Finally, the comparison between two different pairs of sgRNAs led us to deduce that the greater the distance between the two cleavage sites, the greater the gene silencing efficiency, due to the smaller steric hindrance of the two RNP complexes that lead the simultaneous cleavage at the two protospacer sites. However, our therapeutic approach will be further validated in SCA1 patient-derived iNeurons and mouse model.

The CRISPR/Cas9 system is proving to be an effective and powerful technique for modifying genes. The use of the gene editing system in dominantly inherited ataxias like SCA1, where the polyQ expansion in exon 8 of *ATXN1* leads to a gain of toxic protein function, may be the only definitive solution for these kinds of neurodegenerative diseases.

## Supplementary Information

Supplementary Figure S1.

Supplementary Figure S2.

Supplementary Figure S3.

Supplementary Figure S4.

Supplementary Figure S5.

Supplementary Figure S6.

Supplementary Figure S7.

Supplementary Figure S8.

Supplementary Figure S9.

Supplementary Figure S10.

Supplementary Figure S11.

Supplementary Figure S12.

Supplementary Figure S13.

Supplementary Tables.

Supplementary Information 15.

## Data Availability

The data analysed during this study are included in this published article (and its Supplementary Information files) or they are available from the corresponding authors on reasonable request. The NGS datasets generated during the current study are available in the Sequence Read Archive (SRA) repository, the link is: https://dataview.ncbi.nlm.nih.gov/object/PRJNA826320?reviewer=7r0lc5dn171acddl4et51tvchv.

## References

[1] Sullivan R, Yau WY, O'Connor E, Houlden H (2019). Spinocerebellar ataxia: An update. J. Neurol..

[2] Monin ML (2015). Survival and severity in dominant cerebellar ataxias. Ann. Clin. Transl. Neurol..

[3] Lieberman AP, Shakkottai VG, Albin RL (2019). Polyglutamine repeats in neurodegenerative diseases. Annu. Rev. Pathol..

[4] Fan HC (2014). Polyglutamine (PolyQ) diseases: Genetics to treatments. Cell Transpl..

[5] Zoghbi HY, Orr HT (2000). Glutamine repeats and neurodegeneration. Annu. Rev. Neurosci..

[6] Orr HT (2012). Cell biology of spinocerebellar ataxia. J. Cell. Biol..

[7] Schöls L, Bauer P, Schmidt T, Schulte T, Riess O (2004). Autosomal dominant cerebellar ataxias: Clinical features, genetics, and pathogenesis. Lancet Neurol..

[8] Jodice C (1994). Effect of trinucleotide repeat length and parental sex on phenotypic variation in spinocerebellar ataxia I. Am. J. Hum. Gen..

[9] Nagashima Y, Kowa H, Tsuji S, Iwata A (2011). FAT10 protein binds to polyglutamine proteins and modulates their solubility. J. Biol. Chem..

[10] Watase K (2007). Lithium therapy improves neurological function and hippocampal dendritic arborization in a spinocerebellar ataxia type 1 mouse model. PLoS Med..

[11] Menzies FM (2010). Autophagy induction reduces mutant ataxin-3 levels and toxicity in a mouse model of spinocerebellar ataxia type 3. Brain.

[12] Chen ZZ (2015). Trehalose attenuates the gait ataxia and gliosis of spinocerebellar ataxia type 17 mice. Neurochem. Res..

[13] Nascimento-Ferreira I (2011). Overexpression of the autophagic beclin-1 protein clears mutant ataxin-3 and alleviates Machado-Joseph disease. Brain.

[14] Grimm D (2010). Argonaute proteins are key determinants of RNAi efficacy, toxicity, and persistence in the adult mouse liver. J. Clin. Investig..

[15] Friedrich J (2018). Antisense oligonucleotide-mediated ataxin-1 reduction prolongs survival in SCA1 mice and reveals disease-associated transcriptome profiles. JCI Insight..

[16] McLoughlin HS (2018). Oligonucleotide therapy mitigates disease in spinocerebellar ataxia type 3 mice. Ann. Neurol..

[17] KourKouta E (2019). Suppression of mutant protein expression in SCA3 and SCA1 mice using a CAG repeat-targeting antisense oligonucleotide. Mol. Ther. Nucleic Acids..

[18] Chery J (2016). RNA therapeutics: RNAi and antisense mechanisms and clinical applications. Postdoc J..

[19] Jinek M (2012). A programmable dual-RNA-guided DNA endonuclease in adaptive bacterial immunity. Science.

[20] Barrangou R (2007). CRISPR provides acquired resistance against viruses in prokaryotes. Science.

[21] Jinek M (2013). RNA-programmed genome editing in human cells. Elife.

[22] Ouyang S (2018). CRISPR/Cas9 targeted deletion of polyglutamine in spinocerebellar ataxia type 3 derived iPSCs. Stem Cells Dev..

[23] He L (2021). CRISPR/Cas9 mediated gene correction ameliorates abnormal phenotypes in spinocerebellar ataxia type 3 patient-derived induced pluripotent stem cells. Transl. Psychiatry..

[24] Li Y (2015). Excision of expanded GAA repeats alleviates the molecular phenotype of Friedreich’s ataxia. Mol. Ther..

[25] Dabrowska M, Juzwa W, Krzyzosiak WJ, Olejniczak M (2018). Precise excision of the CAG tract from the Huntingtin gene by Cas9 nickases. Front. Neurosci..

[26] Brazelton VA (2015). A quick guide to CRISPR sgRNA design tools. GM Crops Food..

[27] Perez Ortiz JM (2018). Reduction of protein kinase A-mediated phosphorylation of ATXN1-S776 in Purkinje cells delays onset of Ataxia in a SCA1 mouse model. Neurobiol. Dis..

[28] Berry D, Ben MK, Wagner M, Loy A (2011). Barcoded primers used in multiplex amplicon pyrosequencing bias amplification. Appl. Environ. Microbiol..

[29] Herbold CW (2015). A flexible and economical barcoding approach for highly multiplexed amplicon sequencing of diverse target genes. Front. Microbiol..

[30] 16S Metagenomic Sequencing Library Preparation Preparing 16S Ribosomal RNA Gene Amplicons for the Illumina MiSeq System. Preparing 16S Ribosomal RNA Gene Amplicons for the Illumina MiSeq System; https://support.illumina.com/documents/documentation/chemistry_documentation/16s/16s-metagenomic-library-prep-guide-15044223-b.pdf.

[31] Jalili V (2020). The Galaxy platform for accessible, reproducible and collaborative biomedical analyses: 2020 update. Nucleic Acids Res..

[32] Robinson JT (2011). Integrative genomics viewer. Nat. Biotechnol..

[33] Maeder ML, Gersbach CA (2016). Genome-editing technologies for gene and cell therapy. Mol. Ther..

[34] Lino CA, Harper JC, Carney JP, Timlin JA (2018). Delivering CRISPR: A review of the challenges and approaches. Drug Deliv..

[35] DeWitt MA, Corn JE, Carroll D (2017). Genome editing via delivery of Cas9 ribonucleoprotein. Methods.

[36] Jacobi AM (2017). Simplified CRISPR tools for efficient genome editing and streamlined protocols for their delivery into mammalian cells and mouse zygotes. Methods.

[37] Becerra NY, Arenas CM, Patiño MI, Delgado JP, Muskus CE, Restrepo LM (2018). Polyplex system versus nucleofection for human skin cell transfection and effect of internal ribosome entry site sequence. Tissue Eng. Part C Methods..

[38] Kucharski M, Mrowiec P, Ocłon E (2021). Current standards and pitfalls associated with the transfection of primary fibroblast cells. Biotechnol. Prog..

[39] Li H, Durbin R (2009). Fast and accurate short read alignment with Burrows–Wheeler transform. Bioinformatics.

[40] Garrison, E. & Marth, G. Haplotype-based variant detection from short-read sequencing. *arViv*. 1207.3907 [q-bio.GN] (2012).

[41] Bao XR (2021). Tools for experimental and computational analyses of off-target editing by programmable nucleases. Nat. Protoc..

[42] Brusco A (2004). Molecular genetics of hereditary spinocerebellar ataxia: mutation analysis of spinocerebellar ataxia genes and CAG/CTG repeat expansion detection in 225 Italian families. Arch. Neurol..

[43] Yang S (2017). CRISPR/Cas9-mediated gene editing ameliorates neurotoxicity in mouse model of Huntington's disease. J. Clin. Investig..

[44] Yu X (2016). Improved delivery of Cas9 protein/gRNA complexes using lipofectamine CRISPRMAX. Biotechnol. Lett..

[45] Abbasi S (2021). Co-encapsulation of Cas9 mRNA and guide RNA in polyplex micelles enables genome editing in mouse brain. J. Control Release..

[46] Pardridge WM (2020). Blood-brain barrier and delivery of protein and gene therapeutics to brain. Front. Aging Neurosci..

[47] Zhang S, Shen J, Li D, Cheng Y (2021). Strategies in the delivery of Cas9 ribonucleoprotein for CRISPR/Cas9 genome editing. Theranostics..

[48] Rousseaux MW (2018). ATXN1-CIC complex is the primary driver of cerebellar pathology in spinocerebellar ataxia type 1 through a gain-of-function mechanism. Neuron.

[49] Suh J (2019). Loss of ataxin-1 potentiates Alzheimer's pathogenesis by elevating cerebral BACE1 transcription. Cell.

[50] Asher M, Johnson A, Zecevic B, Pease D, Cvetanovic M (2016). Ataxin-1 regulates proliferation of hippocampal neural precursors. Neuroscience.

[51] O’Callaghan B (2020). Antisense oligonucleotide therapeutic approach for suppression of ataxin-1 expression: A safety assessment. Mol. Ther. Nucleic Acids..

[52] Kadam US, Shelake RM, Chavhan RL, Suprasanna P (2018). Concerns regarding 'off-target' activity of genome editing endonucleases. Plant Physiol. Biochem..

[53] Fu Y (2013). High-frequency off-target mutagenesis induced by CRISPR-Cas nucleases in human cells. Nat. Biotechnol..

[54] Hsu PD (2013). DNA targeting specificity of RNA-guided Cas9 nucleases. Nat. Biotechnol..

[55] Lin Y (2014). CRISPR/Cas9 systems have off-target activity with insertions or deletions between target DNA and guide RNA sequences. Nucleic Acids Res..

