# Supplementary material for: Suppressing gain-of-function proteins via CRISPR/Cas9 system in SCA1 cells
Source: Sci Rep. 2022 Nov 24;12:20285. doi: 10.1038/s41598-022-24299-y (PMC9700751; doi:10.1038/s41598-022-24299-y)
Supplement: Supplementary file 1 — Supplementary Figure S1. [file 41598_2022_24299_MOESM1_ESM.pdf]

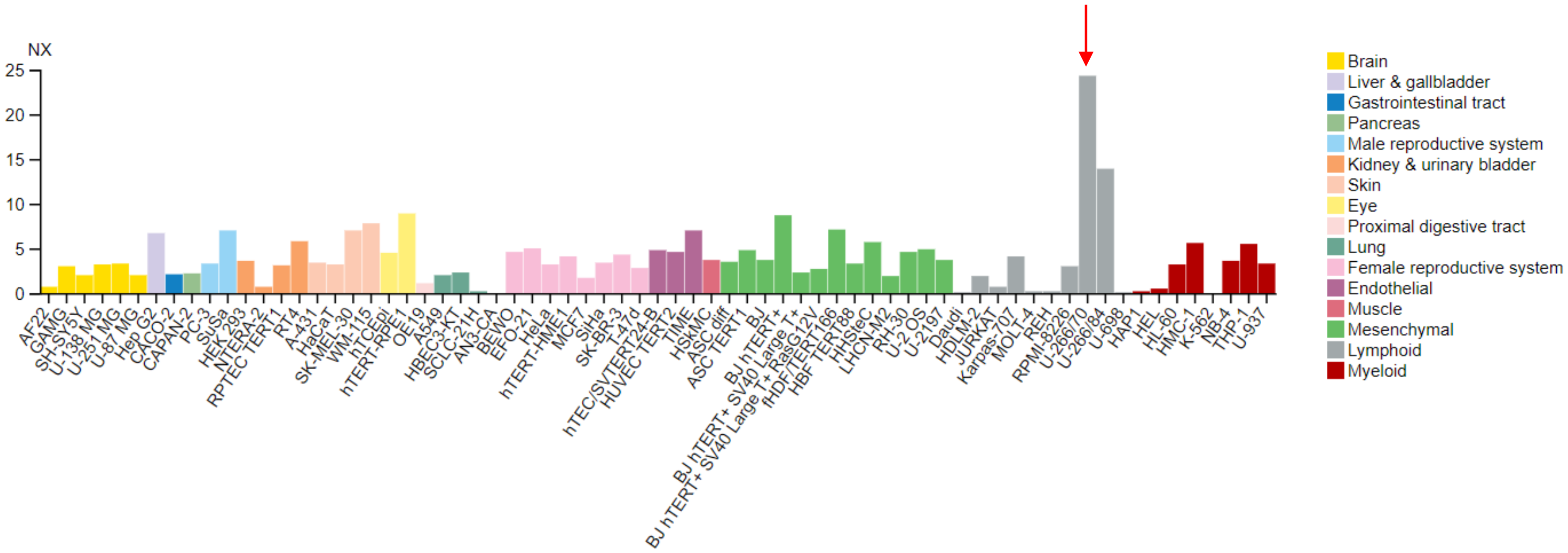

Figure S1. ATXN1 expression in cell lines. Evaluation of the expression of the ATXN1 gene in the most common cell lines, published on The Human Protein Atlas website (https://www.proteinatlas.org/ENSG00000124788-ATXN1/celltype). The arrow points to the U266 cells used for some preliminary experiments.
