# Supplementary material for: Suppressing gain-of-function proteins via CRISPR/Cas9 system in SCA1 cells
Source: Sci Rep. 2022 Nov 24;12:20285. doi: 10.1038/s41598-022-24299-y (PMC9700751; doi:10.1038/s41598-022-24299-y)
Supplement: Supplementary file 2 — Supplementary Figure S2. [file 41598_2022_24299_MOESM2_ESM.pdf]

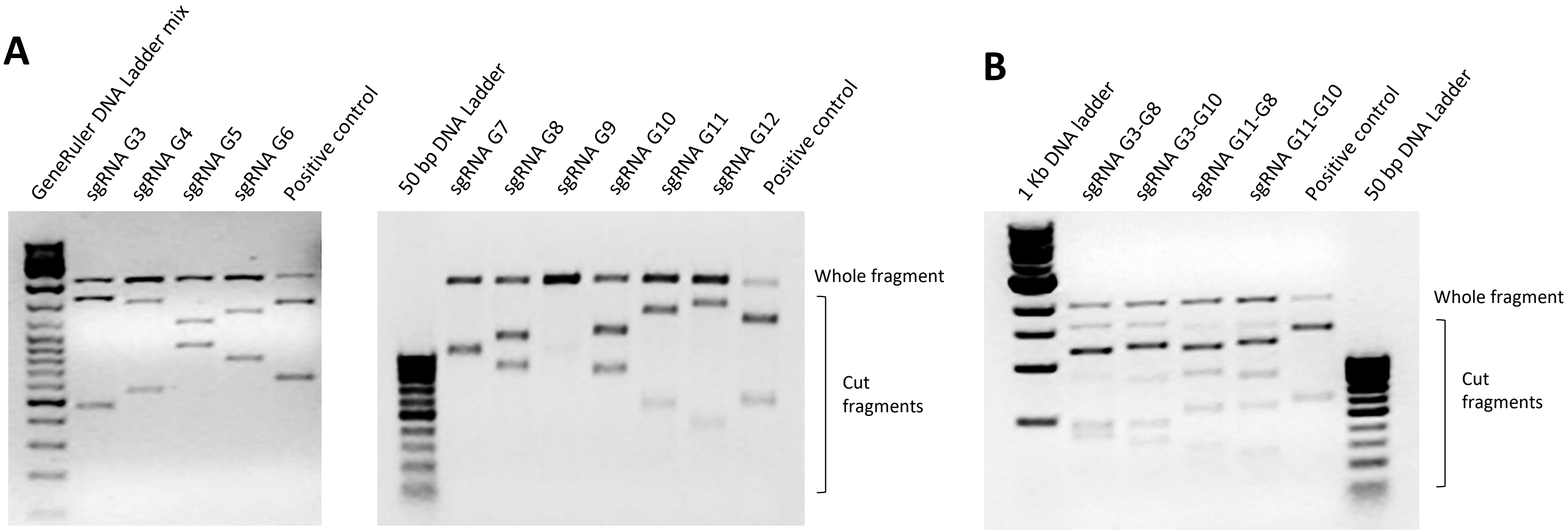

Figure S2. *In vitro* screening of sgRNA for ATXN1 gene. A 2000-bp target fragment of ATXN1 gene was amplified, and the sgRNAs designed to cleave the amplified sequence were in vitro transcribed (using the Guide-it Complete sgRNA Screening System). For each reaction, the target fragment, a sgRNA, and recombinant Cas9 enzyme (also included in kit) were combined in an in vitro cleavage reaction according to the protocol. Densitometer analysis of the agarose gel indicated the target cleavage efficiency. **A,B.** Electrophoretic stroke of the fragments obtained by cutting with one (**A**) or a pair (**B**) of sgRNAs of the target fragment. The positive control consists of a 2000 bp DNA fragment cut from a control Cas9/sgRNA complex, both supplied by the kit. This control was designed to achieve high efficiency DNA double helix cleavage. For this reason, it was used as a comparison for the relative quantification of the cleavage efficiency of the designed sgRNAs. DNA ladder used: 1 Kb plus DNA ladder (100 bp, 200 bp, 300 bp, 400 bp, 500 bp, 600 bp, 700 bp, 800 bp, 900 bp, 1000 bp, 1200 bp, 1500 bp, 2000 bp, 2500 bp, 3000 bp, 3500 bp, 4000 bp, 5000 bp, 6000 bp, 8000 bp, 10000 bp); 1 Kb DNA Ladder (250 bp, 500 bp, 750 bp, 1000 bp, 1500 bp, 2000 bp, 2500 bp, 3000 bp, 4000 bp, 5000 bp, 6000 bp, 8000 bp, 10000 bp); GeneRuler 50 bp DNA Ladder (50 bp, 100 bp, 150 bp, 200 bp, 250 bp, 300 bp, 400 bp, 500 bp, 600 bp, 700 bp, 800 bp, 900 bp, 1000 bp).
