# Supplementary material for: Suppressing gain-of-function proteins via CRISPR/Cas9 system in SCA1 cells
Source: Sci Rep. 2022 Nov 24;12:20285. doi: 10.1038/s41598-022-24299-y (PMC9700751; doi:10.1038/s41598-022-24299-y)
Supplement: Supplementary file 3 — Supplementary Figure S3. [file 41598_2022_24299_MOESM3_ESM.pdf]

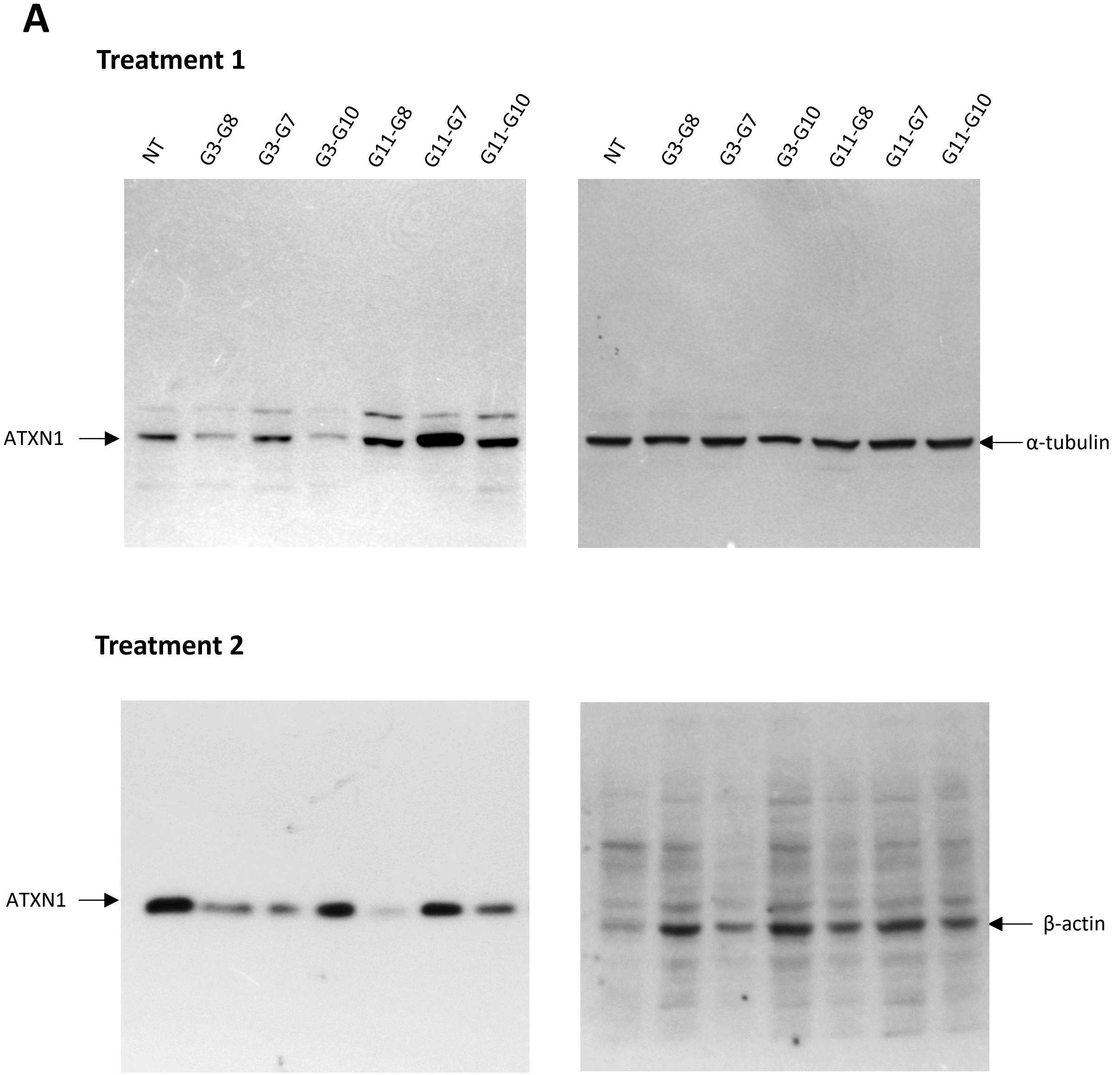

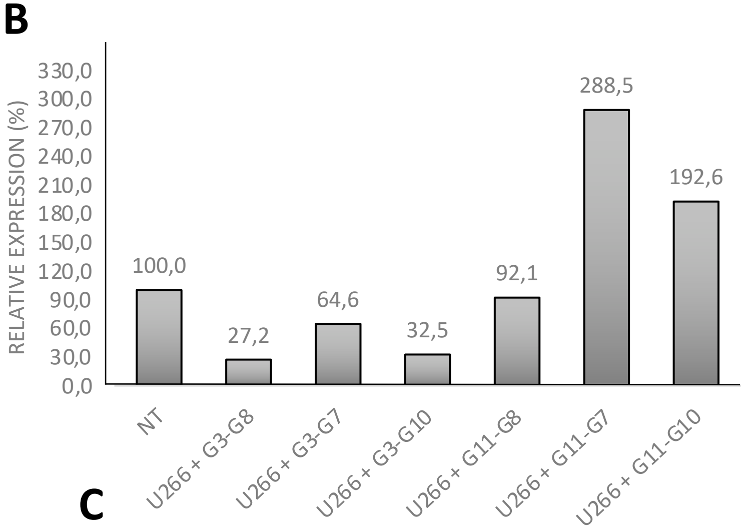

C

Samples	Vol. adj ATXN1	Vol. adj housekeeping
**TREATMENT 1**		
NT	7.751.453	10.840.060
sgRNA G3-G8	2.661.296	9.967.860
sgRNA G3-G7	8.213.034	11.281.834
sgRNA G3-G10	2.311.733	8.636.862
sgRNA G11-G8	13.081.145	10.268.825
sgRNA G11-G7	42.930.017	11.045.067
sgRNA G11-G10	26.797.772	10.712.268
**TREATMENT 2**		
NT	2.425.021	1.439.406
sgRNA G3-G8	1.129.013	3.920.445
sgRNA G3-G7	876.822	1.898.090
sgRNA G3-G10	1.846.898	3.966.278
sgRNA G11-G8	213.987	2.096.389
sgRNA G11-G7	2.002.016	3.555.253
sgRNA G11-G10	1.266.466	2.120.582

Figure S3. Screening of sgRNA pairs in an in vitro cellular model. **A,B**, ATXN1 expression in U266 cell. **A**, Cells were treated using sgRNAs pairs complexed with Cas9 endonucleases and the ATXN1 expression was determined by Western Blotting. **B**, ATXN1 abundances were expressed relative to α-tubulin and β-actin, determined by densitometry. **C**, Raw data obtained by densitometry of Western Blotting bands, using the Image Lab 6.0 software. Adjusted Volume means the background-adjusted volume, which is the sum of all the intensities within the band boundaries. Values are mean from two independent experiments. NT: untreated sample; KO: treated sample; Cas9 C-: sample treated with scramble sgRNA.
