# Supplementary material for: Suppressing gain-of-function proteins via CRISPR/Cas9 system in SCA1 cells
Source: Sci Rep. 2022 Nov 24;12:20285. doi: 10.1038/s41598-022-24299-y (PMC9700751; doi:10.1038/s41598-022-24299-y)
Supplement: Supplementary file 4 — Supplementary Figure S4. [file 41598_2022_24299_MOESM4_ESM.pdf]

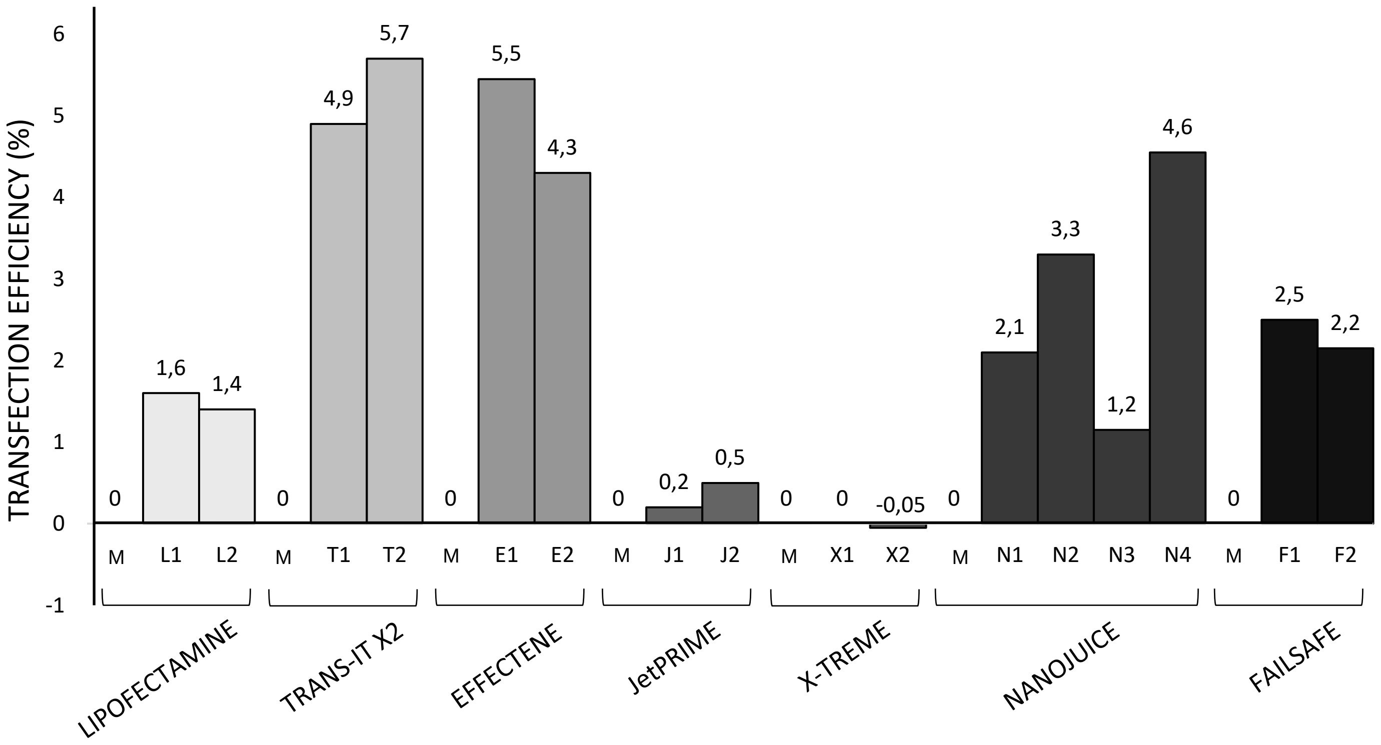

Figure S4. Evaluation of lipofection efficiency in normal and SCA1 fibroblasts. pcDNA3 (M: Mock) or pcDNA3-EGFP were transfected into normal fibroblasts using seven different liposomal agents and the transfection efficiency was determined as percentage of fluorescent cells, assessed after 24 hours by FACS analysis. Values are mean from two independent experiments.
