# Supplementary material for: Suppressing gain-of-function proteins via CRISPR/Cas9 system in SCA1 cells
Source: Sci Rep. 2022 Nov 24;12:20285. doi: 10.1038/s41598-022-24299-y (PMC9700751; doi:10.1038/s41598-022-24299-y)
Supplement: Supplementary file 5 — Supplementary Figure S5. [file 41598_2022_24299_MOESM5_ESM.pdf]

A

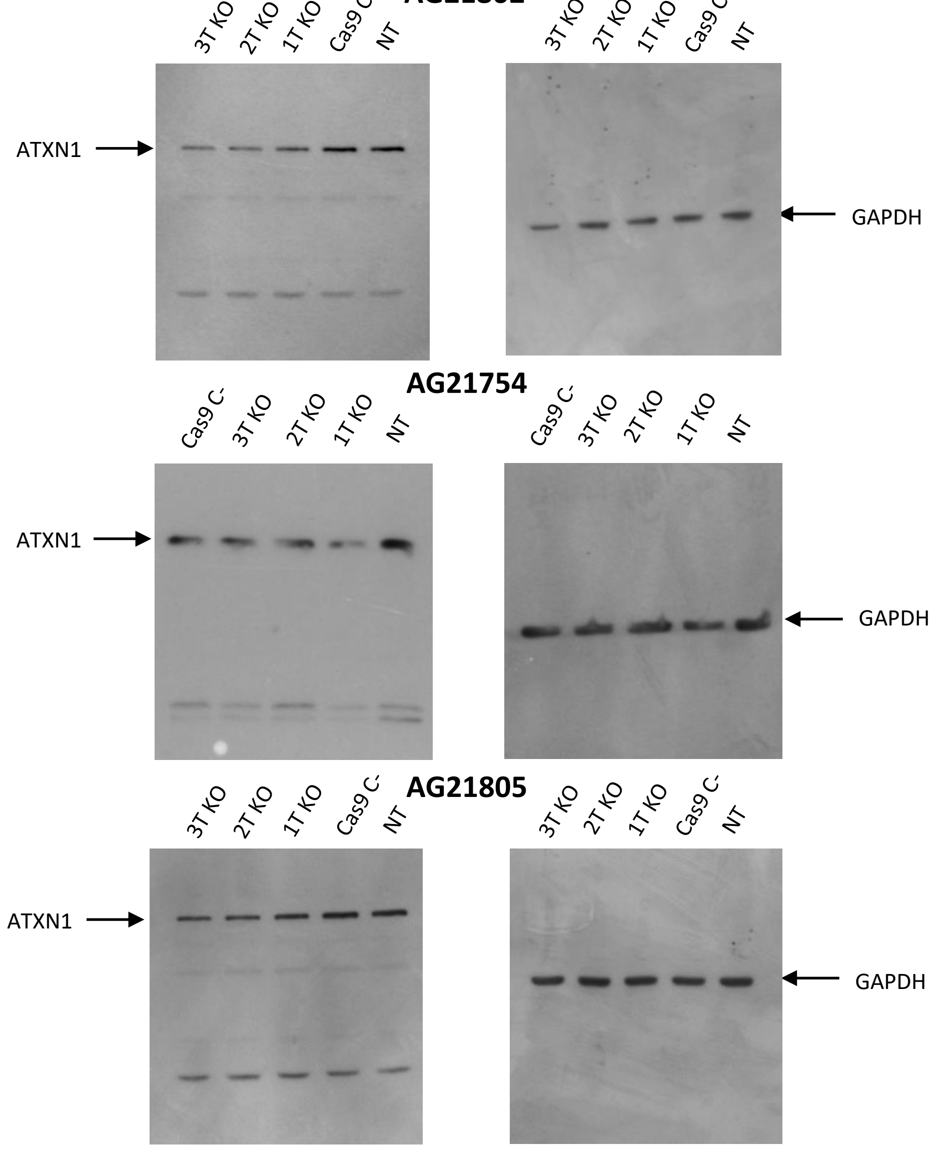

B

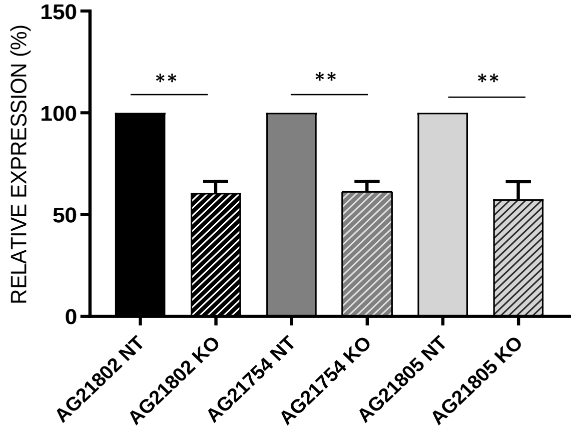

C

Samples	Vol. adj ATXN1	Vol. adj GAPDH
**AG21802**		
NT	6.543.310	2.143.094
Cas9 C-	5.819.277	1.895.502
1T KO	3.770.326	1.823.578
2T KO	2.965.651	1.958.083
3T KO	2.480.605	1.254.446
**AG21754**		
NT	2.921.005	2.230.999
1T KO	1.046.812	1.558.187
2T KO	1.895.241	2.233.809
3T KO	1.680.929	1.895.946
Cas9 C-	1.983.412	1.919.064
**AG21805**		
NT	5.469.928	2.571.886
Cas9 C-	5.716.209	2.365.082
1T KO	3.263.316	2.217.648
2T KO	2.165.656	2.494.861
3T KO	2.031.849	1.522.009

Figure S5. Effects of CRISPR/Cas9 system on ATXN1 expression in normal fibroblasts. **A,B**, ATXN1 expression in SCA1 fibroblast. **A**, Fibroblasts from three normal subjects were treated using sgRNAs G3 and G8 complexed with Cas9 endonucleases and the ATXN1 expression was determined by Western Blotting. **B**, ATXN1 abundances were expressed relative to GAPDH, determined by densitometry. **C**, Raw data obtained by densitometry of Western Blotting bands, using the Image Lab 6.0 software. Adjusted Volume means the background-adjusted volume, which is the sum of all the intensities within the band boundaries. Values are mean ± s.e.m. from three independent experiments. The statistical test used was unpaired t test with two-tailed P value and alpha level P<0.05. **p < 0,005. NT: untreated sample; KO: treated sample; Cas9 C-: sample treated with scramble sgRNA.
