# Supplementary material for: Suppressing gain-of-function proteins via CRISPR/Cas9 system in SCA1 cells
Source: Sci Rep. 2022 Nov 24;12:20285. doi: 10.1038/s41598-022-24299-y (PMC9700751; doi:10.1038/s41598-022-24299-y)
Supplement: Supplementary file 6 — Supplementary Figure S6. [file 41598_2022_24299_MOESM6_ESM.pdf]

A

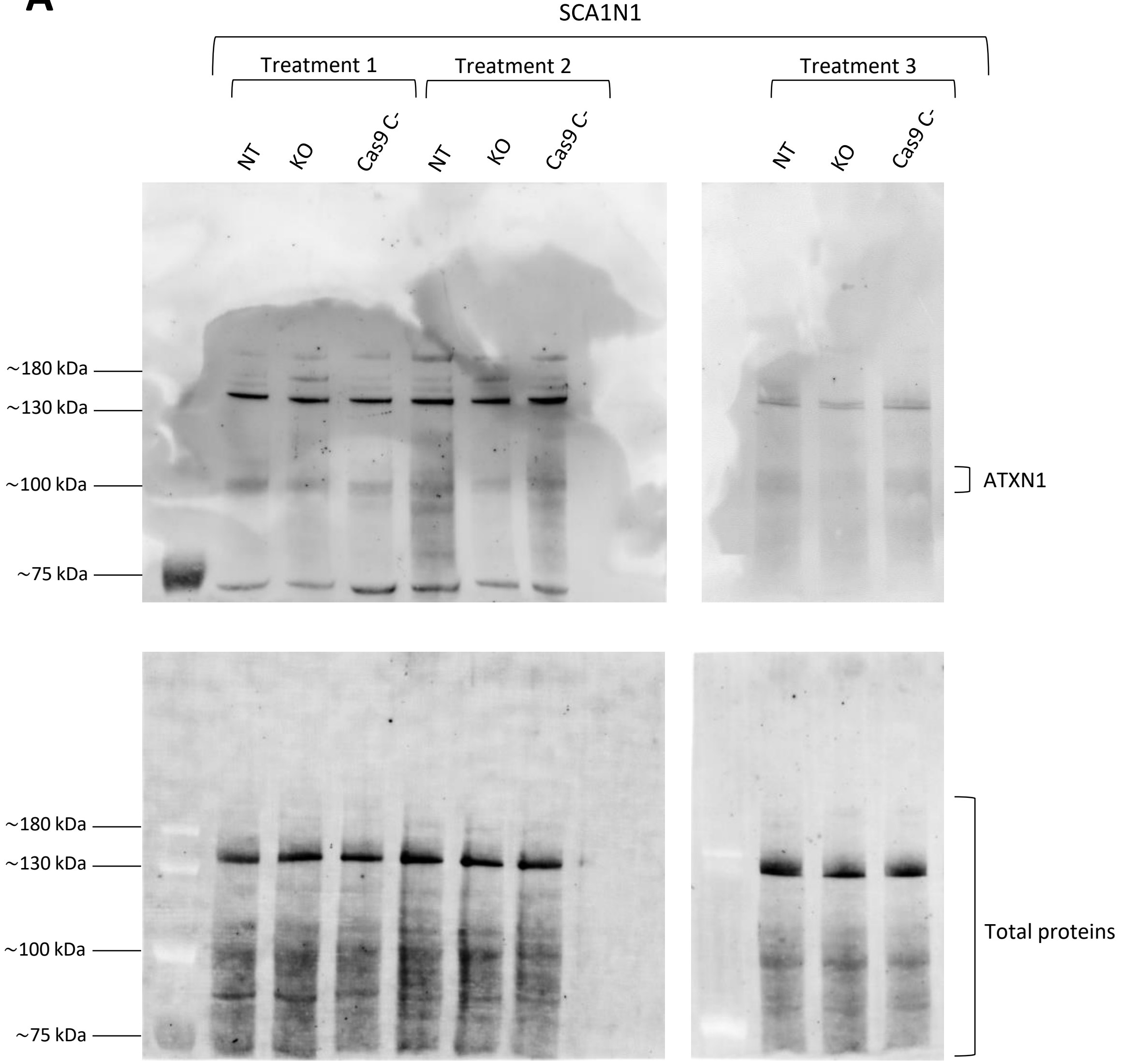

B

Samples	Adj. Vol. ATXN1	Adj. Vol. Total proteins
**Treatment 1**		
SCA1N1 NT	30.939.058	427.074.249
SCA1N1 KO	10.711.207	437.725.372
SCA1N1 Cas9 C-	22.284.091	461.746.163
**Treatment 2**		
SCA1N1NT	32.439.680	552.354.179
SCA1N1 KO	15.001.993	451.201.779
SCA1N1 Cas9 C-	30.422.882	413.321.218
**Treatment 3**		
SCA1N1 NT	49.413.283	561.211.693
SCA1N1 KO	22.932.449	589.239.936
SCA1N1 Cas9 C-	40.927.236	566.432.745

Figure S6. Effects of CRISPR/Cas9 system on ATXN1 expression in SCA1 fibroblasts.

C

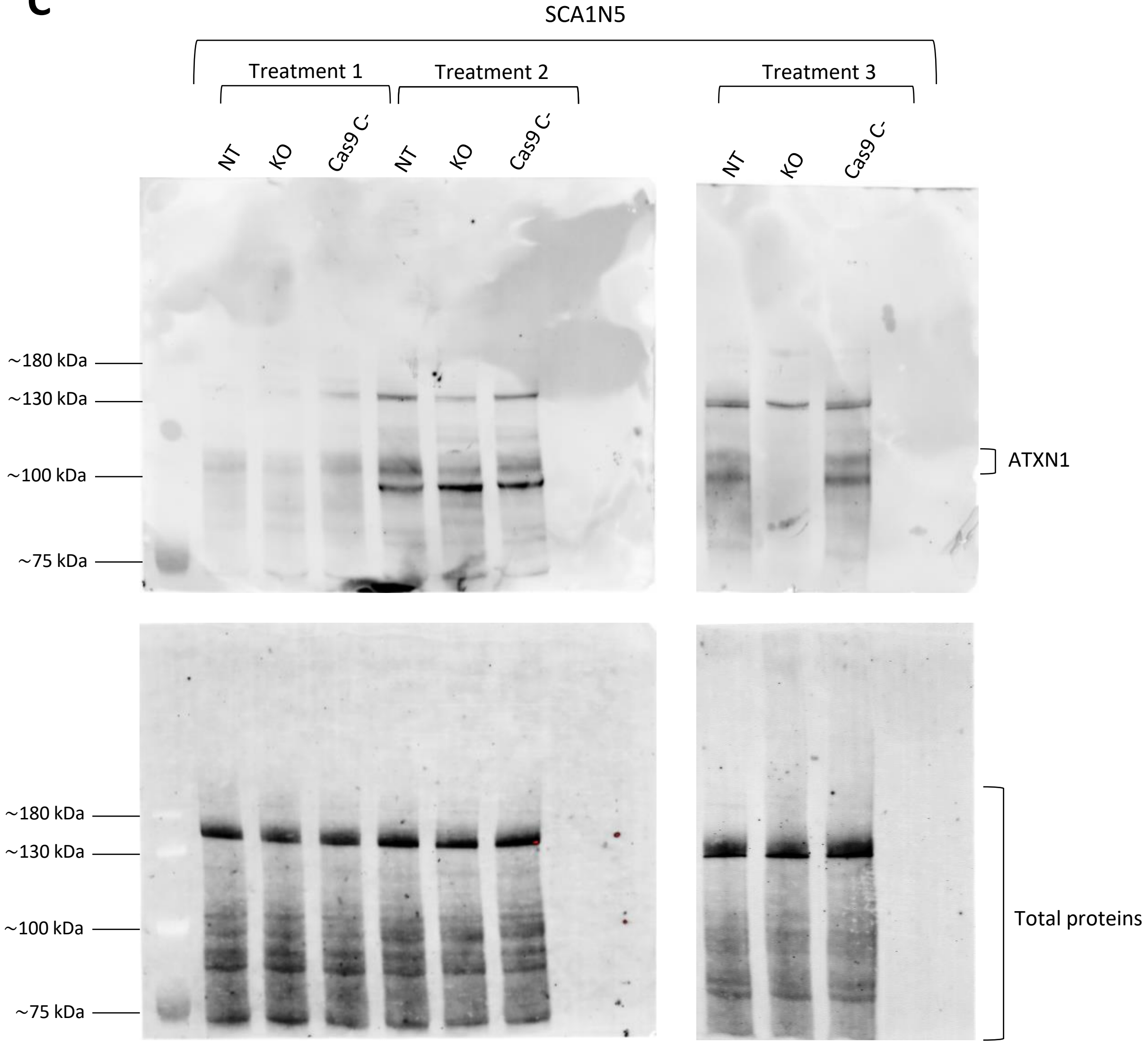

D

Samples	Adj. Vol. ATXN1	Adj. Vol. Total proteins
**Treatment 1**		
SCA1N5 NT	25.097.450	642.613.237
SCA1N5 KO	19.014.508	759.380.948
SCA1N5 Cas9 C-	34.995.658	721.852.236
**Treatment 2**		
SCA1N5 NT	96.464.636	710.474.680
SCA1N5 KO	49.155.769	641.455.285
SCA1N5 Cas9 C-	62.228.553	594.597.890
**Treatment 3**		
SCA1N5 NT	114.354.677	703.475.170
SCA1N5 KO	15.838.076	768.194.824
SCA1N5 Cas9 C-	110.069.479	486.957.354

Figure S6. Effects of CRISPR/Cas9 system on ATXN1 expression in SCA1 fibroblasts.

E

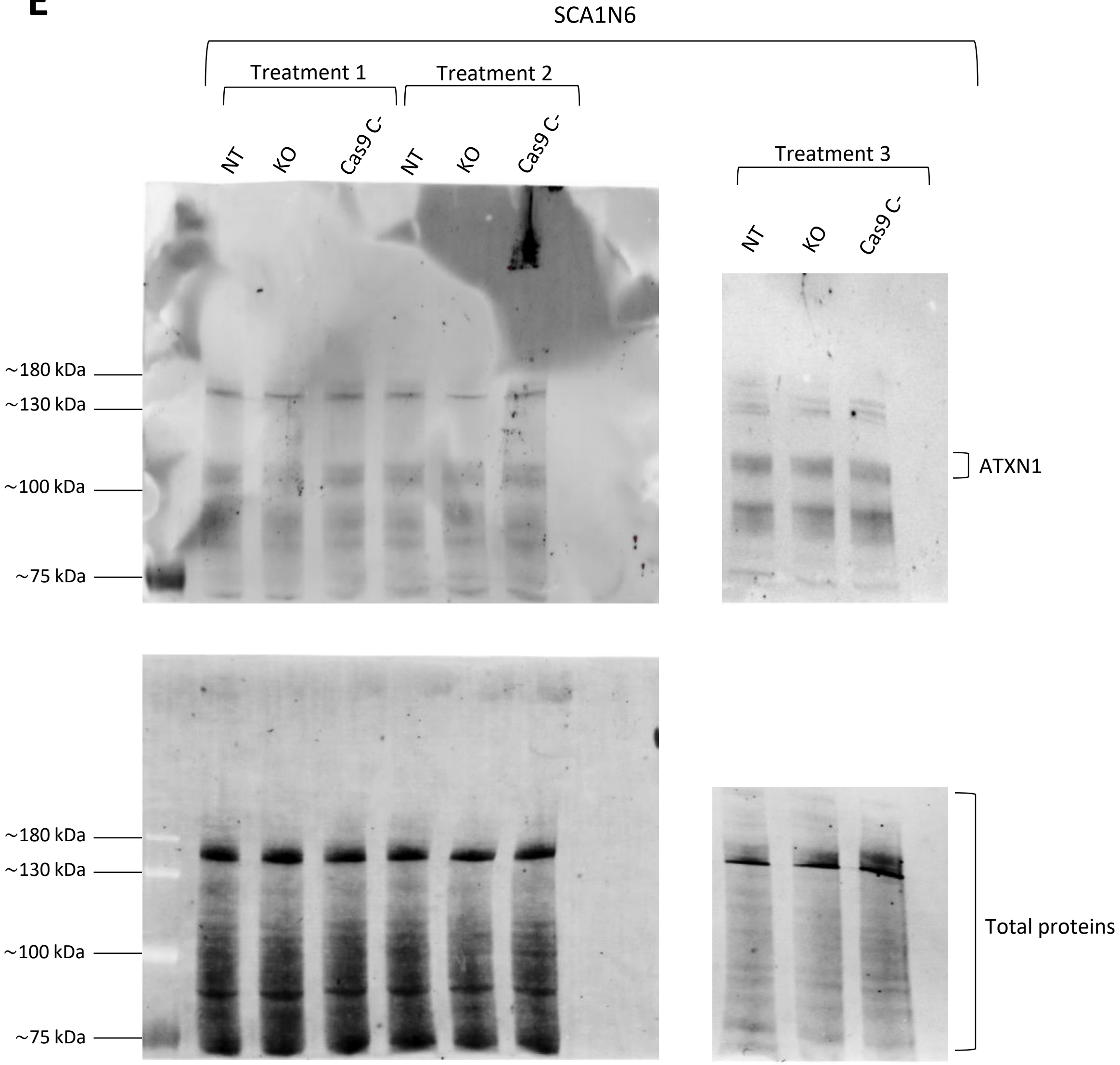

F

Samples	Adj. Vol. ATXN1	Adj. Vol. Total proteins
**Treatment 1**		
SCA1N6 NT	28.592.943	113.671.538
SCA1N6 KO	16.335.656	107.977.561
SCA1N6 Cas9 C-	21.458.010	100.176.912
**Treatment 2**		
SCA1N6 NT	19.890.546	113.877.997
SCA1N6 KO	11.762.817	89.182.109
SCA1N6 Cas9 C-	13.788.550	94.548.584
**Treatment 3**		
SCA1N6 NT	4.219.739	271.310.565
SCA1N6 KO	3.724.752	278.979.304
SCA1N6 Cas9 C-	3.493.452	249.243.291

Figure S6. Effects of CRISPR/Cas9 system on ATXN1 expression in SCA1 fibroblasts.

G

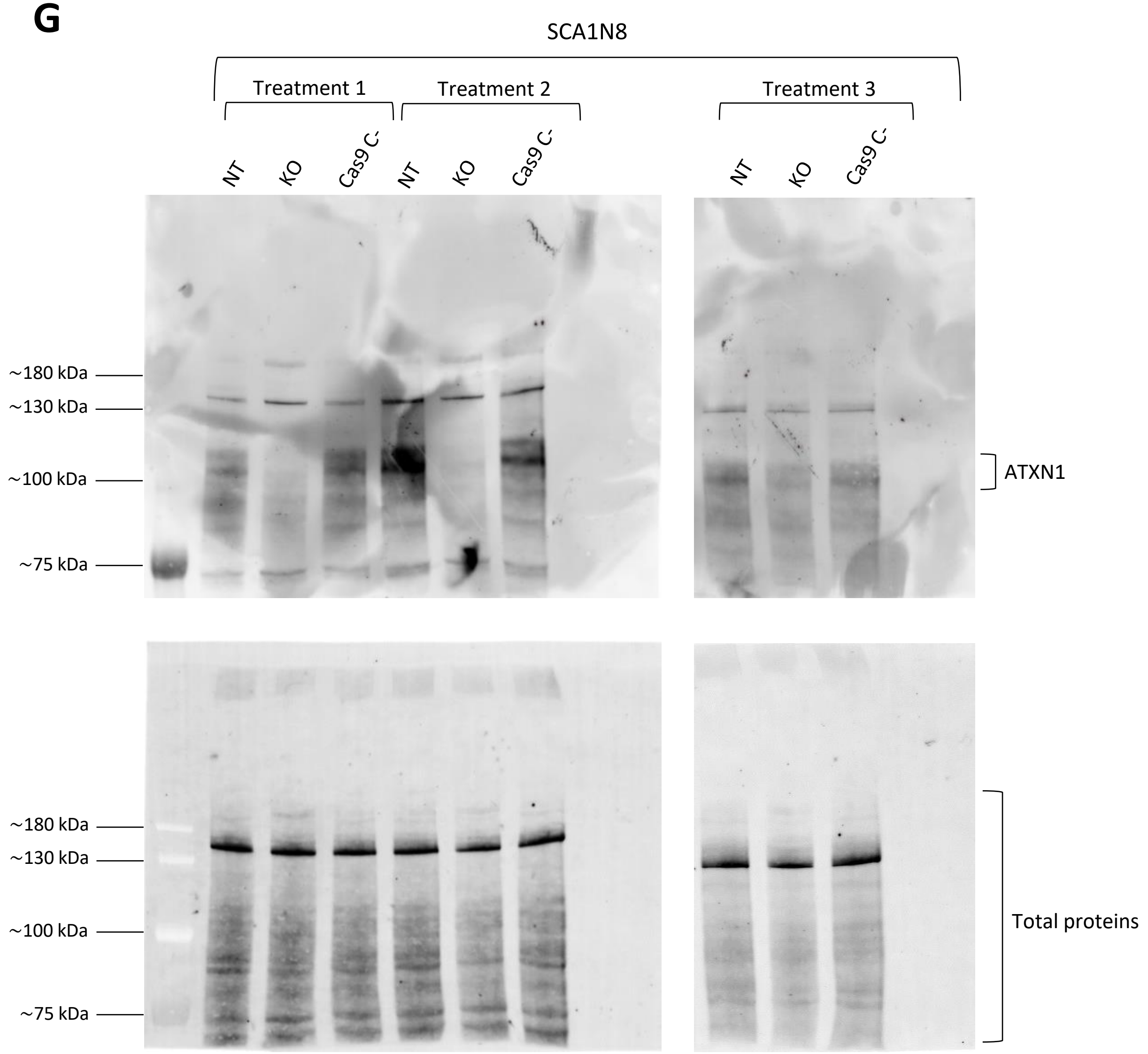

H

Samples	Adj. Vol. ATXN1	Adj. Vol. Total proteins
**Treatment 1**		
SCA1N8 NT	44.512.034	621.744.601
SCA1N8 KO	3.498.857	580.868.835
SCA1N8 Cas9 C-	51.554.483	634.720.980
**Treatment 2**		
SCA1N8 NT	75.227.152	659.367.492
SCA1N8 KO	4.800.378	505.317.194
SCA1N8 Cas9 C-	74.563.066	542.981.685
**Treatment 3**		
SCA1N8 NT	186.829.233	932.791.223
SCA1N8 KO	102.353.650	868.580.287
SCA1N8 Cas9 C-	154.779.964	746.004.100

Figure S6. Effects of CRISPR/Cas9 system on ATXN1 expression in SCA1 fibroblasts.

I

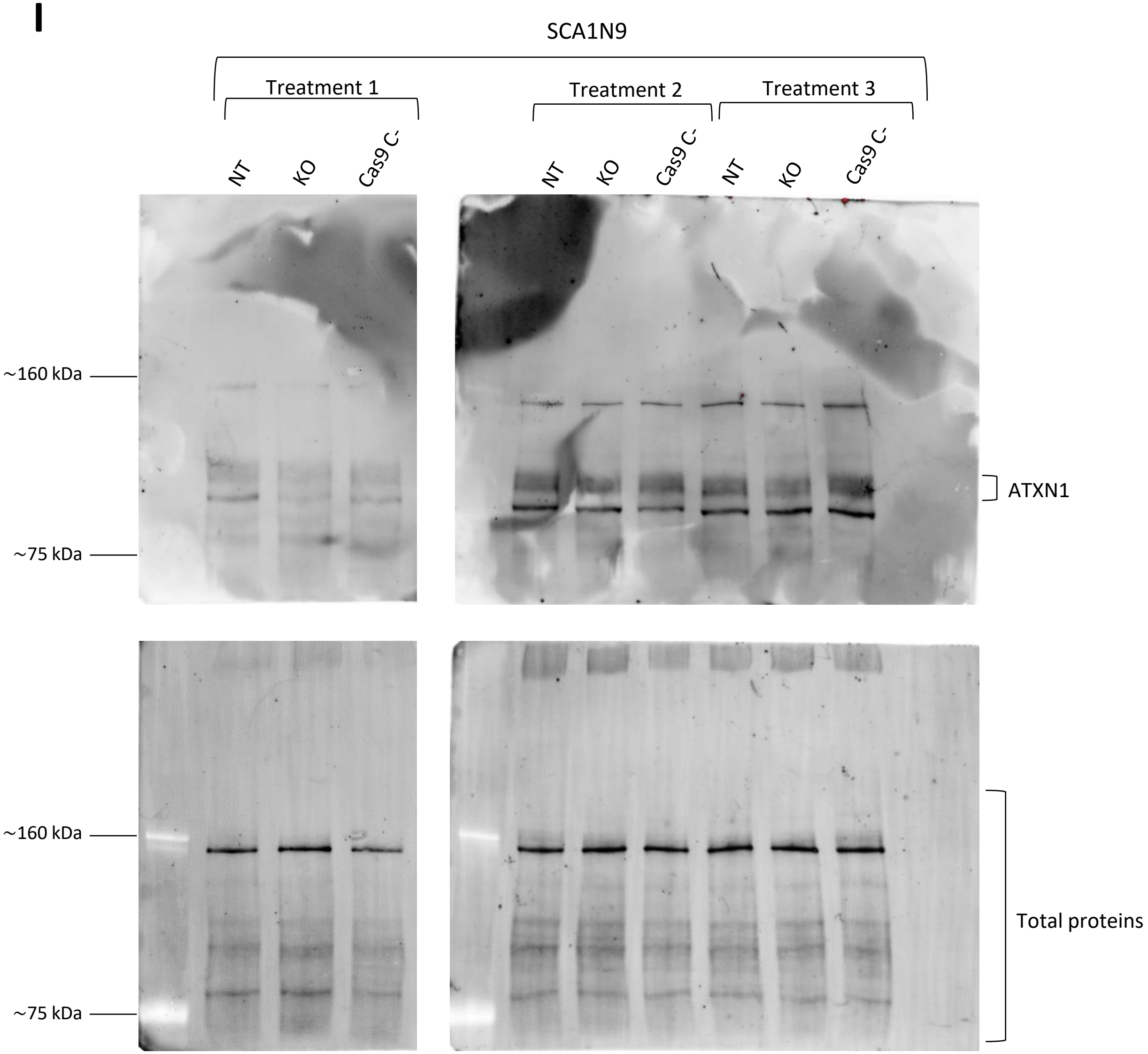

L

Samples	Adj. Vol. ATXN1	Adj. Vol. Total proteins
**Treatment 1**		
SCA1N9 NT	42.379.870	319.128.450
SCA1N9 KO	24.180.291	325.982.139
SCA1N9 Cas9 C-	35.089.818	268.623.993
**Treatment 2**		
SCA1N9 NT	25.952.230	259.756.846
SCA1N9 KO	24.306.072	316.936.250
SCA1N9 Cas9 C-	28.324.149	280.673.046
**Treatment 3**		
SCA1N9 NT	53.915.530	226.324.410
SCA1N9 KO	39.448.398	297.253.724
SCA1N9 Cas9 C-	56.246.234	281.697.290

Figure S6. Effects of CRISPR/Cas9 system on ATXN1 expression in SCA1 fibroblasts.

M

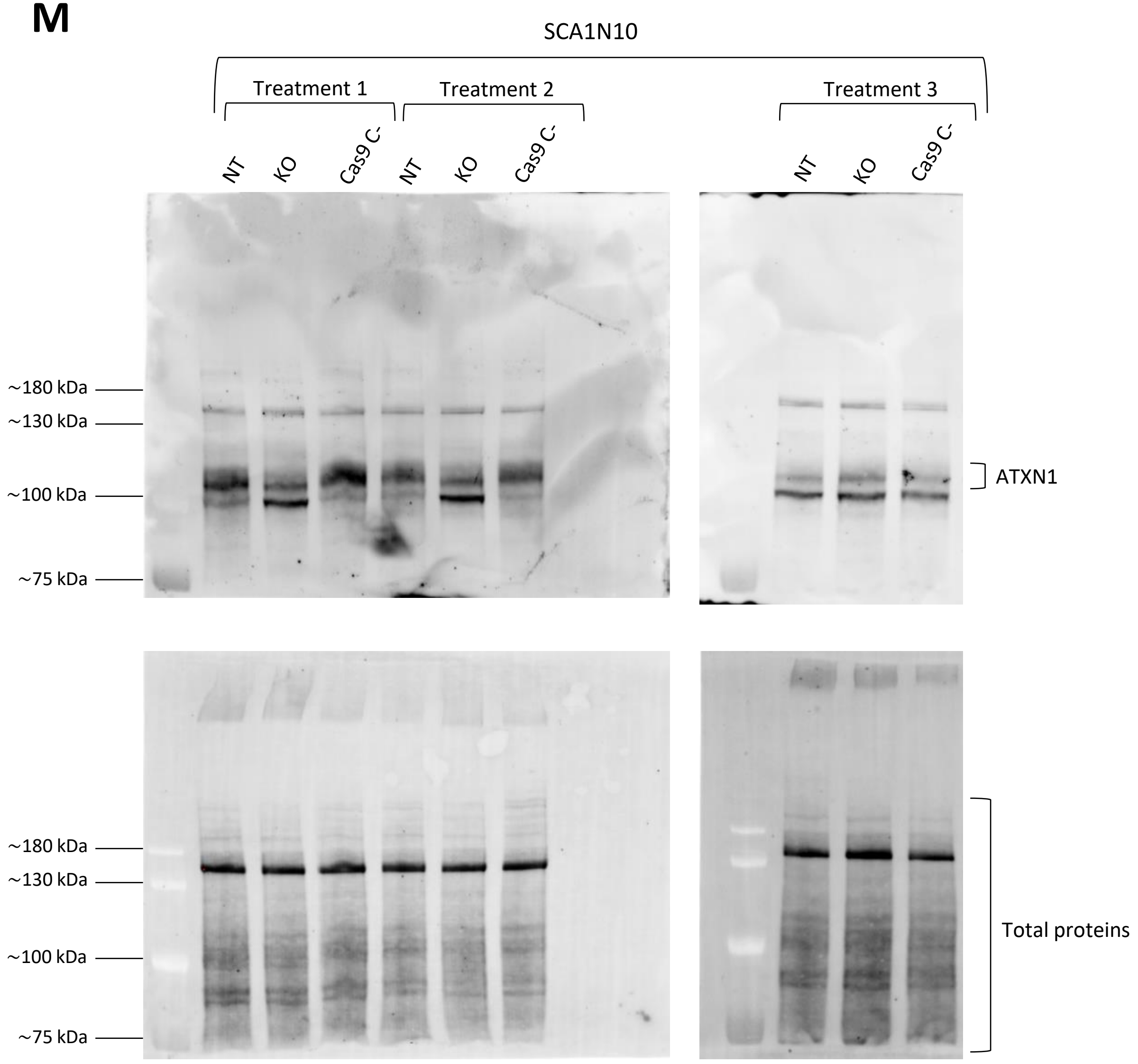

N

Samples	Adj. Vol. ATXN1	Adj. Vol. Total proteins
**Treatment 1**		
SCA1N10 NT	89.388.367	839.941.885
SCA1N10 KO	51.756.500	888.419.004
SCA1N10 Cas9 C-	101.744.886	858.863.057
**Treatment 2**		
SCA1N10 NT	82.829.113	772.893.905
SCA1N10 KO	35.038.367	813.343.880
SCA1N10 Cas9 C-	84.879.492	839.683.443
**Treatment 3**		
SCA1N10 NT	23.921.264	680.769.039
SCA1N10 KO	29.273.385	1.062.595.389
SCA1N10 Cas9 C-	23.313.406	744.241.794

Figure S6. Effects of CRISPR/Cas9 system on ATXN1 expression in SCA1 fibroblasts.

O

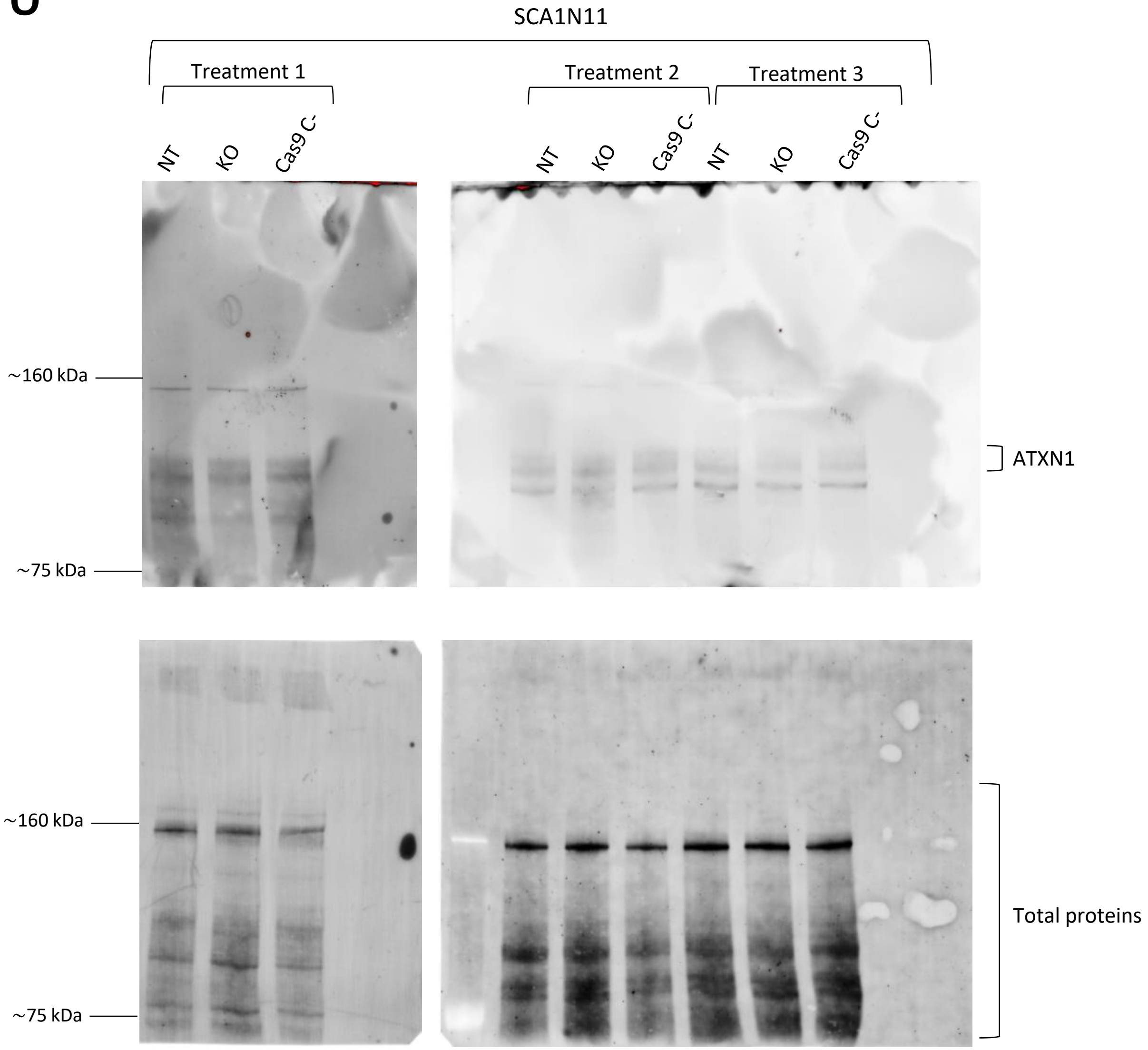

P

Samples	Adj. Vol. ATXN1	Adj. Vol. Total proteins
**Treatment 1**		
SCA1N11 NT	38.881.358	426.528.261
SCA1N11 KO	26.326.442	440.670.192
SCA1N11 Cas9 C-	39.644.005	310.536.353
**Treatment 2**		
SCA1N11 NT	31.214.676	707.167.925
SCA1N11 KO	34.362.108	933.825.406
SCA1N11 Cas9 C-	34.354.669	679.220.521
**Treatment 3**		
SCA1N11 NT	27.347.431	704.024.157
SCA1N11 KO	18.282.827	781.457.184
SCA1N11 Cas9 C-	19.539.687	786.333.340

Figure S6. Effects of CRISPR/Cas9 system on ATXN1 expression in SCA1 fibroblasts.

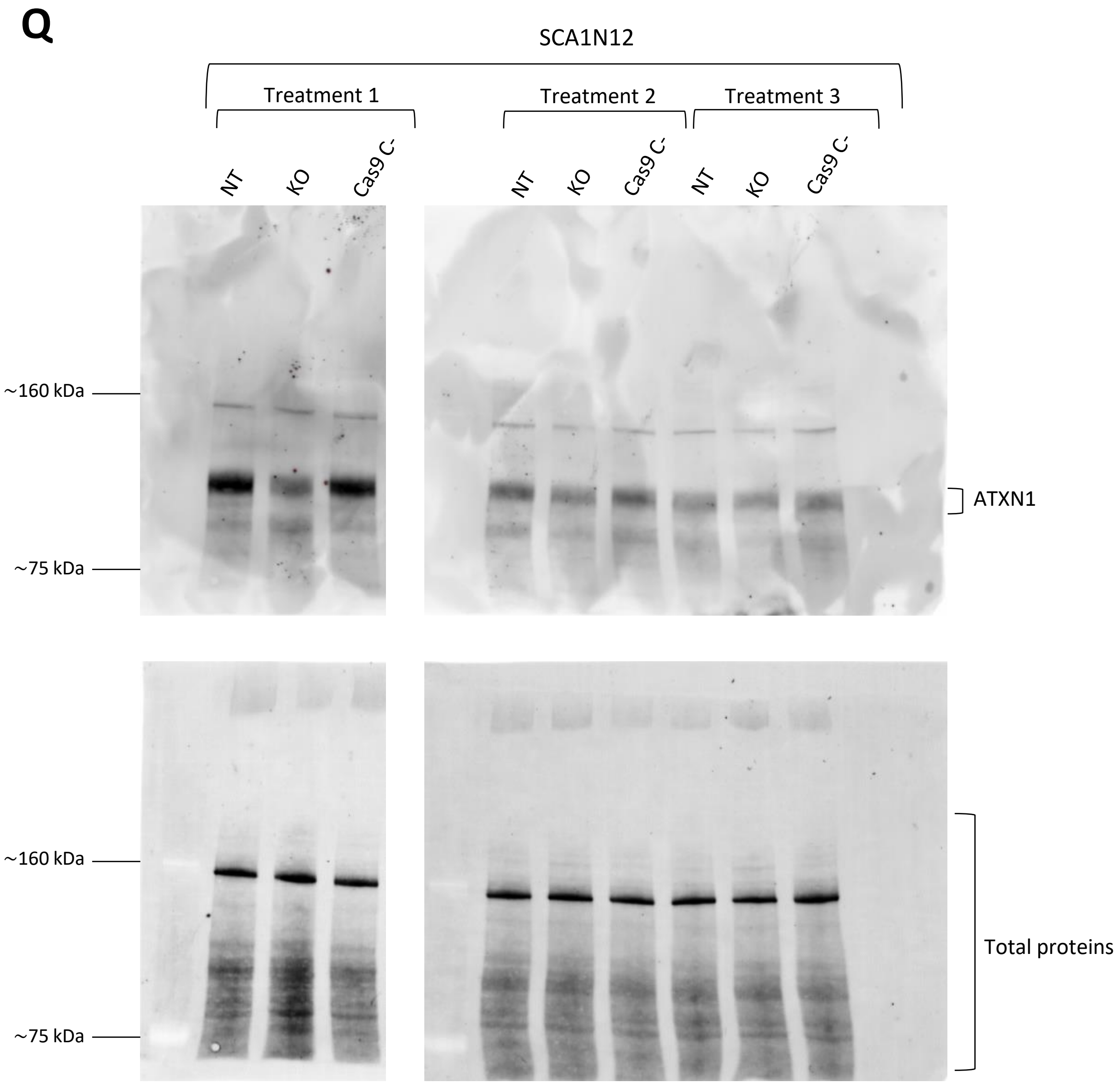

R

Samples	Adj. Vol. ATXN1	Adj. Vol. Total proteins
**Treatment 1**		
SCA1N12 NT	318.814.664	378.007.481
SCA1N12 KO	149.779.609	642.514.633
SCA1N12 Cas9 C-	279.988.162	291.277.840
**Treatment 2**		
SCA1N12 NT	257.715.910	443.672.852
SCA1N12 KO	176.463.500	446.981.180
SCA1N12 Cas9 C-	244.046.882	476.421.680
**Treatment 3**		
SCA1N12 NT	168.601.004	585.731.248
SCA1N12 KO	121.618.090	489.413.763
SCA1N12 Cas9 C-	156.339.773	488.298.062

Figure S6. Effects of CRISPR/Cas9 system on ATXN1 expression in SCA1 fibroblasts.

S

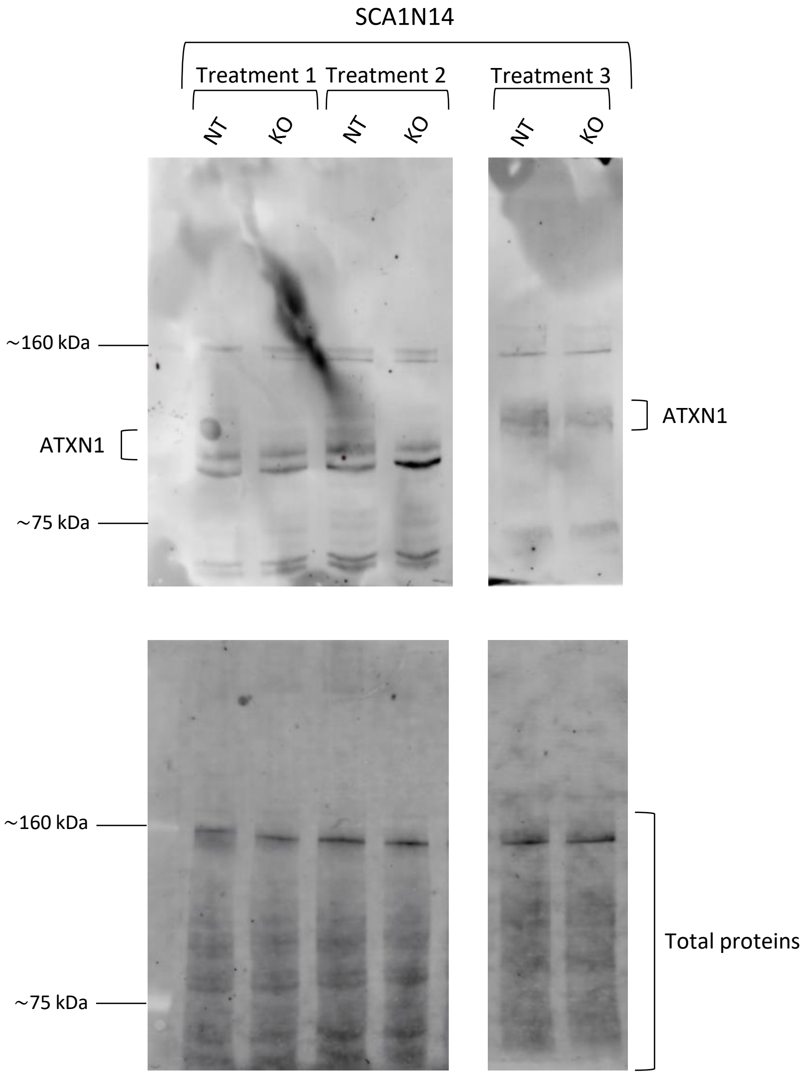

T

Samples	Adj. Vol. ATXN1	Adj. Vol. Total proteins
**Treatment 1**		
SCA1N14 NT	23.083.413	204.166.208
SCA1N14 KO	18.950.088	272.760.511
**Treatment 2**		
SCA1N14 NT	37.162.159	343.533.237
SCA1N14 KO	24.977.212	431.412.496
**Treatment 3**		
SCA1N14 NT	45.195.185	814.528.786
SCA1N14 KO	25.151.389	704.542.265

Figure S6. Effects of CRISPR/Cas9 system on ATXN1 expression in SCA1 fibroblasts.

U

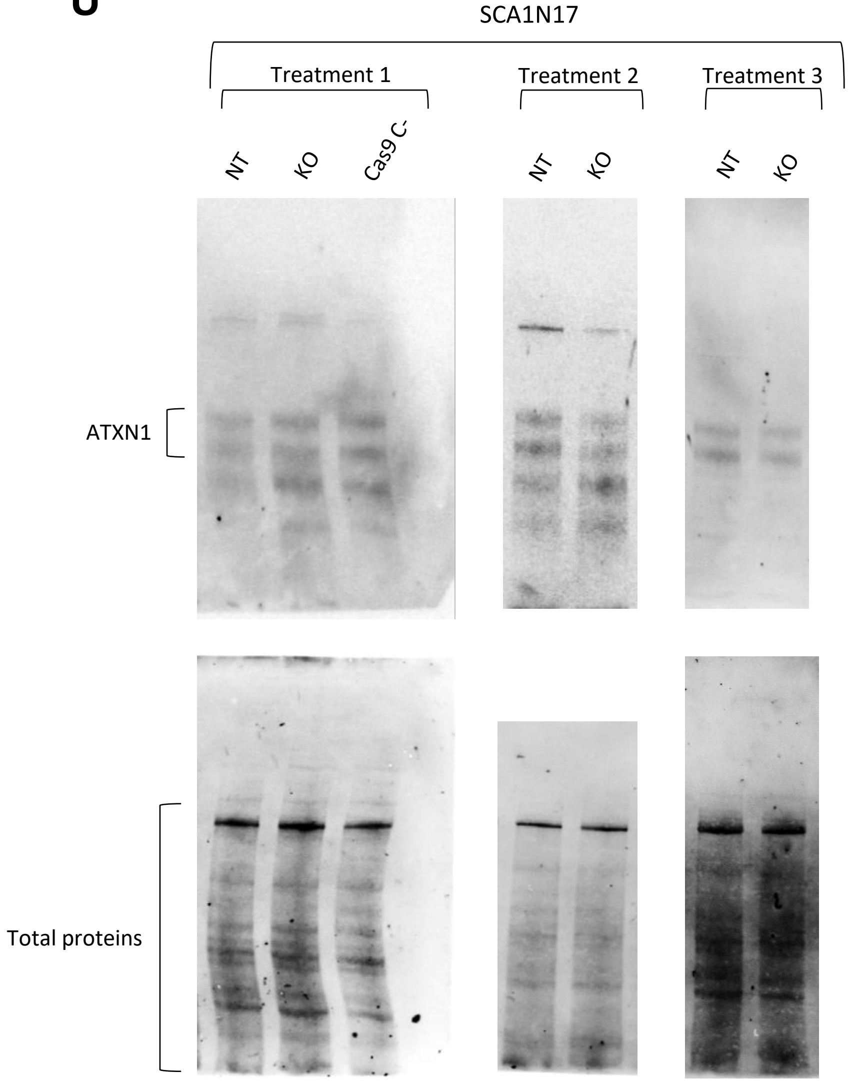

V

Samples	Adj. Vol. ATXN1	Adj. Vol. Total proteins
**Treatment 1**		
SCA1N17 NT	2.281.738	119.286.963
SCA1N17 KO	2.403.343	155.973.918
SCA1N17 Cas9 C-	2.555.294	131.758.662
**Treatment 2**		
SCA1N17 2T NT	1.586.436	65.727.533
SCA1N17 2T KO	676.037	60.286.332
**Treatment 3**		
SCA1N17 3T NT	1.707.583	403.262.895
SCA1N17 3T KO	1.343.606	385.239.664

Figure S6. Effects of CRISPR/Cas9 system on ATXN1 expression in SCA1 fibroblasts. Fibroblasts from patients SCA1N1 (**A,B**), SCA1N5 (**C,D**), SCA1N6 (**E,F**), SCA1N8 (**G,H**), SCA1N9 (**I,L**), SCA1N10 (**M,N**), SCA1N11 (**O,P**), SCA1N12 (**Q,R**), SCA1N14 (**S,T**), SCA1N17 (**U,V**) were treated using sgRNAs G3 and G8 complexed with Cas9 endonucleases and the ATXN1 expression was determined by Western Blotting. Raw data were obtained by densitometry of Western Blotting bands, using the Image Lab 6.0 software. Adjusted Volume means the background-adjusted volume, which is the sum of all the intensities within the band boundaries. Values are mean ± s.e.m. from three independent experiments. The statistical test used was unpaired t test with two-tailed P value and alpha level $P<0.05$. NT: untreated sample; KO: treated sample; Cas9 C-: sample treated with scramble sgRNA.
