# Supplementary material for: Suppressing gain-of-function proteins via CRISPR/Cas9 system in SCA1 cells
Source: Sci Rep. 2022 Nov 24;12:20285. doi: 10.1038/s41598-022-24299-y (PMC9700751; doi:10.1038/s41598-022-24299-y)
Supplement: Supplementary file 7 — Supplementary Figure S7. [file 41598_2022_24299_MOESM7_ESM.pdf]

A

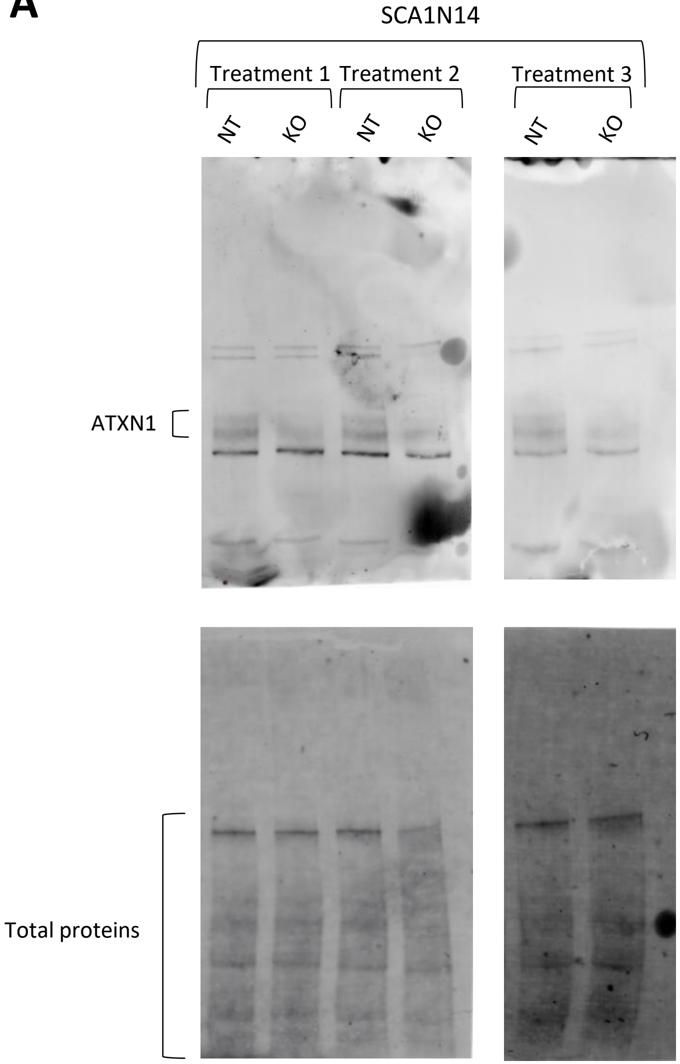

B

Samples	Adj. Vol. ATXN1	Adj. Vol. Total proteins
**Treatment 1**		
SCA1N14 NT	33.861.283	198.624.452
SCA1N14 KO	12.277.738	238.902.014
**Treatment 2**		
SCA1N14 NT	31.573.317	214.457.378
SCA1N14 KO	9.727.162	261.823.183
**Treatment 3**		
SCA1N14 NT	21.905.759	402.248.302
SCA1N14 KO	10.204.937	504.755.277

Figure S7. Effects of G3N/G8N-Cas9 RNP complexes on ATXN1 expression in SCA1 fibroblasts.

C

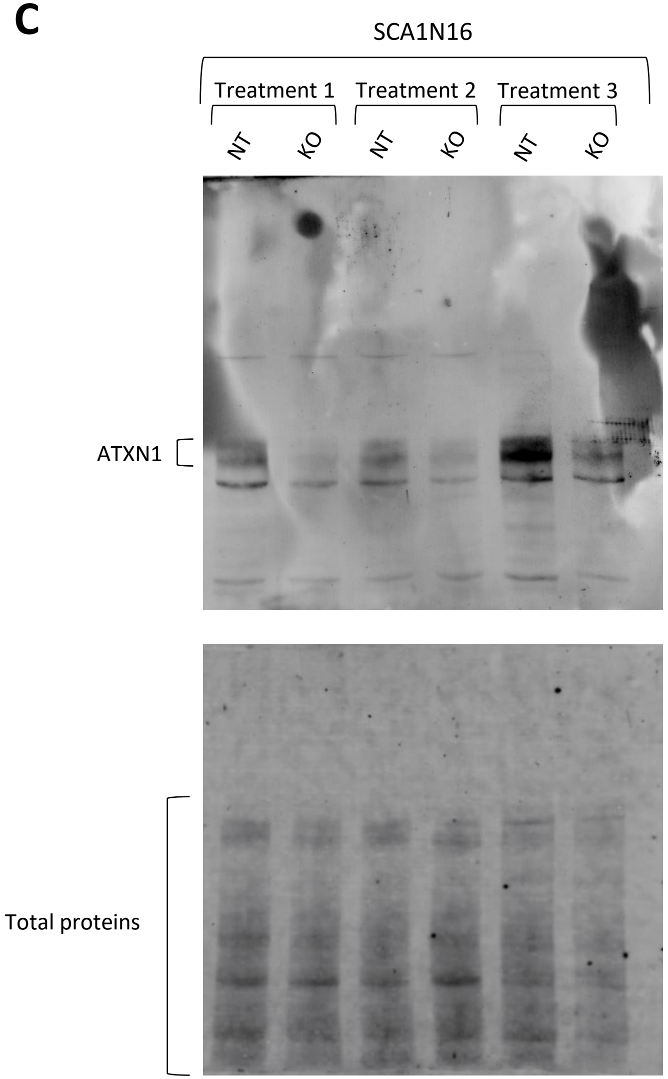

D

Samples	Adj. Vol. ATXN1	Adj. Vol. Total proteins
**Treatment 1**		
SCA1N16 NT	54.013.235	329.057.317
SCA1N16 KO	13.655.912	307.850.990
**Treatment 2**		
SCA1N16 NT	41.525.260	320.189.948
SCA1N16 KO	19.903.546	388.180.578
**Treatment 3**		
SCA1N16 NT	142.681.331	317.438.699
SCA1N16 KO	41.121.032	217.585.507

Figure S7. Effects of G3N/G8N-Cas9 RNP complexes on ATXN1 expression in SCA1 fibroblasts.

E

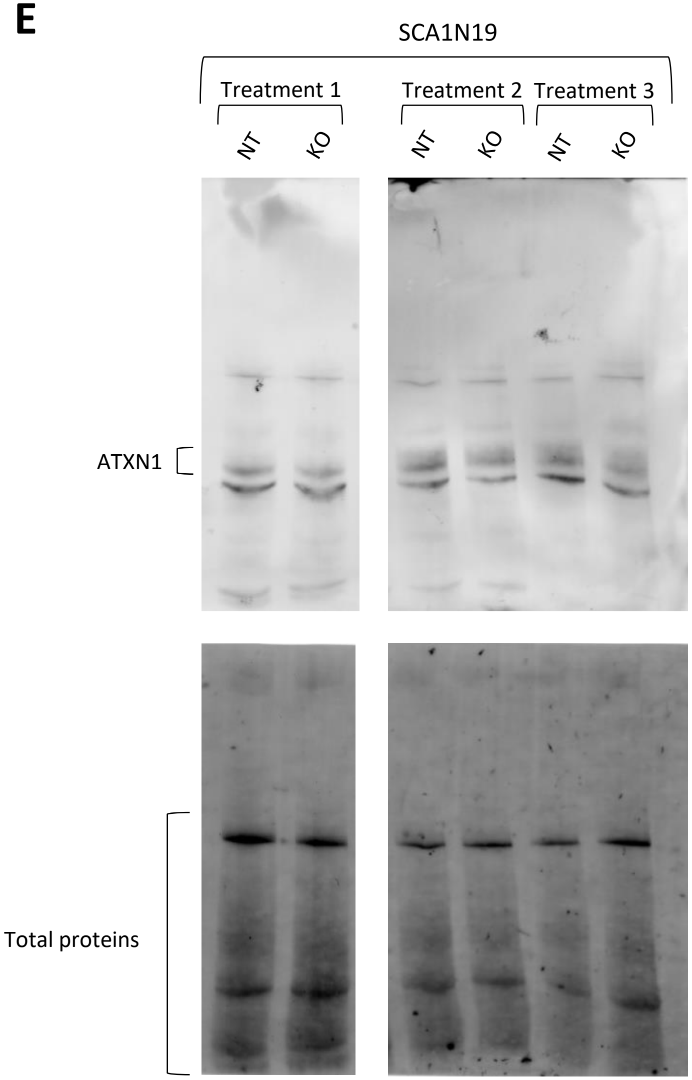

F

Samples	Adj. Vol. ATXN1	Adj. Vol. Total proteins
**Treatment 1**		
SCA1N19 NT	27.262.581	694.013.635
SCA1N19 KO	15.526.928	720.813.114
**Treatment 2**		
SCA1N19 NT	51.102.622	475.828.382
SCA1N19 KO	28.226.895	480.178.215
**Treatment 3**		
SCA1N19 NT	38.795.211	408.893.996
SCA1N19 KO	23.767.258	539.246.134

Figure S7. Effects of G3N/G8N-Cas9 RNP complexes on ATXN1 expression in SCA1 fibroblasts. Fibroblasts from patients SCA1N14 (**A,B**), SCA1N16 (**C,D**), SCA1N19 (**E,F**) were treated using sgRNAs G3N and G8N complexed with Cas9 endonucleases and the ATXN1 expression was determined by Western Blotting. Raw data were obtained by densitometry of Western Blotting bands, using the Image Lab 6.0 software. Adjusted Volume means the background-adjusted volume, which is the sum of all the intensities within the band boundaries. Values are mean ± s.e.m. from three independent experiments. The statistical test used was unpaired t test with two-tailed P value and alpha level $P<0.05$. NT: untreated sample; KO: treated sample; Cas9 C-: sample treated with scramble sgRNA.
