# Supplementary material for: Suppressing gain-of-function proteins via CRISPR/Cas9 system in SCA1 cells
Source: Sci Rep. 2022 Nov 24;12:20285. doi: 10.1038/s41598-022-24299-y (PMC9700751; doi:10.1038/s41598-022-24299-y)
Supplement: Supplementary file 8 — Supplementary Figure S8. [file 41598_2022_24299_MOESM8_ESM.pdf]

# A

| Samples | Adj. Vol. ATXN1 | Adj. Vol. Total Protein |
|---|---|---|
| **Treatment 1** | | |
| SCA1N1 NT M | 3.941.585 | 427.074.249 |
| SCA1N1 NT WT | 3.330.311 | |
| SCA1N1 KO M | 2.671.227 | 437.725.372 |
| SCA1N1 KO WT | 3.359.231 | |
| **Treatment 2** | | |
| SCA1N1 NT M | 4.530.608 | 552.354.179 |
| SCA1N1 NT WT | 2.429.979 | |
| SCA1N1 KO M | 2.635.067 | 451.201.779 |
| SCA1N1 KO WT | 1.579.377 | |
| **Treatment 3** | | |
| SCA1N1 NT M | 8.529.164 | 561.211.693 |
| SCA1N1 NT WT | 9.433.524 | |
| SCA1N1 KO M | 4.606.080 | 589.239.936 |
| SCA1N1 KO WT | 4.304.774 | |

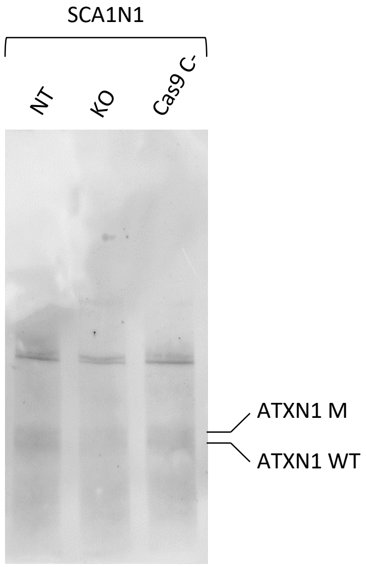


| Samples | Adj. Vol. ATXN1 | Adj. Vol. Total Protein |
|---|---|---|
| **Treatment 1** | | |
| SCA1N5 NT M | 3.696.301 | 642.613.237 |
| SCA1N5 NT WT | 7.517.746 | |
| SCA1N5 KO M | 2.201.872 | 759.380.948 |
| SCA1N5 KO WT | 5.541.146 | |
| **Treatment 2** | | |
| SCA1N5 NT M | 10.702.659 | 710.474.680 |
| SCA1N5 NT WT | 23.745.417 | |
| SCA1N5 KO M | 3.408.529 | 641.455.285 |
| SCA1N5 KO WT | 17.021.003 | |
| **Treatment 3** | | |
| SCA1N5 NT M | 21.389.196 | 703.475.170 |
| SCA1N5 NT WT | 34.750.481 | |
| SCA1N5 KO M | 6.653.340 | 768.194.824 |
| SCA1N5 KO WT | 8.905.871 | |

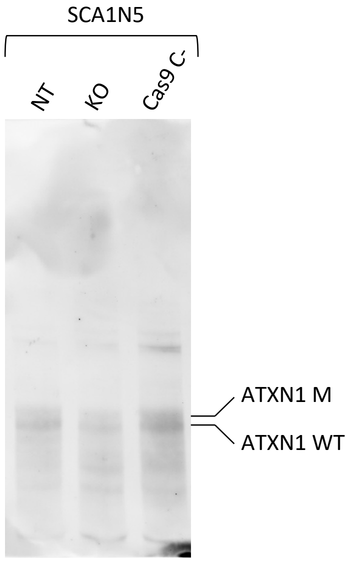


| Samples | Adj. Vol. ATXN1 | Adj. Vol. Total Protein |
|---|---|---|
| **Treatment 1** | | |
| SCA1N6 NT M | 7.505.218 | 113.671.538 |
| SCA1N6 NT WT | 8.939.601 | |
| SCA1N6 KO M | 4.025.320 | 107.977.561 |
| SCA1N6 KO WT | 5.928.647 | |
| **Treatment 2** | | |
| SCA1N6 NT M | 4.871.181 | 113.877.997 |
| SCA1N6 NT WT | 6.721.831 | |
| SCA1N6 KO M | 2.247.809 | 89.182.109 |
| SCA1N6 KO WT | 3.448.163 | |
| **Treatment 3** | | |
| SCA1N6 NT M | 647.787 | 271.310.565 |
| SCA1N6 NT WT | 790.038 | |
| SCA1N6 KO M | 436.707 | 278.979.304 |
| SCA1N6 KO WT | 590.195 | |

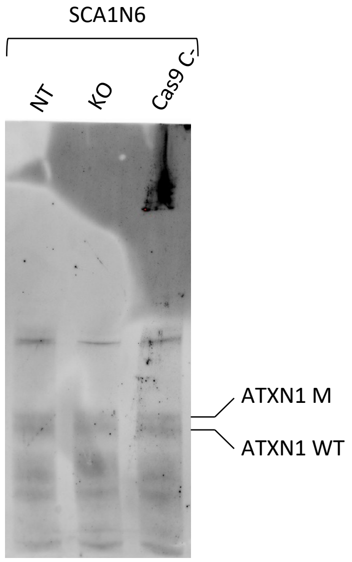


**Figure S8.** Raw data obtained by densitometry of Western Blotting bands.

| Samples | Adj. Vol. ATXN1 | Adj. Vol. Total Protein |
|---|---|---|
| **Treatment 1** | | |
| SCA1N8 NT M | 20.739.209 | 621.744.601 |
| SCA1N8 NT WT | 31.487.196 | |
| SCA1N8 KO M | 228.281 | 580.868.835 |
| SCA1N8 KO WT | 3.435.086 | |
| **Treatment 2** | | |
| SCA1N8 NT M | 33.504.883 | 659.367.492 |
| SCA1N8 NT WT | 54.182.941 | |
| SCA1N8 KO M | 1.376.896 | 505.317.194 |
| SCA1N8 KO WT | 5.848.644 | |
| **Treatment 3** | | |
| SCA1N8 NT M | 15.129.363 | 932.791.223 |
| SCA1N8 NT WT | 31.916.905 | |
| SCA1N8 KO M | 4.301.144 | 868.580.287 |
| SCA1N8 KO WT | 13.740.287 | |

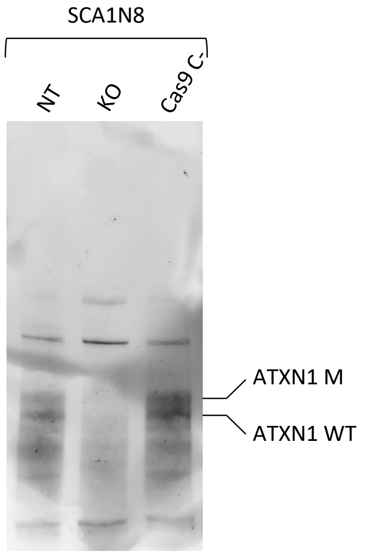


| Samples | Adj. Vol. ATXN1 | Adj. Vol. Total Protein |
|---|---|---|
| **Treatment 1** | | |
| SCA1N9 NT M | 11.944.463 | 319.128.450 |
| SCA1N9 NT WT | 9.500.506 | |
| SCA1N9 KO M | 3.120.987 | 325.982.139 |
| SCA1N9 KO WT | 3.754.825 | |
| **Treatment 2** | | |
| SCA1N9 NT M | 10.470.909 | 259.756.846 |
| SCA1N9 NT WT | 17.985.821 | |
| SCA1N9 KO M | 8.560.136 | 316.936.250 |
| SCA1N9 KO WT | 25.363.530 | |
| **Treatment 3** | | |
| SCA1N9 NT M | 14.511.690 | 226.324.410 |
| SCA1N9 NT WT | 20.052.547 | |
| SCA1N9 KO M | 11.462.718 | 297.253.724 |
| SCA1N9 KO WT | 14.762.722 | |

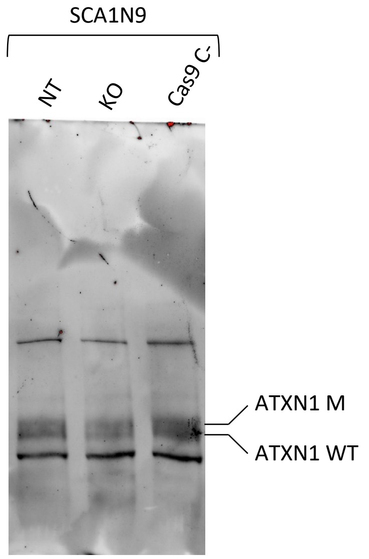


| Samples | Adj. Vol. ATXN1 | Adj. Vol. Total Protein |
|---|---|---|
| **Treatment 1** | | |
| SCA1N10 NT M | 17.903.729 | 839.941.885 |
| SCA1N10 NT WT | 43.532.839 | |
| SCA1N10 KO M | 11.521.410 | 888.419.004 |
| SCA1N10 KO WT | 38.443.730 | |
| **Treatment 2** | | |
| SCA1N10 NT M | 14.416.784 | 772.893.905 |
| SCA1N10 NT WT | 39.313.501 | |
| SCA1N10 KO M | 11.589.687 | 813.343.880 |
| SCA1N10 KO WT | 42.041.288 | |
| **Treatment 3** | | |
| SCA1N10 NT M | 2.621.993 | 680.769.039 |
| SCA1N10 NT WT | 20.342.595 | |
| SCA1N10 KO M | 2.435.796 | 1.062.595.389 |
| SCA1N10 KO WT | 27.036.968 | |

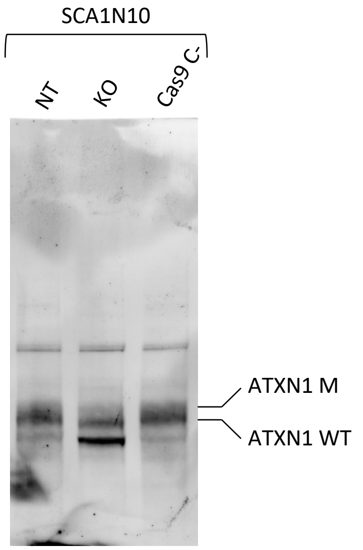


**Figure S8.** Raw data obtained by densitometry of Western Blotting bands.

| Samples | Adj. Vol. ATXN1 | Adj. Vol. Total Protein |
|---|---|---|
| **Treatment 1** | | |
| SCA1N11 NT M | 11.728.884 | 426.528.261 |
| SCA1N11 NT WT | 14.489.655 | |
| SCA1N11 KO M | 10.281.377 | 440.670.192 |
| SCA1N11 KO WT | 15.342.736 | |
| **Treatment 2** | | |
| SCA1N11 NT M | 6.225.366 | 707.167.925 |
| SCA1N11 NT WT | 13.179.002 | |
| SCA1N11 KO M | 6.507.696 | 933.825.406 |
| SCA1N11 KO WT | 16.321.338 | |
| **Treatment 3** | | |
| SCA1N11 NT M | 2.732.554 | 704.024.157 |
| SCA1N11 NT WT | 13.512.601 | |
| SCA1N11 KO M | 2.499.633 | 781.457.184 |
| SCA1N11 KO WT | 7.390.966 | |

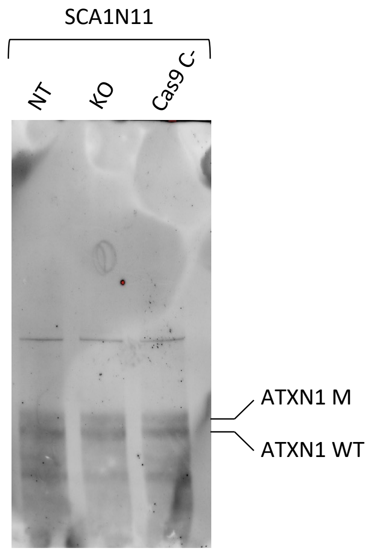


| Samples | Adj. Vol. ATXN1 | Adj. Vol. Total Protein |
|---|---|---|
| **Treatment 1** | | |
| SCA1N12 NT M | 19.755.779 | 378.007.481 |
| SCA1N12 NT WT | 37.710.065 | |
| SCA1N12 KO M | 19.942.356 | 642.514.633 |
| SCA1N12 KO WT | 27.501.992 | |
| **Treatment 2** | | |
| SCA1N12 NT M | 23.457.233 | 443.672.852 |
| SCA1N12 NT WT | 41.541.831 | |
| SCA1N12 KO M | 16.812.695 | 446.981.180 |
| SCA1N12 KO WT | 33.594.041 | |
| **Treatment 3** | | |
| SCA1N12 NT M | 20.178.590 | 585.731.248 |
| SCA1N12 NT WT | 30.784.389 | |
| SCA1N12 KO M | 10.500.302 | 489.413.763 |
| SCA1N12 KO WT | 24.130.235 | |

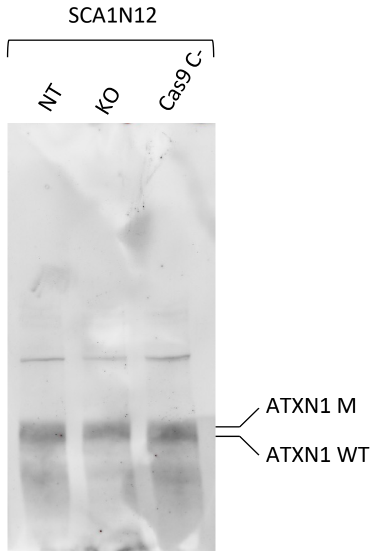


| Samples | Adj. Vol. ATXN1 | Adj. Vol. Total Protein |
|---|---|---|
| **Treatment 1** | | |
| SCA1N14 1T NT M | 3.172.221 | 204.166.208 |
| SCA1N14 1T NT WT | 10.428.217 | |
| SCA1N14 1T KO M | 1.428.740 | 272.760.511 |
| SCA1N14 1T KO WT | 10.462.019 | |
| **Treatment 2** | | |
| SCA1N14 2T NT M | 2.354.080 | 343.533.237 |
| SCA1N14 2T NT WT | 12.609.440 | |
| SCA1N14 2T KO M | 712.984 | 431.412.496 |
| SCA1N14 2T KO WT | 12.098.587 | |
| **Treatment 3** | | |
| SCA1N14 3T NT M | 10.193.338 | 814.528.786 |
| SCA1N14 3T NT WT | 14.134.800 | |
| SCA1N14 3T KO M | 4.123.250 | 704.542.265 |
| SCA1N14 3T KO WT | 13.420.163 | |

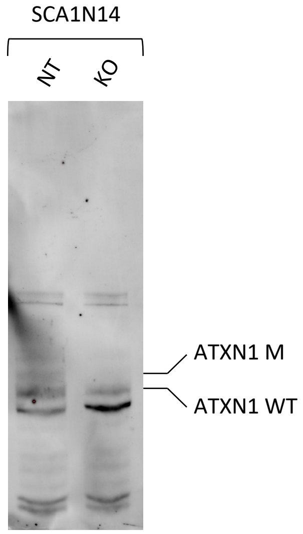


**Figure S8.** Raw data obtained by densitometry of Western Blotting bands.

| Samples | Adj. Vol. ATXN1 | Adj. Vol. Total Protein |
|---|---|---|
| **Treatment 1** | | |
| SCA1N17 NT M | 540.397 | 119.286.963 |
| SCA1N17 NT WT | 443.573 | |
| SCA1N17 KO M | 824.172 | 155.973.918 |
| SCA1N17 KO WT | 593.645 | |
| **Treatment 2** | | |
| SCA1N17 NT M | 421.316 | 65.727.533 |
| SCA1N17 NT WT | 499.140 | |
| SCA1N17 KO M | 273.771 | 60.286.332 |
| SCA1N17 KO WT | 319.189 | |
| **Treatment 3** | | |
| SCA1N17 NT M | 359.564 | 403.262.895 |
| SCA1N17 NT WT | 459.064 | |
| SCA1N17 KO M | 300.154 | 385.239.664 |
| SCA1N17 KO WT | 410.043 | |

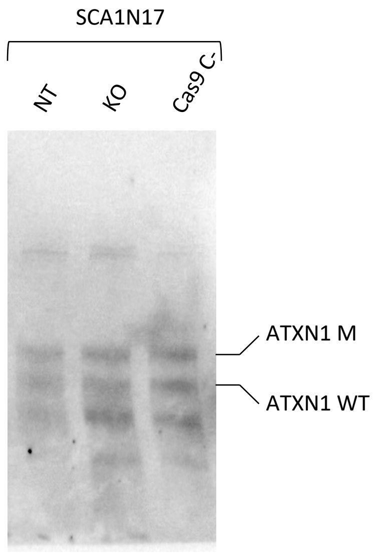


**Figure S8.** Raw data obtained by densitometry of Western Blotting bands.

**B**

| Samples | Adj. Vol. ATXN1 | Adj. Vol. Total Protein |
|---|---|---|
| **Treatment 1** | | |
| SCA1N14 NT M | 3.057.226 | 198.624.452 |
| SCA1N14 NT WT | 10.245.390 | |
| SCA1N14 KO M | 464.353 | 238.902.014 |
| SCA1N14 KO WT | 4.069.869 | |
| **Treatment 2** | | |
| SCA1N14 NT M | 2.650.822 | 214.457.378 |
| SCA1N14 NT WT | 10.857.969 | |
| SCA1N14 KO M | 879.784 | 261.823.183 |
| SCA1N14 KO WT | 6.238.124 | |
| **Treatment 3** | | |
| SCA1N14 NT M | 8.036.112 | 402.248.302 |
| SCA1N14 NT WT | 13.838.522 | |
| SCA1N14 KO M | 3.078.626 | 504.755.277 |
| SCA1N14 KO WT | 16.896.110 | |

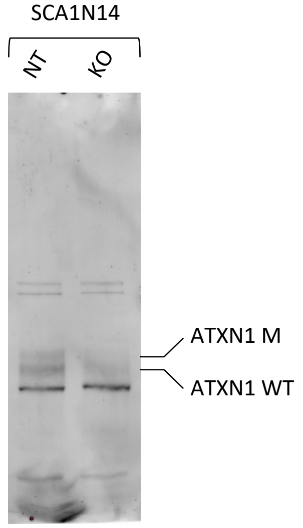


| Samples | Adj. Vol. ATXN1 | Adj. Vol. Total Protein |
|---|---|---|
| **Treatment 1** | | |
| SCA1N16 NT M | 9.731.993 | 329.057.317 |
| SCA1N16 NT WT | 17.516.522 | |
| SCA1N16 KO M | 1.968.865 | 307.850.990 |
| SCA1N16 KO WT | 4.473.212 | |
| **Treatment 2** | | |
| SCA1N16 NT M | 7.222.601 | 320.189.948 |
| SCA1N16 NT WT | 11.533.984 | |
| SCA1N16 KO M | 3.287.987 | 388.180.578 |
| SCA1N16 KO WT | 5.601.027 | |
| **Treatment 3** | | |
| SCA1N16 NT M | 22.270.927 | 317.438.699 |
| SCA1N16 NT WT | 44.853.767 | |
| SCA1N16 KO M | 6.340.869 | 217.585.507 |
| SCA1N16 KO WT | 14.546.246 | |

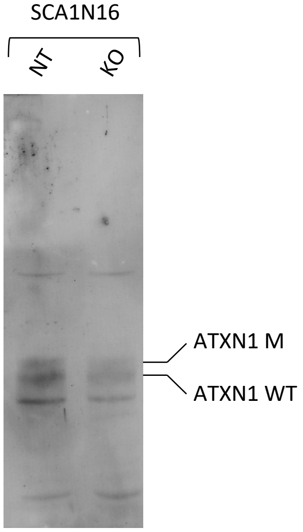


| Samples | Adj. Vol. ATXN1 | Adj. Vol. Total Protein |
|---|---|---|
| **Treatment 1** | | |
| SCA1N19 NT M | 648.996 | 694.013.635 |
| SCA1N19 NT WT | 17.434.105 | |
| SCA1N19 KO M | 217.249 | 720.813.114 |
| SCA1N19 KO WT | 12.158.614 | |
| **Treatment 2** | | |
| SCA1N19 NT M | 5.715.926 | 475.828.382 |
| SCA1N19 NT WT | 18.558.710 | |
| SCA1N19 KO M | 3.183.166 | 480.178.215 |
| SCA1N19 KO WT | 15.128.387 | |
| **Treatment 3** | | |
| SCA1N19 NT M | 5.285.120 | 408.893.996 |
| SCA1N19 NT WT | 11.010.793 | |
| SCA1N19 KO M | 4.169.871 | 539.246.134 |
| SCA1N19 KO WT | 10.302.845 | |

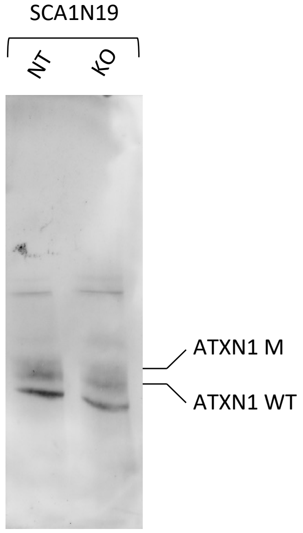


**Figure S8.** Raw data obtained by densitometry of the two Western Blotting bands formed by healthy and mutated ataxin 1. Densitometry was performed using the Image Lab 6.0 software. Adjusted Volume means the background-adjusted volume, which is the sum of all the intensities within the band boundaries. An example of bands obtained by the Western Blotting assay is reported for each table. M: upper band consisting of mutated ataxin 1; WT: lower band consisting of healthy ataxin 1.
