# Supplementary material for: Suppressing gain-of-function proteins via CRISPR/Cas9 system in SCA1 cells
Source: Sci Rep. 2022 Nov 24;12:20285. doi: 10.1038/s41598-022-24299-y (PMC9700751; doi:10.1038/s41598-022-24299-y)
Supplement: Supplementary file 9 — Supplementary Figure S9. [file 41598_2022_24299_MOESM9_ESM.pdf]

Exon 8 with the cutting sites of G3N, G3 and G8 sgRNAs and the fragments obtained from the first PCRs (regular) and from nested.

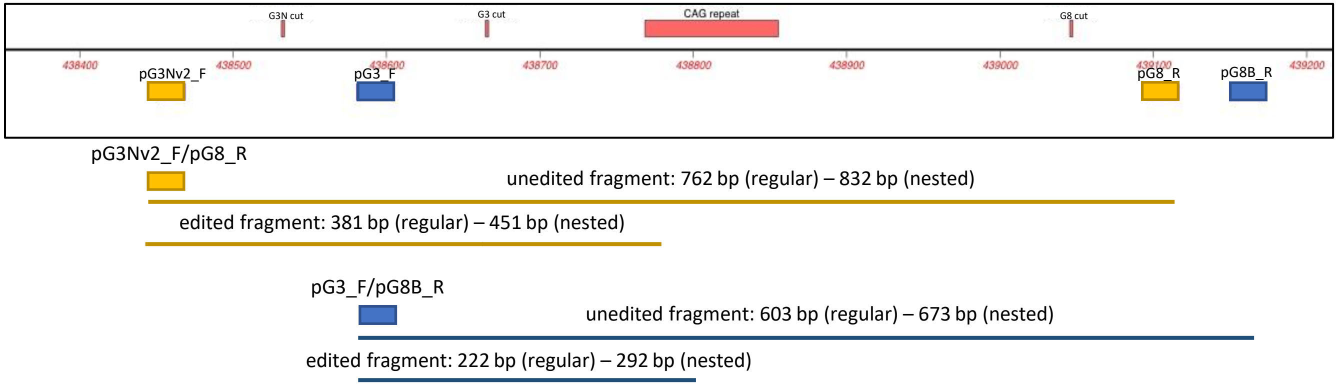

Exon 9 with the cutting site of the G8N sgRNA and the fragments obtained from the first PCR (regular) and from nested.

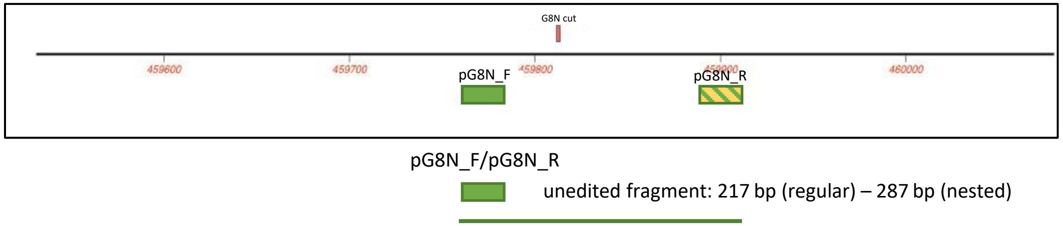

Simulation of the structure of the large deletion between G3N and G8N cutting sites and the fragments obtained from the first PCR (regular) and from nested.

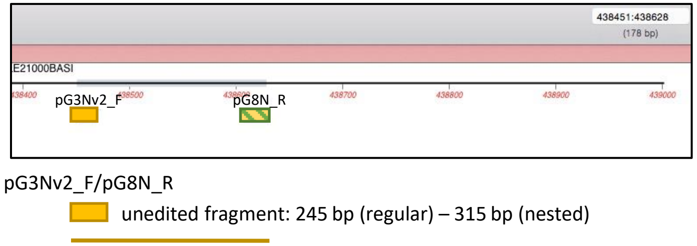

Figure S9. NGS amplicons simulation and PCR fragments size. Simulation of the amplicons obtained with the primers designed for on-target NGS analysis. The protospacer of G3, G8, G3N, and G8N sgRNAs are also indicated. Fragment sizes are reported for both the first PCRs and the nested.
