# Supplementary material for: Suppressing gain-of-function proteins via CRISPR/Cas9 system in SCA1 cells
Source: Sci Rep. 2022 Nov 24;12:20285. doi: 10.1038/s41598-022-24299-y (PMC9700751; doi:10.1038/s41598-022-24299-y)
Supplement: Supplementary file 10 — Supplementary Figure S10. [file 41598_2022_24299_MOESM10_ESM.pdf]

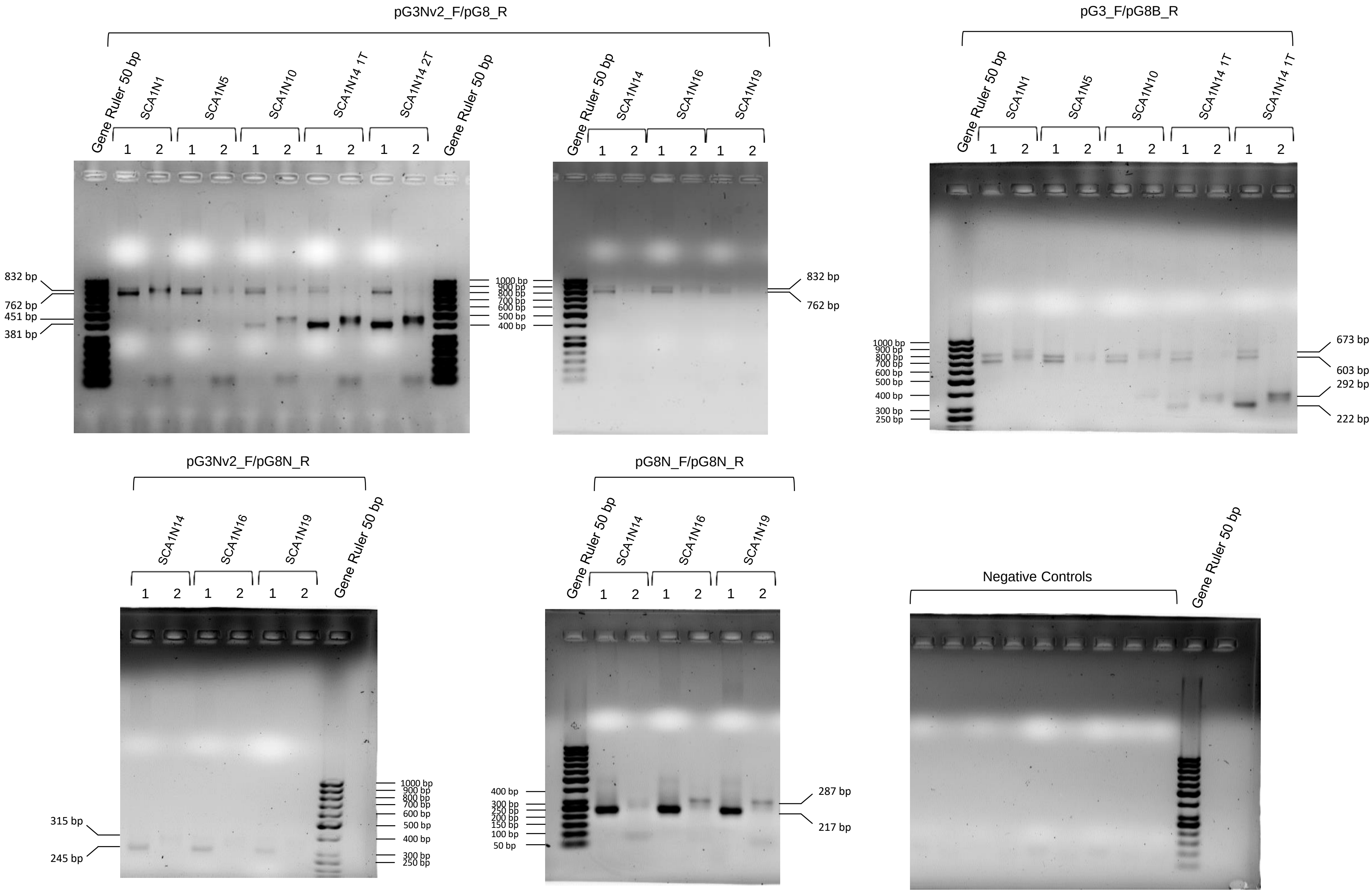

Figure S10. NGS libraries. The DNA of fibroblasts treated with both the RNP complexes G3sgRNA/Cas9 and G8sgRNA/Cas9 (SCA1N1, SCA1N5, SCA1N10, SCA1N14 1T, SCA1N14 2T, where 1T stands for first treatment and 2T stands for second treatment) and with the G3NsgRNA/Cas9 and G8NsgRNA/Cas9 RNPs (SCA1N14, SCA1N16, SCA1N19). The primers used are shown in table 3 for the initial PCR (indicated with 1 in the figure) and in table 5 for the nested PCR (indicated with 2 in the figure). DNA ladder: GeneRuler 50 bp DNA Ladder (50 bp, 100 bp, 150 bp, 200 bp, 250 bp, 300 bp, 400 bp, 500 bp, 600 bp, 700 bp, 800 bp, 900 bp, 1000 bp).
