# Supplementary material for: Suppressing gain-of-function proteins via CRISPR/Cas9 system in SCA1 cells
Source: Sci Rep. 2022 Nov 24;12:20285. doi: 10.1038/s41598-022-24299-y (PMC9700751; doi:10.1038/s41598-022-24299-y)
Supplement: Supplementary file 11 — Supplementary Figure S11. [file 41598_2022_24299_MOESM11_ESM.pdf]

A

LOCATION WITH RESPECT TO THE CUTTING SITE	SCA1N5					
	G3			G8		
	SNPs	INDELS	READS	SNPs	INDELS	READS
	%	%	Total Count	%	%	Total Count
-10	11	0	3202	1	0,03	8649
-9	1	0	3202	2	0,03	8626
-8	8	0,03	3205	1	0,05	8618
-7	10	0,03	3230	0	0,07	8618
-6	11	0,03	3232	1	0,07	8605
-5	10	0,06	3231	1	0,07	8684
-4	6	0,06	3235	0	0,08	8505
-3	9	0,06	3296	0	0,15	8475
-2	6	0,06	6454	1	0,15	8379
-1	5	0,09	6518	0	0,2	8350
+1	1	0,08	7469	0	0,4	8283
+2	4	0,1	7486	2	0,2	4181
+3	3	0,07	7544	1	0,2	4138
+4	4	0,06	7572	1	0,2	4138
+5	7	0,07	7811	1	0,2	4131
+6	3	0,05	7884	0	0,2	4136
+7	2	0,05	7892	1	0,1	4138
+8	5	0,06	7892	1	0,1	4137
+9	3	0,06	7933	4	0,1	4135
+10	5	0,08	7952	9	0,2	4140

B

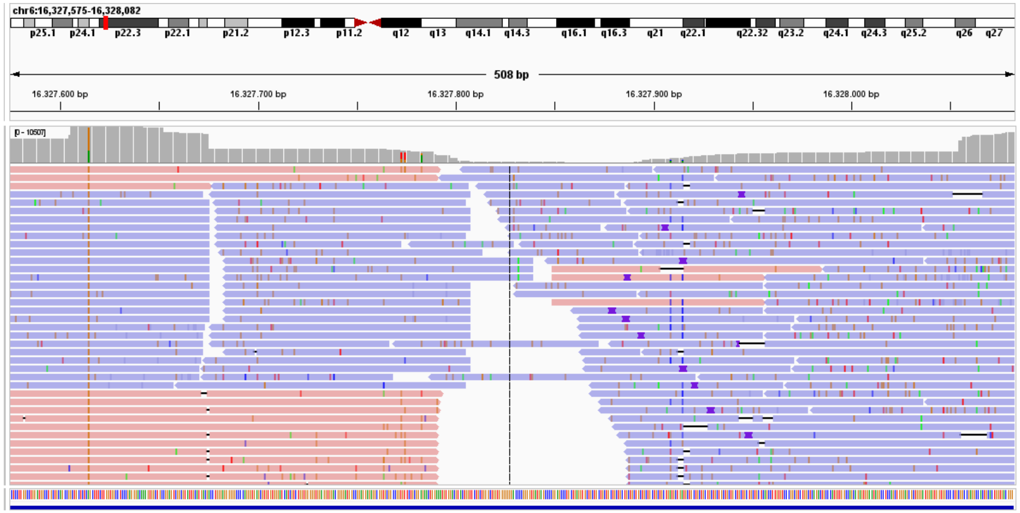

Figure S11. Results of the NGS analysis using the FreeBayes algorithm.

C

LOCATION WITH RESPECT TO THE CUTTING SITE	SCA1N10					
	G3			G8		
	SNPs	INDELS	READS	SNPs	INDELS	READS
	%	%	Total Count	%	%	Total Count
-10	14	0,06	21471	3	0,04	337294
-9	3	0,08	21490	1	0,05	336066
-8	13	0,16	21807	0	0,08	335327
-7	15	0,13	21862	1	0,09	332783
-6	15	0,05	21873	1	0,1	328814
-5	15	0,5	21921	0	0,1	314099
-4	10	0,6	22211	0	0,2	312793
-3	10	0,6	27624	0	0,2	300035
-2	2	0,08	265373	0	0,2	294241
-1	1	0,08	271980	0	0,3	286914
+1	0	0,06	297602	2	0,8	51427
+2	1	0,03	299378	0	0,6	49108
+3	1	0,01	305858	1	0,7	44426
+4	1	0,008	310964	2	0,6	43489
+5	6	0,009	321752	0	0,6	43404
+6	1	0,006	324384	0	0,6	43276
+7	1	0,005	326669	0	0,5	43232
+8	3	0,007	328258	2	0,6	36113
+9	3	0,008	331235	0	0,8	36055
+10	4	0,006	334141	1	0,5	36132

D

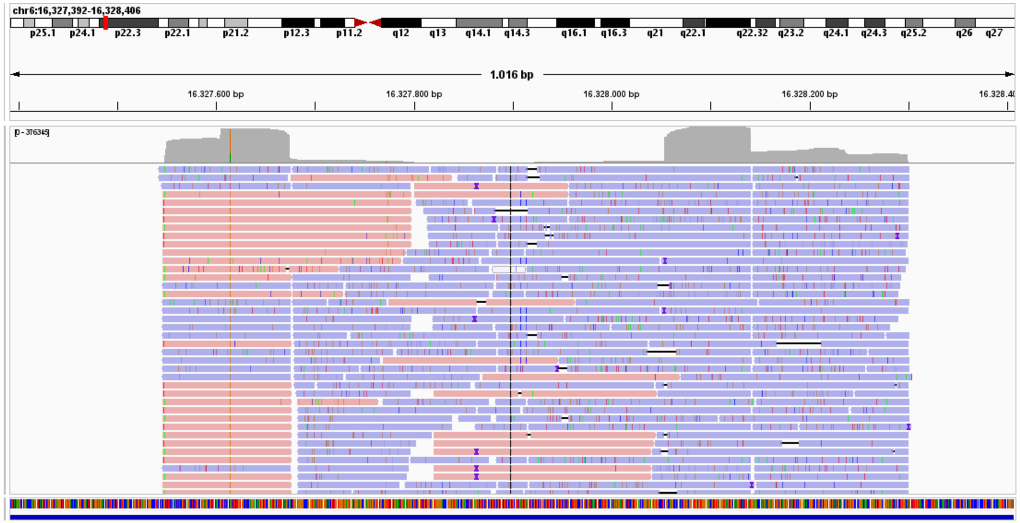

Figure S11. Results of the NGS analysis using the FreeBayes algorithm.

E

LOCATION WITH RESPECT TO THE CUTTING SITE	SCA1N14 1T G3-G8					
	G3			G8		
	SNPs	INDELS	READS	SNPs	INDELS	READS
	%	%	Total Count	%	%	Total Count
-10	6	0	175	2	0	330314
-9	2	0	175	1	0	328511
-8	3	0	175	0	0,0006	327240
-7	5	0	183	1	0	325014
-6	8	0	211	1	0,0003	320635
-5	3	0,46	215	0	0,006	311163
-4	7	0.16	608	0	0,008	309549
-3	1	0,05	9424	0	0,009	294842
-2	3	0,006	279036	0	0,009	288072
-1	0	0,0017	289108	0	0,008	288734
+1	0	0,0093	332593	3	0,06	6617
+2	0	0,027	335087	1	0,09	3469
+3	0	0,007	345832	1	0,04	2408
+4	0	0,0006	349293	3	0,06	1619
+5	7	0,0005	366346	0	0,06	1601
+6	1	0	371835	1	0,06	1584
+7	0	0,0003	373497	0	0,06	1573
+8	4	0,003	374315	4	0,07	1439
+9	3	0,0016	382229	0	0,07	1432
+10	5	0,00026	386746	1	0,07	1433

F

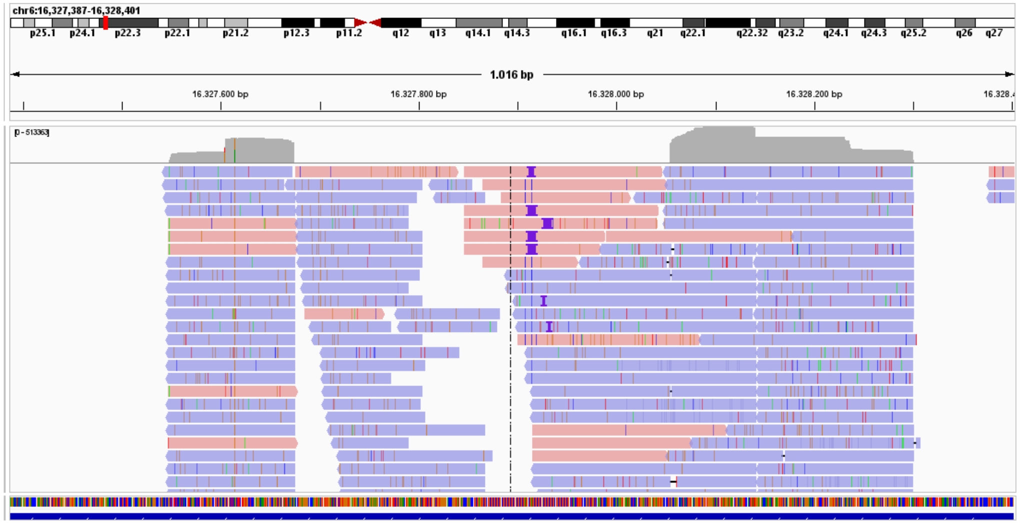

Figure S11. Results of the NGS analysis using the FreeBayes algorithm.

G

LOCATION WITH RESPECT TO THE CUTTING SITE	SCA1N14 2T G3-G8					
	G3			G8		
	SNPs	INDELS	READS	SNPs	INDELS	READS
	%	%	Total Count	%	%	Total Count
-10	8	0,66	756	3	0,003	381784
-9	1	0,79	756	1	0,002	378603
-8	2	0,78	773	0	0,004	376933
-7	6	1,25	802	1	0,004	373956
-6	5	1,18	935	2	0,0046	369447
-5	6	2,55	940	0	0,0047	358751
-4	5	2,14	1167	0	0,018	356965
-3	4	0,43	7909	0	0,019	339570
-2	2	0,03	326411	0	0,02	332648
-1	0	0,03	336764	0	0,02	326169
+1	0	0,01	389786	1	0,4	6663
+2	0	0,028	391845	1	0,7	2642
+3	0	0,01	403934	0	0,99	1404
+4	0	0,007	407779	2	1,3	1035
+5	9	0,006	440551	0	1,2	1034
+6	9	0,006	440551	2	1,1	995
+7	0	0,004	447419	0	0,9	992
+8	2	0,005	448018	3	0,9	959
+9	1	0,004	452324	0	1,6	967
+10	4	0,003	456056	1	0,9	962

H

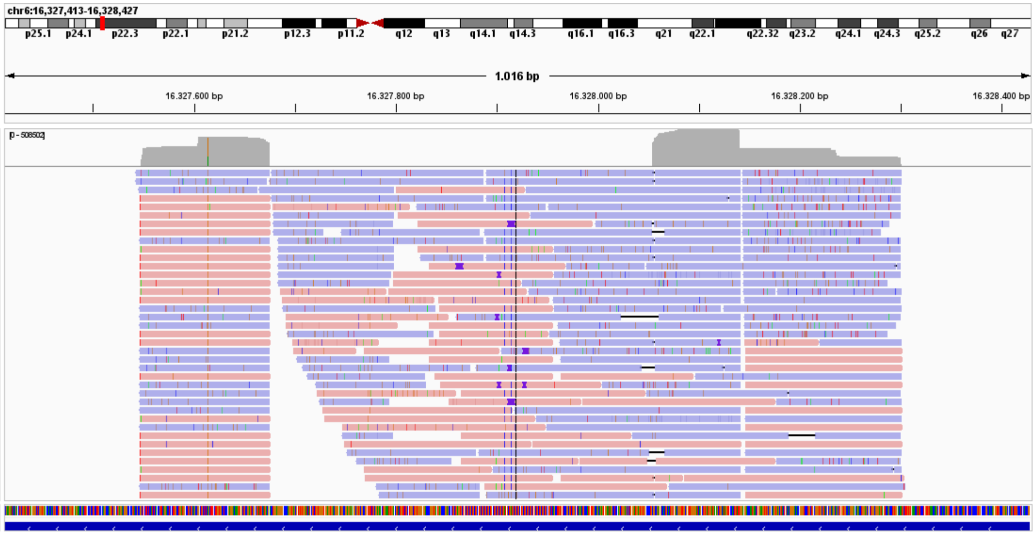

Figure S11. Results of the NGS analysis using the FreeBayes algorithm.

I

LOCATION WITH RESPECT TO THE CUTTING SITE		SCA1N14 G3N-G8N					
		G3N			G8N		
		SNPs	INDELS	READS	SNPs	INDELS	READS
		%	%	Total Count	%	%	Total Count
	-10	1	0,7	11568	0	0,08	523570
	-9	3	0,8	11540	2	0,09	522832
	-8	1	0,9	11528	1	0,09	519945
	-7	4	1	11515	2	0,1	519179
	-6	4	1,6	11443	1	0,2	518353
	-5	2	1,9	11418	2	0,2	516826
	-4	2	2,08	11415	1	0,3	512717
	-3	1	3,4	11702	0	0,4	510894
	-2	3	2,2	23286	1	2,3	495573
	-1	1	1,5	53859	0	0,8	495372
	+1	0	0,14	233617	0	1,4	341447
	+2	0	0,06	246857	0	1,9	311352
	+3	0	0,04	250889	1	1,06	274565
	+4	0	0,04	252095	0	0,4	276446
	+5	3	0,04	253438	3	0,3	276458
	+6	0	0,04	254341	1	0,3	276255
	+7	2	0,03	254951	1	0,3	276425
	+8	1	0,03	255701	1	0,2	276511
	+9	2	0,03	256290	3	0,3	276204
	+10	1	0,03	257460	1	0,15	276568

L

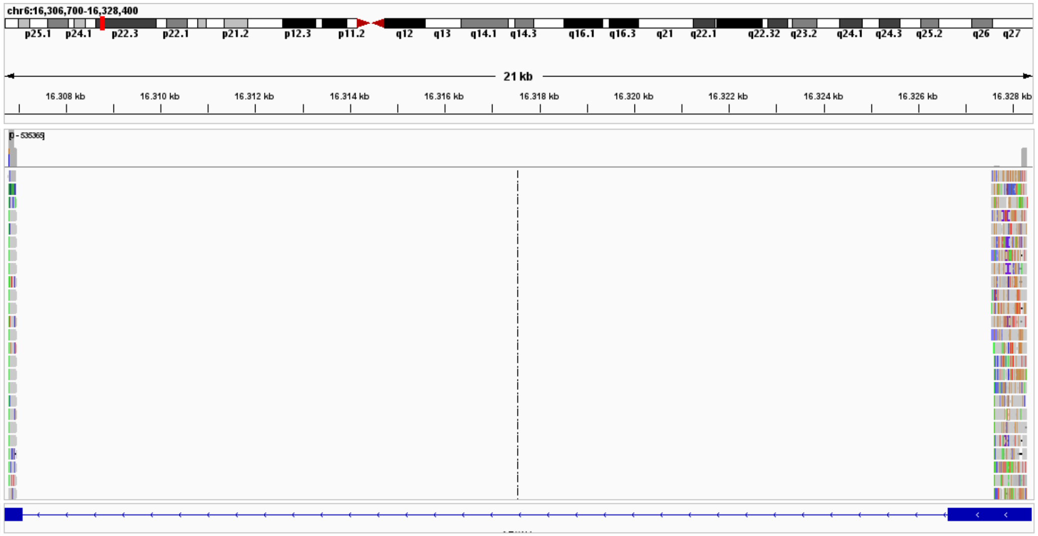

Figure S11. Results of the NGS analysis using the FreeBayes algorithm.

M

LOCATION WITH RESPECT TO THE CUTTING SITE	SCA1N16 G3N-G8N					
	G3N			G8N		
	SNPs	INDELS	READS	SNPs	INDELS	READS
	%	%	Total Count	%	%	Total Count
-10	1	0,1	22278	1	0,1	503119
-9	1	0,1	22277	2	0,1	502962
-8	1	0,2	22274	2	0,1	501776
-7	4	0,2	22268	1	0,1	500686
-6	2	0,2	22252	1	0,2	499810
-5	1	0,2	22376	2	0,2	499091
-4	2	0,3	22399	1	0,2	495770
-3	1	0,4	22513	0	0,3	494939
-2	4	0,5	28621	2	2,3	482225
-1	2	0,5	49195	0	0,7	486909
+1	0	0,05	163340	0	1,1	390849
+2	0	0,04	168631	1	1,6	375266
+3	0	0,02	169651	1	0,7	349502
+4	0	0,02	172932	0	0,2	351121
+5	3	0,02	173668	3	0,2	351215
+6	0	0,02	177220	1	0,2	351023
+7	2	0,01	177417	3	0,1	351180
+8	1	0,01	178134	1	0,1	351270
+9	2	0,01	178388	3	0,2	350862
+10	1	0,01	179331	1	0,08	351317

N

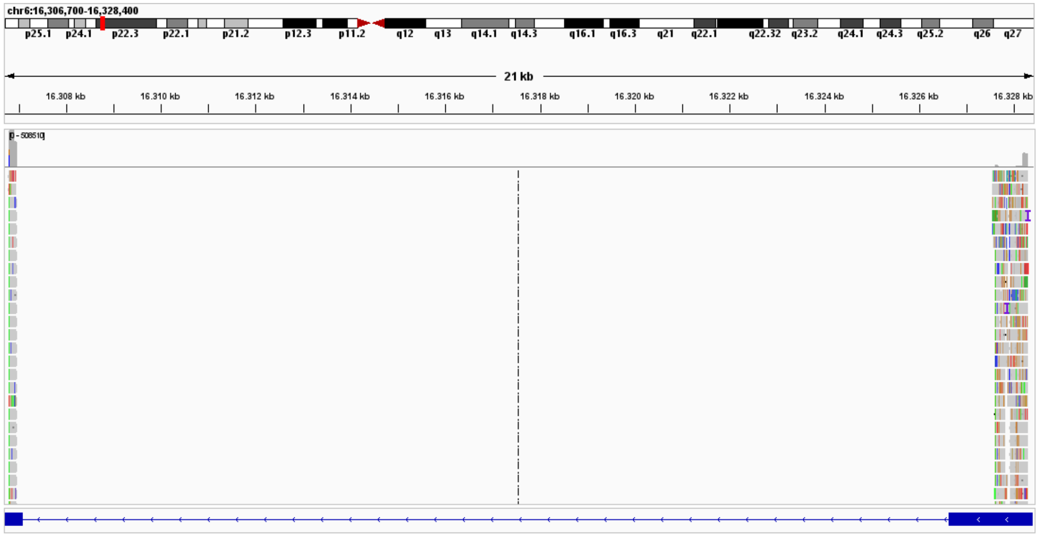

Figure S11. Results of the NGS analysis using the FreeBayes algorithm.

O

LOCATION WITH RESPECT TO THE CUTTING SITE	SCA1N19					
	G3N			G8N		
	SNPs	INDELS	READS	SNPs	INDELS	READS
	%	%	Total Count	%	%	Total Count
-10	1	0,1	24875	0	0,02	418639
-9	2	0,1	24874	2	0,02	418508
-8	1	0,1	24874	3	0,02	417432
-7	4	0,1	24872	1	0,03	417176
-6	2	0,2	24863	1	0,05	416683
-5	1	0,2	24855	2	0,05	415684
-4	2	0,2	24863	1	0,06	411906
-3	1	0,3	25968	0	0,08	411159
-2	4	0,3	27527	1	0,7	407502
-1	2	0,4	44028	0	0,1	408525
+1	0	0,4	135624	0	0,3	334903
+2	0	0,04	137452	1	0,4	330230
+3	0	0,03	138841	1	0,2	305301
+4	0	0,03	139083	0	0,09	304670
+5	3	0,02	139338	3	0,09	305659
+6	0	0,02	139632	2	0,1	305495
+7	2	0,02	139728	3	0,07	305540
+8	1	0,02	139911	1	0,06	305562
+9	2	0,02	140022	3	0,1	305146
+10	1	0,02	141028	1	0,05	305557

P

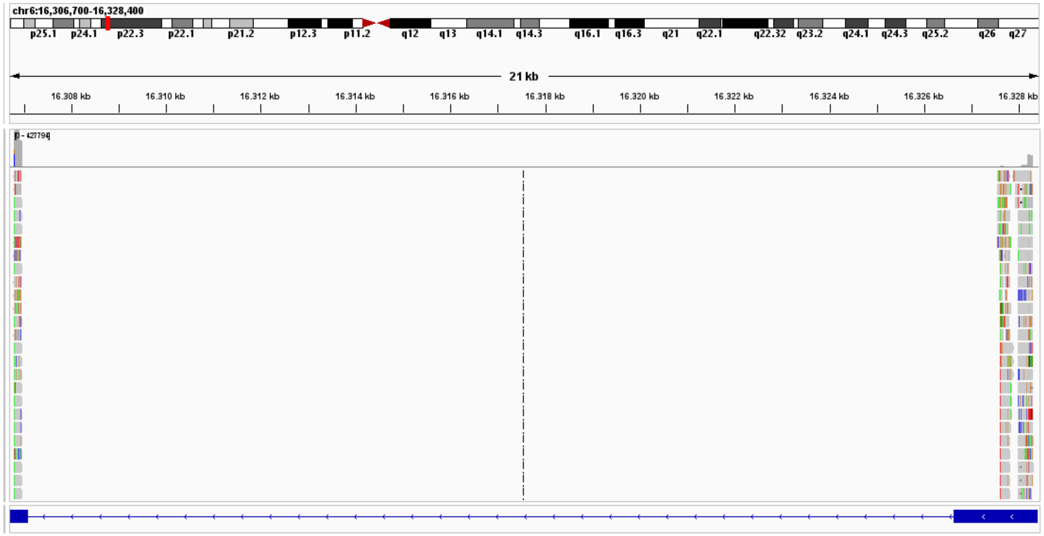

Figure S11. Results of the NGS analysis using the FreeBayes algorithm. The tables show the percentages of SNP and indel and the total number of aligned reads of the region comprised between 10 nucleotides upstream (-10) and downstream (+10) of the cutting sites of the G3sgRNA/Cas9 and G8sgRNA/Cas9 (**A,C,E,G**) and G3NsgRNA/Cas9 and G8NsgRNA/Cas9 (**I,M,O**) complexes. The alignments of the reads obtained with IGV tool are also reported for the same complexes: **B,D,F,H** for G3-G8sgRNAs/Cas9 and **L,N,P** for G3N-G8NsgRNA/Cas9 RNPs.
