# Supplementary material for: Suppressing gain-of-function proteins via CRISPR/Cas9 system in SCA1 cells
Source: Sci Rep. 2022 Nov 24;12:20285. doi: 10.1038/s41598-022-24299-y (PMC9700751; doi:10.1038/s41598-022-24299-y)
Supplement: Supplementary file 12 — Supplementary Figure S12. [file 41598_2022_24299_MOESM12_ESM.pdf]

A

LOCATION WITH RESPECT TO THE CUTTING SITE		BCOR		
		**SNPs**	**INDELS**	**READS**
		%	%	Total Count
	-10	0	0	30
	-9	0	0	30
	-8	0	0	30
	-7	3	0	30
	-6	0	0	30
	-5	0	0	30
	-4	0	0	30
	-3	0	0	30
	-2	0	0	30
	-1	0	0	30
	+1	0	0	30
	+2	0	0	30
	+3	0	0	30
	+4	0	0	30
	+5	0	0	30
	+6	0	0	30
	+7	3	0	30
	+8	0	0	30
	+9	0	0	30
	+10	0	0	30

B

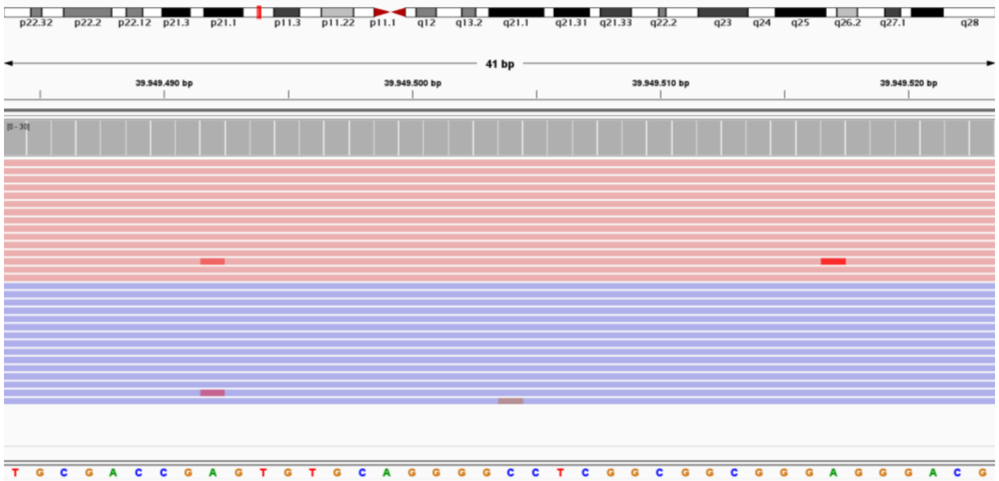

Figure S12. Results of the off-target NGS analysis using the FreeBayes algorithm.

C

LOCATION WITH RESPECT TO THE CUTTING SITE	INPP5A		
	SNPs	INDELS	READS
	%	%	Total Count
-10	0,05	0,009	44356
-9	0,03	0,009	44372
-8	0,04	0,009	44365
-7	0,13	0,009	44352
-6	0,1	0,009	44342
-5	0,06	0,009	44332
-4	0,05	0,009	44335
-3	0,09	0,009	44343
-2	0,09	0	44372
-1	0,03	0	44380
+1	0,1	0	44387
+2	0,03	0	44396
+3	0,07	0	44399
+4	0,06	0,004	44400
+5	0,06	0,004	44453
+6	0,06	0,004	44485
+7	0,07	0,004	44504
+8	0,09	0,004	44528
+9	0,18	0,004	44555
+10	0,03	0,004	44602

D

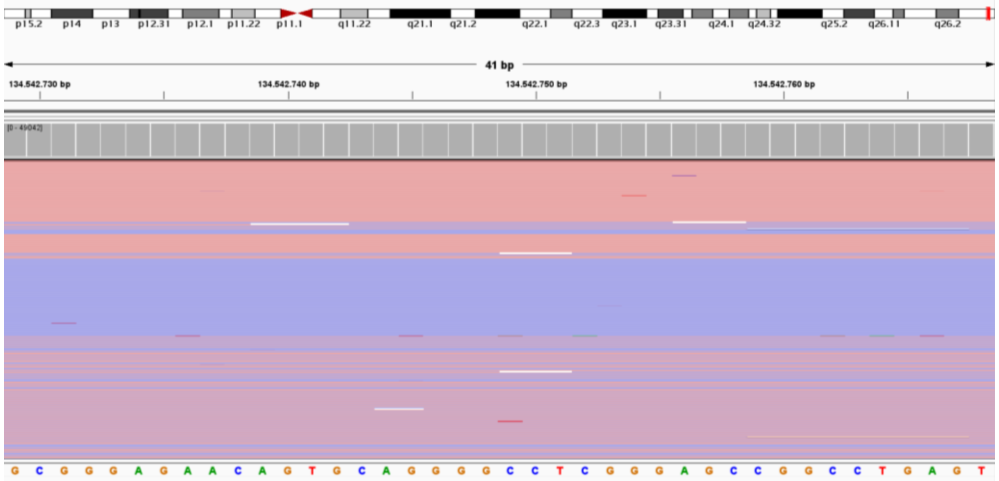

Figure S12. Results of the off-target NGS analysis using the FreeBayes algorithm.

E

LOCATION WITH RESPECT TO THE CUTTING SITE	ZNRF1		
	SNPs	INDELS	READS
	%	%	Total Count
-10	0,84	0	44906
-9	0,24	0	44925
-8	0,14	0	44947
-7	0,93	0	44956
-6	0,1	0	44958
-5	0,17	0	44970
-4	0,14	0	44980
-3	0,97	0	44997
-2	0,09	0	45019
-1	0,19	0	45013
+1	0,25	0	45032
+2	0,12	0	45050
+3	0,16	0	45086
+4	0,35	0	45120
+5	0,06	0	45150
+6	0,7	0	45157
+7	0,18	0	45187
+8	0,16	0	45211
+9	0,12	0	45233
+10	0,31	0	45230

F

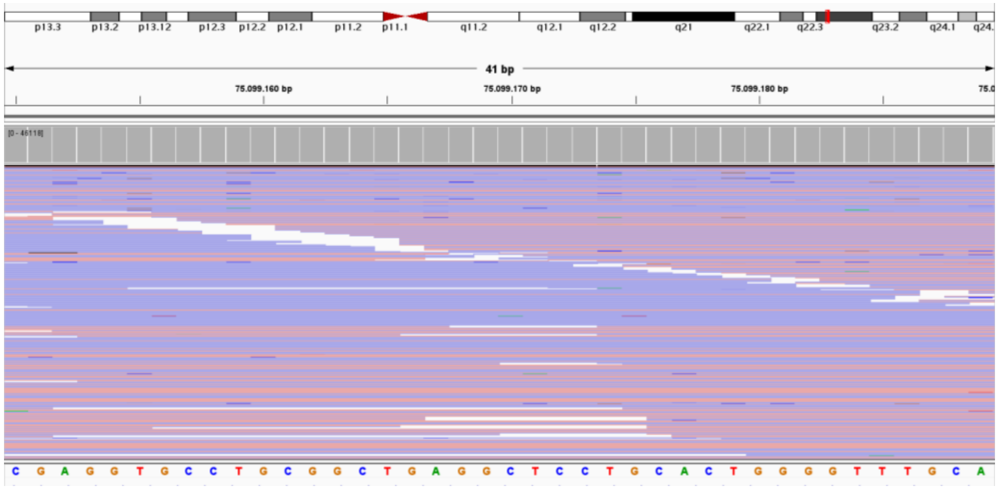

Figure S12. Results of the off-target NGS analysis using the FreeBayes algorithm.

G

LOCATION WITH RESPECT TO THE CUTTING SITE		COL3A		
		SNPs	INDELS	READS
		%	%	Total Count
	-10	1,19	0	2183
	-9	0,37	0	2189
	-8	0,18	0	2189
	-7	0	0	2184
	-6	0,14	0	2190
	-5	0,32	0	2206
	-4	0,05	0	2212
	-3	0,18	0	2242
	-2	0,13	0	2250
	-1	0,09	0	2259
	+1	0,22	0	2281
	+2	0,31	0	2287
	+3	0,04	0	2313
	+4	0,38	0	2345
	+5	0,13	0	2360
	+6	0,13	0	2368
	+7	0,17	0	2395
	+8	0,41	0	2419
	+9	0,2	0	2445
	+10	0,04	0	2480

H

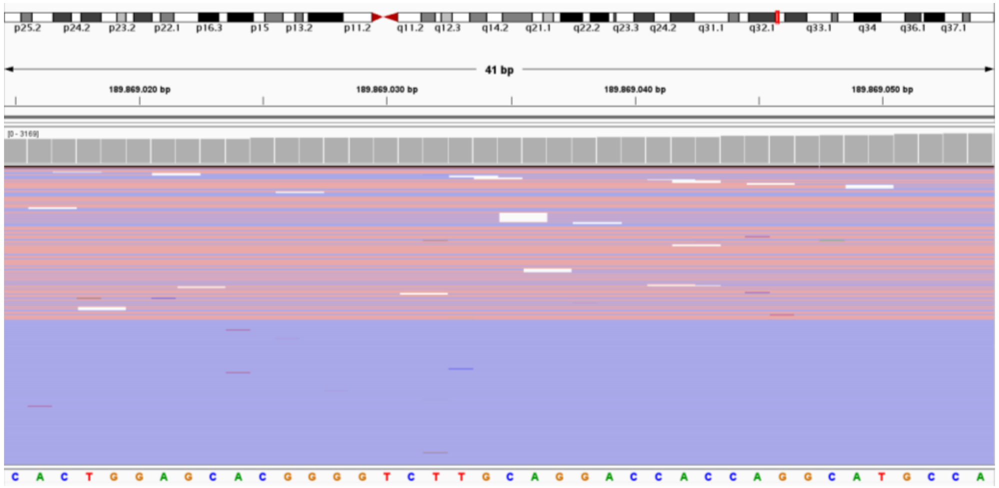

Figure S12. Results of the off-target NGS analysis using the FreeBayes algorithm.

I

LOCATION WITH RESPECT TO THE CUTTING SITE	KCNQ1		
	**SNPs**	**INDELS**	**READS**
	%	%	Total Count
-10	0,5	0,003	77272
-9	0,14	0,003	77251
-8	0,18	0,003	77267
-7	0,1	0,003	77273
-6	0,24	0,003	77263
-5	0,36	0,003	77243
-4	0,12	0,003	77232
-3	0,2	0,003	77227
-2	0,18	0,003	77233
-1	0,34	0,003	77251
+1	0,14	0,003	77247
+2	0,1	0,003	77267
+3	0,12	0,003	77290
+4	0,17	0,004	77279
+5	0,17	0,004	77263
+6	0,23	0,004	77255
+7	0,4	0,004	77242
+8	0,14	0,006	77237
+9	0,16	0,004	77217
+10	0,39	0,004	77208

L

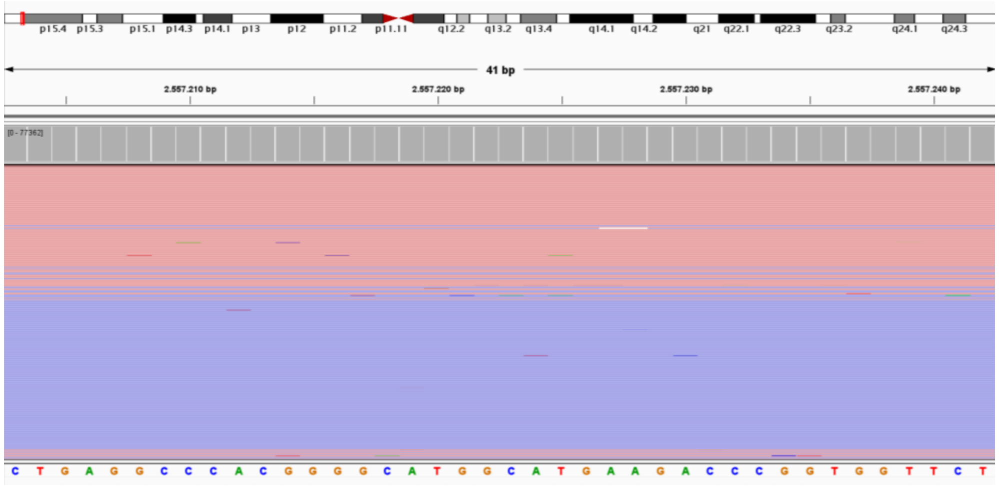

Figure S12. Results of the off-target NGS analysis using the FreeBayes algorithm.

M

LOCATION WITH RESPECT TO THE CUTTING SITE	MEIS1		
	SNPs	INDELS	READS
	%	%	Total Count
-10	0,1	0	39698
-9	0,14	0	39650
-8	0,12	0	39648
-7	0,11	0	39622
-6	0,15	0	39624
-5	0,12	0	39608
-4	0,1	0	39538
-3	0,06	0	39522
-2	0,07	0	39503
-1	0,15	0	39487
+1	0,06	0	39447
+2	0,05	0	39411
+3	0,05	0	39387
+4	0,09	0	39352
+5	0,09	0	39322
+6	0,07	0	39272
+7	0,1	0	39223
+8	0,1	0	39200
+9	0,07	0	39142
+10	0,06	0	39126

N

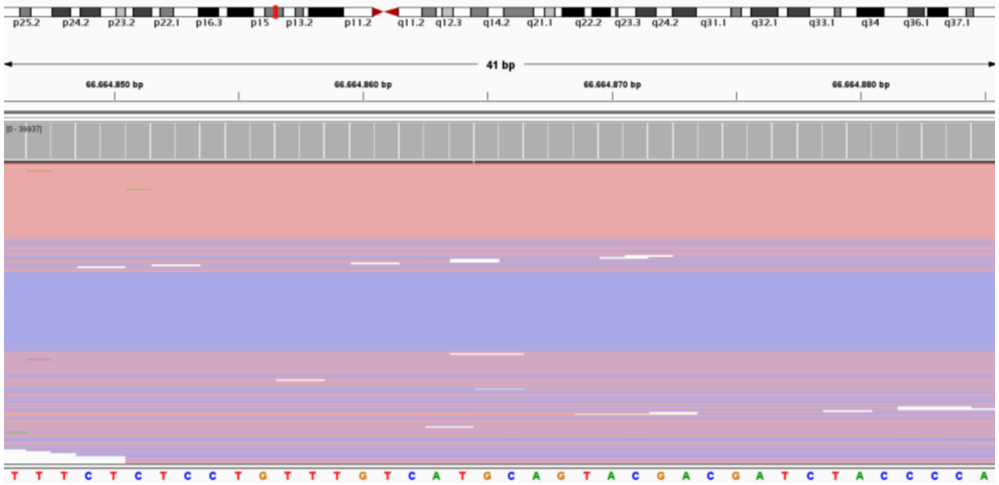

Figure S12. Results of the off-target NGS analysis using the FreeBayes algorithm. The tables show the percentages of SNP and indel and the total number of aligned reads of the region comprised between 10 nucleotides upstream (-10) and downstream (+10) of the hypothetical cutting sites (**A,C,E,G,L,M**). The alignments of the reads obtained with IGV tool are also reported for the same off-target sites (**B,D,F,H,L,N**).
