# Supplementary material for: Suppressing gain-of-function proteins via CRISPR/Cas9 system in SCA1 cells
Source: Sci Rep. 2022 Nov 24;12:20285. doi: 10.1038/s41598-022-24299-y (PMC9700751; doi:10.1038/s41598-022-24299-y)
Supplement: Supplementary file 13 — Supplementary Figure S13. [file 41598_2022_24299_MOESM13_ESM.pdf]

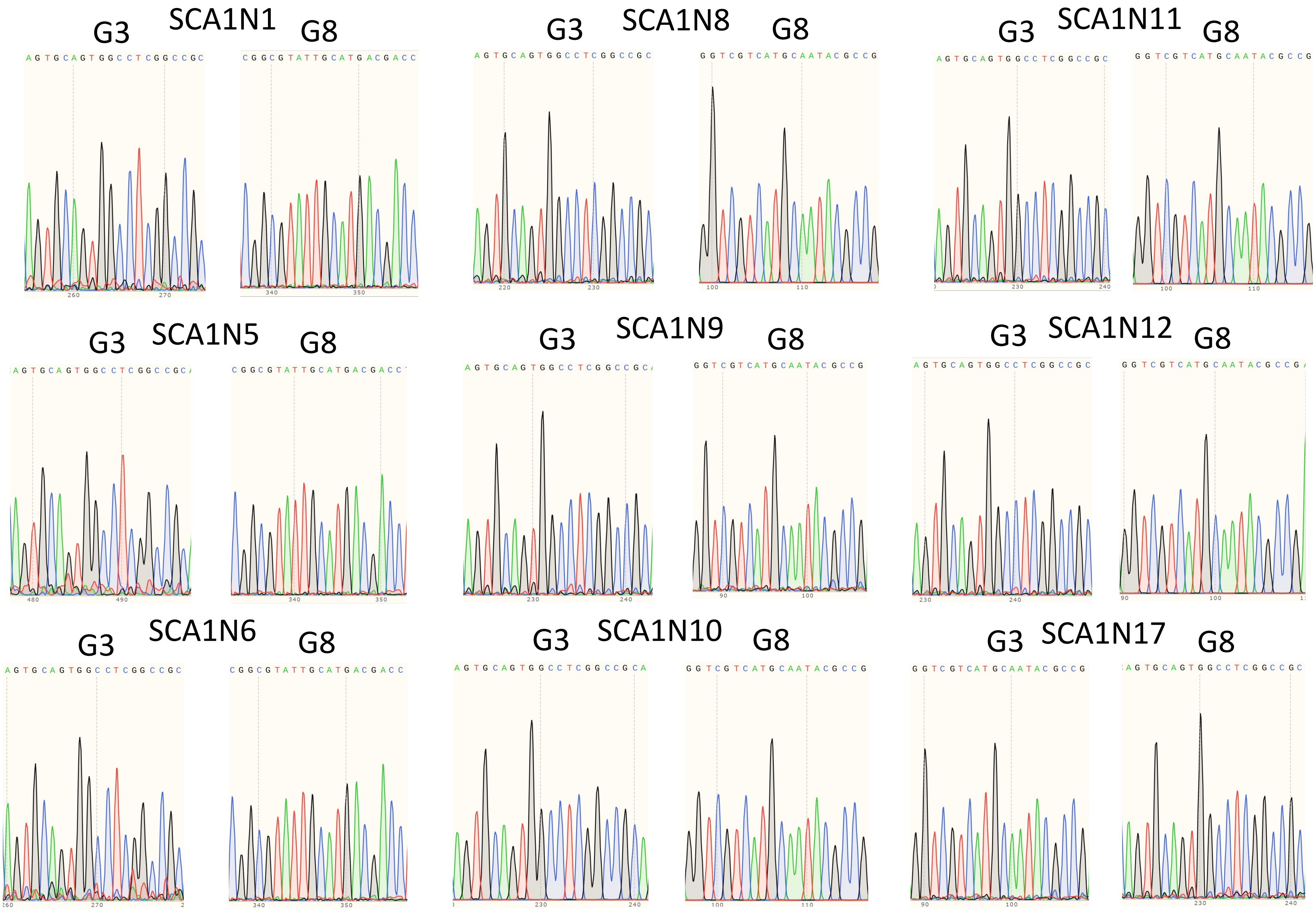

Figure S13. Sequencing of patients' G3 and G8 protospacers. G3 and G8 protospacers of SCA1N1, SCA1N5, SCA1N6, SCA1N8, SCA1N9, SCA1N10, SCA1N11, SCA1N12, SCA1N17 patients were sequenced by Sanger method (using forward or reverse primers) to confirm the absence of genetic modifications.
