# Supplementary material for: Suppressing gain-of-function proteins via CRISPR/Cas9 system in SCA1 cells
Source: Sci Rep. 2022 Nov 24;12:20285. doi: 10.1038/s41598-022-24299-y (PMC9700751; doi:10.1038/s41598-022-24299-y)
Supplement: Supplementary file 14 — Supplementary Tables. [file 41598_2022_24299_MOESM14_ESM.pdf]

Supplementary Material and Methods Section

**Table S1. Forward primers to produce the sgRNA *in vitro* transcription template.** Each primer contains the T7 promoter sequence, the spacer sequence (different for each sgRNA) and the scaffold template-annealing sequence.			
**sgRNA name**	**T7 promoter sequence**	**Spacer sequence**	**Guide-it Scaffold Template-annealing sequence**
G3	GCGGCCTCTAATACGACTCACTATAGGG	AGTGCAGTGGCCTCGGCCGC	GTTTTAGAGCTAGAAATAGCA
G4	GCGGCCTCTAATACGACTCACTAT AGGG	GGGCAGTCTGAGCCAGACGC	GTTTTAGAGCTAGAAATAGCA
G5	GCGGCCTCTAATACGACTCACTATAGGG	GTCATGCAATACGCCGACTC	GTTTTAGAGCTAGAAATAGCA
G6	GCGGCCTCTAATACGACTCACTATAGGG	GCCGGTGTTCTGCGGAGAAC	GTTTTAGAGCTAGAAATAGCA
G7	GCGGCCTCTAATACGACTCACTATAGGG	TGGAGAAGAGCCGGCGGTAC	GTTTTAGAGCTAGAAATAGCA
G8	GCGGCCTCTAATACGACTCACTATAGGG	CGGCGTATTGCATGACGACC	GTTTTAGAGCTAGAAATAGCA
G9	GCGGCCTCTAATACGACTCACTATAGGG	AGGACGGGGCCCCGTACCGC	GTTTTAGAGCTAGAAATAGCA
G10	GCGGCCTCTAATACGACTCACTATAGGG	GCGTGTGTGGGATCATCGTC	GTTTTAGAGCTAGAAATAGCA
G11	GCGGCCTCTAATACGACTCACTATAGGG	CAGCCTTGTGTCCCGGCGTC	GTTTTAGAGCTAGAAATAGCA
G12	GCGGCCTCTAATACGACTCACTATAGGG	TGGTGACGGGGTTGGCGGTT	GTTTTAGAGCTAGAAATAGCA

**Table S2. Primers for PCR reactions.** For each primer are reported the nucleotide sequence, the Melting temperature and what reaction it was used for.			
**Name**	**Sequence**	**Tm**	**Use**
TempForward	GAATGCCTGCCTCCCAAGAA	57,8	Cutting template production
TempReverse	AGTTTGGAACACGGCAAATCA	55,5	
BCOR-NGS-Forw	GGAGGACTGGAAACCCT	55	Amplicon NGS off-target
BCOR-NGS-Rev	TTCCGGGAACTACCTACAC	57	
INNP5A-NGS-Forw	TATGAAGGACTGCAAGGC	54	
INNP5A-NGS-Rev	CTGTGTGAGAGGAACTCTTTA	57	
ZNRF1-NGS-Forw	CCTGCAGGAATGTAGAATGG	58	
ZNRF1-NGS-Rev	CACTGCTACTACAAAGGTGTAA	58	
G8_COL3A1-Forw	TTGTAGGGTAACCCAGGACC	56,1	
G8_COL3A1-Rev	CCTGGCTAATTTACTCCAGTGAC	55,2	
KCNQ1-NGS-Forw	TTGCTTTGAGGGCTGTTTAG	56	
KCNQ1-NGS-Rev	GGACCAGCCTTCCTTCA	55	
MEIS1-NGS-Forw	GAAGGACCCAGCTGTATTG	57	
MEIS1-NGS-Rev	CCATGGCGTTGGTATGAG	56	

**Table S3. Primers for first step PCR reactions for libraries production.** For each primer are reported the adaptor, spacer and locus sequences.			
**Name**	**Adaptor**	**Spacer**	**Locus**
G3Nv2_1F	GTCTCGTGGGCTCGGAGATGTGTATAAGAGACAG		TGTCCACAGGGCTGGACTACT
G3Nv2_2F	GTCTCGTGGGCTCGGAGATGTGTATAAGAGACAG	ACATT	TGTCCACAGGGCTGGACTACT
G3Nv2_3F	GTCTCGTGGGCTCGGAGATGTGTATAAGAGACAG	CAGGGCACTTA	TGTCCACAGGGCTGGACTACT
G8_1R	TCGTCGGCAGCGTCAGATGTGTATAAGAGACAG	GCT	GCTCTCAGCTTTCTTGGT
G8_2R	TCGTCGGCAGCGTCAGATGTGTATAAGAGACAG	TGCT	GCTCTCAGCTTTCTTGGT
G8_3R	TCGTCGGCAGCGTCAGATGTGTATAAGAGACAG	CAGATAGCT	GCTCTCAGCTTTCTTGGT
G3_1F	GTCTCGTGGGCTCGGAGATGTGTATAAGAGACAG		AATACAGTGGAACCTATGCCAGC
G3_2F	GTCTCGTGGGCTCGGAGATGTGTATAAGAGACAG	CTCG	AATACAGTGGAACCTATGCCAGC
G3_3F	GTCTCGTGGGCTCGGAGATGTGTATAAGAGACAG	TCGCTGAG	AATACAGTGGAACCTATGCCAGC
G8B_1R	TCGTCGGCAGCGTCAGATGTGTATAAGAGACAG		TCTTCTCCATCTCACCGTTCA
G8B_2R	TCGTCGGCAGCGTCAGATGTGTATAAGAGACAG	CTA	CTCTTCTCCATCTCACCGTTCA
G8B_3R	TCGTCGGCAGCGTCAGATGTGTATAAGAGACAG	AGCGAACGTAG	CTCTTCTCCATCTCACCGTTCA
G8N_1F	GTCTCGTGGGCTCGGAGATGTGTATAAGAGACAG		ATCTCGCTTACCCTCAAGAACCT
G8N_2F	GTCTCGTGGGCTCGGAGATGTGTATAAGAGACAG	GCTC	ATCTCGCTTACCCTCAAGAACCT
G8N_3F	GTCTCGTGGGCTCGGAGATGTGTATAAGAGACAG	TGAAGCT	ATCTCGCTTACCCTCAAGAACCT
G8N_1R	TCGTCGGCAGCGTCAGATGTGTATAAGAGACAG	G	CTGGTTGATTCCGTTTTCCTG
G8N_2R	TCGTCGGCAGCGTCAGATGTGTATAAGAGACAG	AAC	CTGGTTGATTCCGTTTTCCTG
G8N_3R	TCGTCGGCAGCGTCAGATGTGTATAAGAGACAG	TGGACAAAG	CTGGTTGATTCCGTTTTCCTG

Lipofection

The day before the lipofection, $5x10^5$ cells were plated in 60 mm-diameter plates, in the specific medium for fibroblasts. The cells were then incubated at 37°C to reach a confluence of 70-80%. The next day two test tubes were prepared for each sample to be transfected. In the first tube the plasmid vector pcDNA3 or pcDNA3-EGFP was diluted in 250 µL of Opti-MEM medium and mixed gently; in the second one each selected transfection agent was added to 250 µL of Opti-MEM medium depending on volumes reported in table S4. This last step was carried out using polystyrene tubes, a material capable of preventing the adhesion of the transfecting agent to the surface of the tube itself, to facilitate its dilution with the medium and, subsequently, the formation of complexes with DNA. Subsequently the two solutions were combined and mixed and then incubated at room temperature from 15 to 30 minutes depending on the transfecting agent used, to promote the formation of complexes between cationic lipids and DNA. After removing the spent medium from the cells and replacing it with fresh serum-free medium, it was possible to add the cationic lipid-DNA complexes previously prepared to each well, favouring their mixing through circular and cross movements. In the wells corresponding to the negative controls only the medium and the liposomal agents were added, without the plasmid DNA. Finally, the plates were incubated at 37°C and, after 4 hours, the medium was replaced with complete medium, to limit toxic effects due to the permanence of the transfecting agents in culture. Transfected cells were incubated at 37°C in an 80% humidified atmosphere, containing 5% CO_2.

Table S4. Transfection agents used for lipofection tests. The table summarizes the various transfecting agents used in the fibroblast lipofection tests, their volumes, the amount of DNA used and the relationship between DNA and transfecting agent.

Abbreviation	Transfection agent	Amount of DNA (µg)	Volume of the transfection agent (µL)	Ratio DNA:transfection agent	Note
**L-NT**	**Lipofectamine 2000**	-	20 µL	-	
**L1**	**Lipofectamine 2000**	3 µg	20 µL	-	
**L2**	**Lipofectamine 2000 + Plus Reagent**	3 µg	20 µL	-	Addition of **Plus Reagent** (60 µL)
**T-NT**	**TransIT-X2**	-	7,5 µL	-	
**T1**	**TransIT-X2**	2,5 µg	7,5 µL	1:3	
**T2**	**TransIT-X2**	2,5 µg	10 µL	1:4	
**E-NT**	**Effectene**	-	10 µL	-	
**E1**	**Effectene**	0,4 µg	10 µL	1:25	
**E2**	**Effectene**	0,4 µg	20 µL	1:50	
**J-NT**	**JetPRIME**	-	4 µL	-	
**J1**	**JetPRIME**	2 µg	4 µL	1:2	
**J2**	**JetPRIME**	2 µg	8 µL	1:4	
**X-NT**	**X-TremeGENE**	-	3 µL	-	
**X1**	**X-TremeGENE**	1,00 µg	3 µL	1:3	
**X2**	**X-TremeGENE**	1,00 µg	6 µL	1:6	
**N-NT**	**Nano-Juice**	-	1,25 µL	-	Addition of **Transfection Booster** (1,25 µL)
**N1**	**Nano-Juice**	1,25 µg	1,25 µL	1:1	Addition of **Transfection Booster** (1,25 µL)
**N2**	**Nano-Juice**	1,25 µg	2,5 µL	1:2	Addition of **Transfection Booster** (1,25 µL)
**N3**	**Nano-Juice**	1,25 µg	1,25 µL	1:1	Addition of **Transfection Booster** (2,5 µL)
**N4**	**Nano-Juice**	1,25 µg	2,5 µL	1:2	Addition of **Transfection Booster** (2,5 µL)
**F-NT**	**Fail-Safe**	-	8 µL	-	
**F1**	**Fail-Safe**	2 µg	8 µL	1:4	
**F2**	**Fail-Safe**	4 µg	16 µL	1:4	

Table S5. Barcodes for NGS libraries. For each barcode are reported the nucleotide sequence and orientation.

Name	Sequence	Orientation
N701	CAAGCAGAAGACGGCATACGAGATTCGCCTTAGTCTCGTGGGCTCGG	Forward
N702	CAAGCAGAAGACGGCATACGAGATCTAGTACGGTCTCGTGGGCTCGG	
N703	CAAGCAGAAGACGGCATACGAGATTTCTGCCTGTCTCGTGGGCTCGG	
N704	CAAGCAGAAGACGGCATACGAGATGCTCAGGAGTCTCGTGGGCTCGG	
N705	CAAGCAGAAGACGGCATACGAGATAGGAGTCCGTCTCGTGGGCTCGG	
N706	CAAGCAGAAGACGGCATACGAGATCATGCCTAGTCTCGTGGGCTCGG	
N707	CAAGCAGAAGACGGCATACGAGATGTAGAGAGGTCTCGTGGGCTCGG	
N708	CAAGCAGAAGACGGCATACGAGATCCTCTCTGGTCTCGTGGGCTCGG	
N709	CAAGCAGAAGACGGCATACGAGATAGCGTAGCGTCTCGTGGGCTCGG	
N710	CAAGCAGAAGACGGCATACGAGATCAGCCTCGGTCTCGTGGGCTCGG	
S501	AATGATACGGCGACCACCGAGATCTACACTAGATCGCTCGTCGGCAGCGTC	Reverse
S503	AATGATACGGCGACCACCGAGATCTACACTATCCTCTTCGTCGGCAGCGTC	
S505	AATGATACGGCGACCACCGAGATCTACACGTAAGGAGTCGTCGGCAGCGTC	
S506	AATGATACGGCGACCACCGAGATCTACACACTGCATATCGTCGGCAGCGTC	
S507	AATGATACGGCGACCACCGAGATCTACACAAGGAGTATCGTCGGCAGCGTC	
S508	AATGATACGGCGACCACCGAGATCTACACCTAAGCCTTCGTCGGCAGCGTC	
S5010	AATGATACGGCGACCACCGAGATCTACACCGTCTAATTCGTCGGCAGCGTC	
S5011	AATGATACGGCGACCACCGAGATCTACACTCTCTCCGTCGTCGGCAGCGTC	
S5013	AATGATACGGCGACCACCGAGATCTACACTCGACTAGTCGTCGGCAGCGTC	
