# Supplementary material for: Suppressing gain-of-function proteins via CRISPR/Cas9 system in SCA1 cells
Source: Sci Rep. 2022 Nov 24;12:20285. doi: 10.1038/s41598-022-24299-y (PMC9700751; doi:10.1038/s41598-022-24299-y)
Supplement: Supplementary file 15 — Supplementary Information 15. [file 41598_2022_24299_MOESM15_ESM.pdf]

Figure 2a

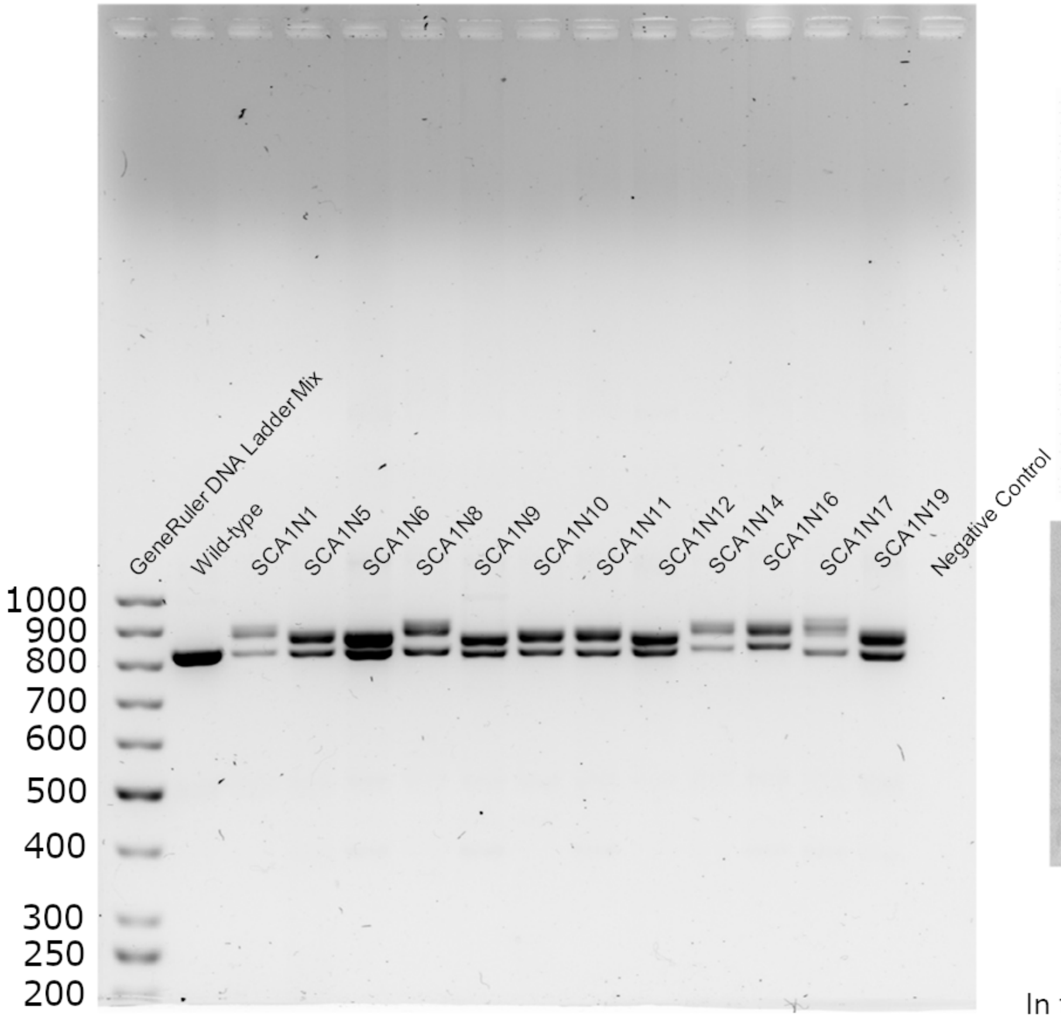

Figure 2b

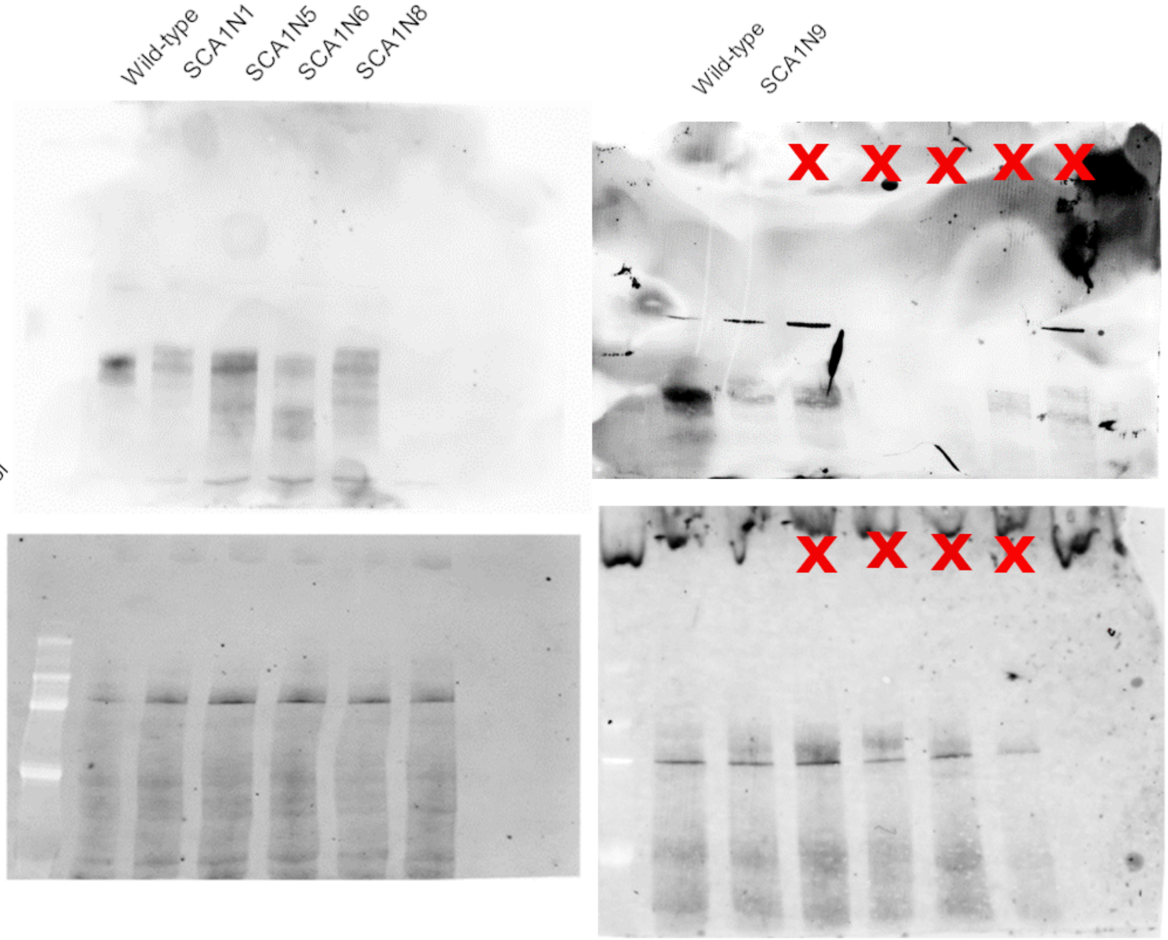

In the lanes indicated with X, proteins extracted from other SCA1 fibroblasts, not reported in the manuscript, were loaded.

Figura 2b

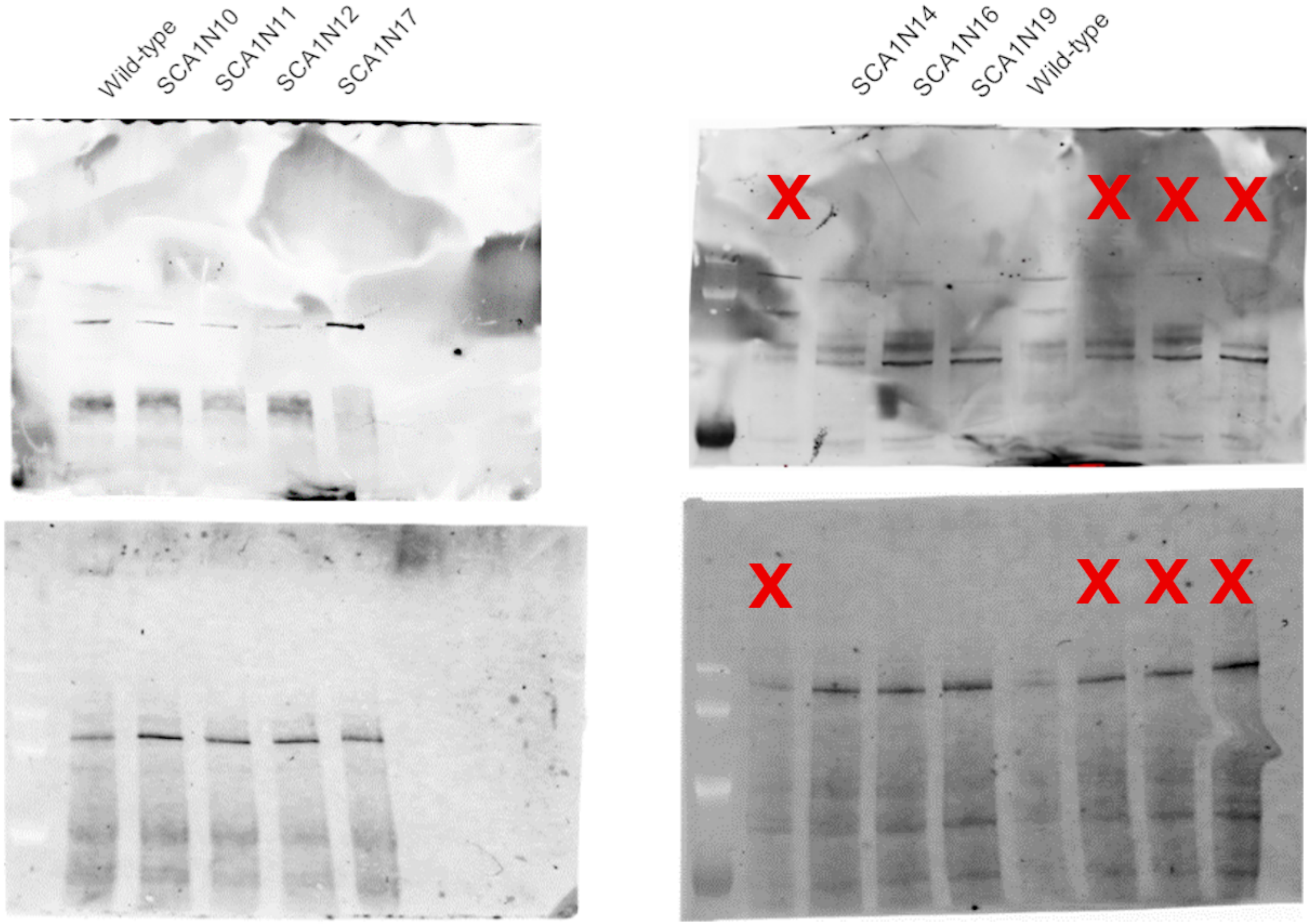

In the lanes indicated with X, proteins extracted from other SCA1 fibroblasts, not reported in the manuscript, were loaded.

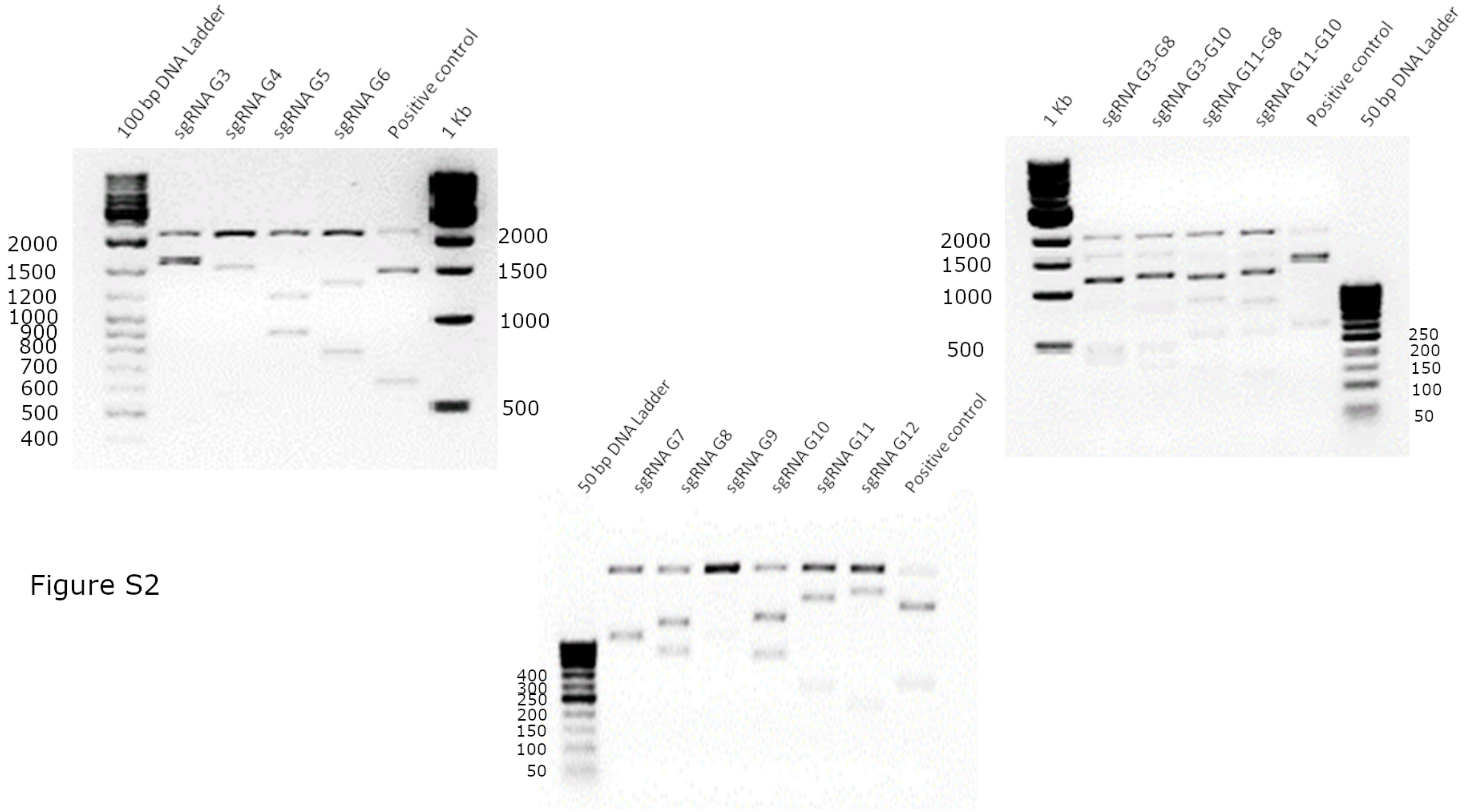
100 bp DNA Ladder
sgRNA G3
sgRNA G4
sgRNA G5
sgRNA G6
Positive control
1 Kb
2000
1500
1200
1000
900
800
700
600
500
400
2000
1500
1000
500
1 Kb
sgRNA G3-G8
sgRNA G3-G10
sgRNA G11-G8
sgRNA G11-G10
Positive control
50 bp DNA Ladder
2000
1500
1000
500
250
200
150
100
50
50 bp DNA Ladder
sgRNA G7
sgRNA G8
sgRNA G9
sgRNA G10
sgRNA G11
sgRNA G12
Positive control
400
300
250
200
150
100
50

Figure S2

Figure S3

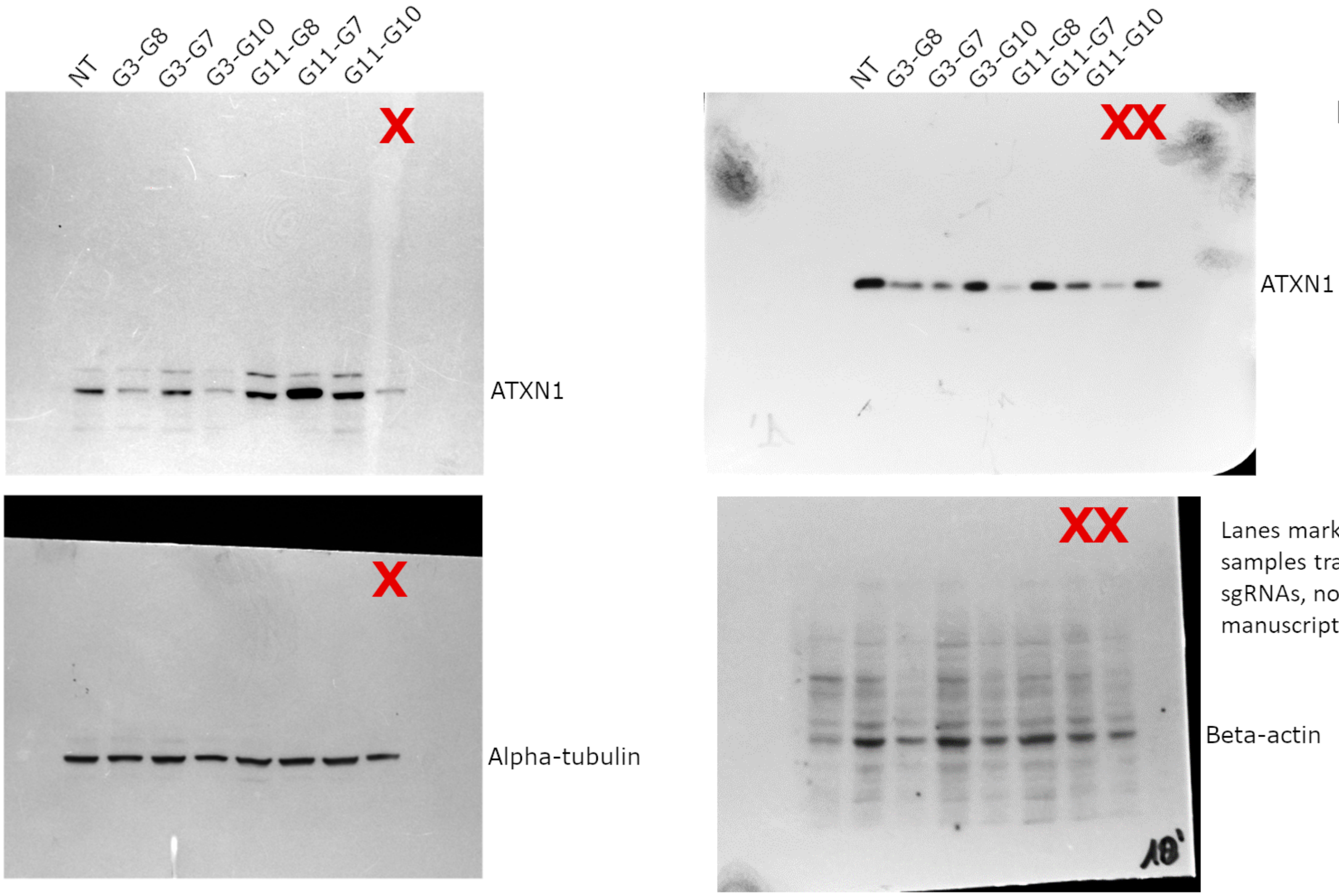

Lanes marked with X correspond to samples transfected with other sgRNAs, not included in the manuscript.

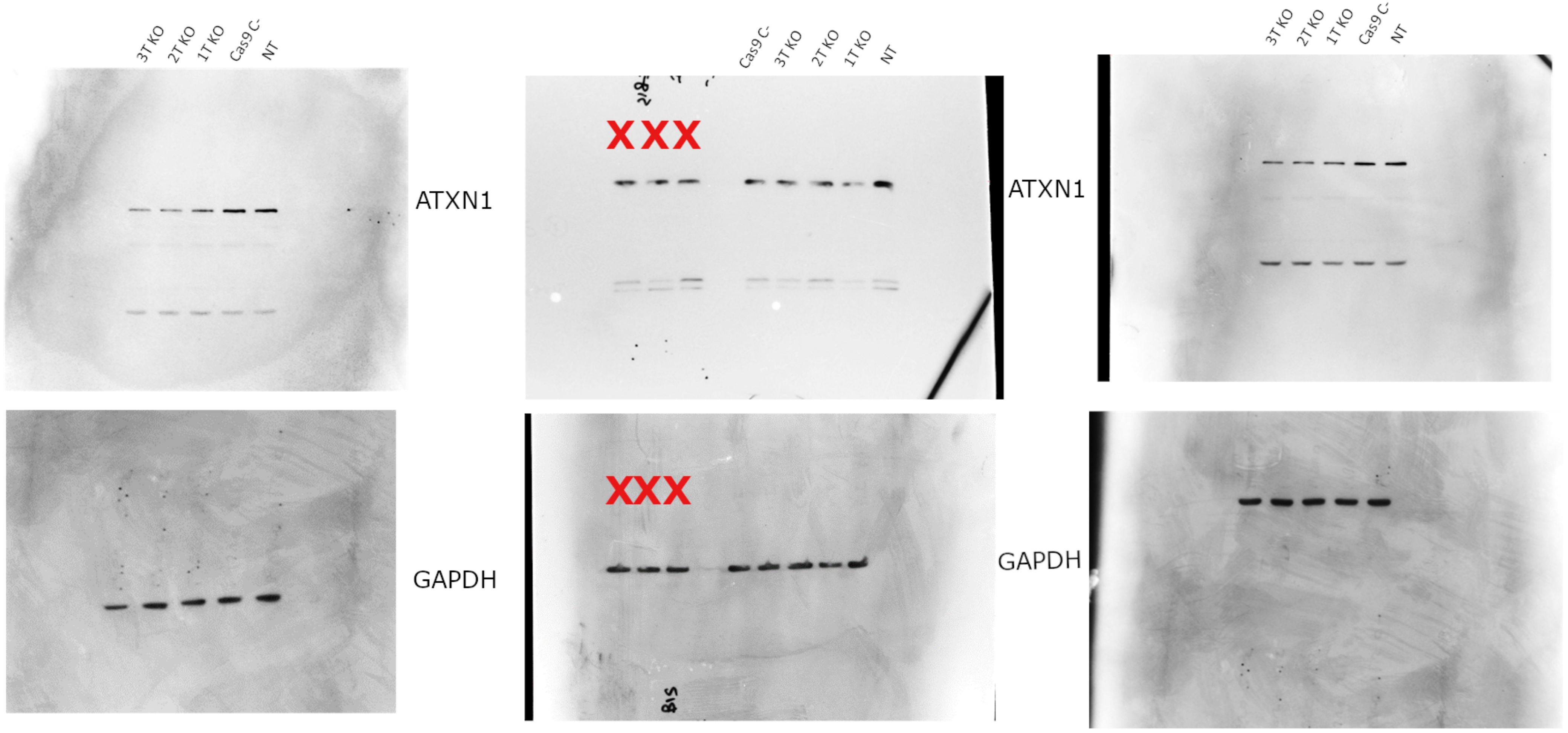

Figure S4

Lanes indicated with X correspond to samples transfected with a gene editing approach designed to replace part of the ATXN1 gene.

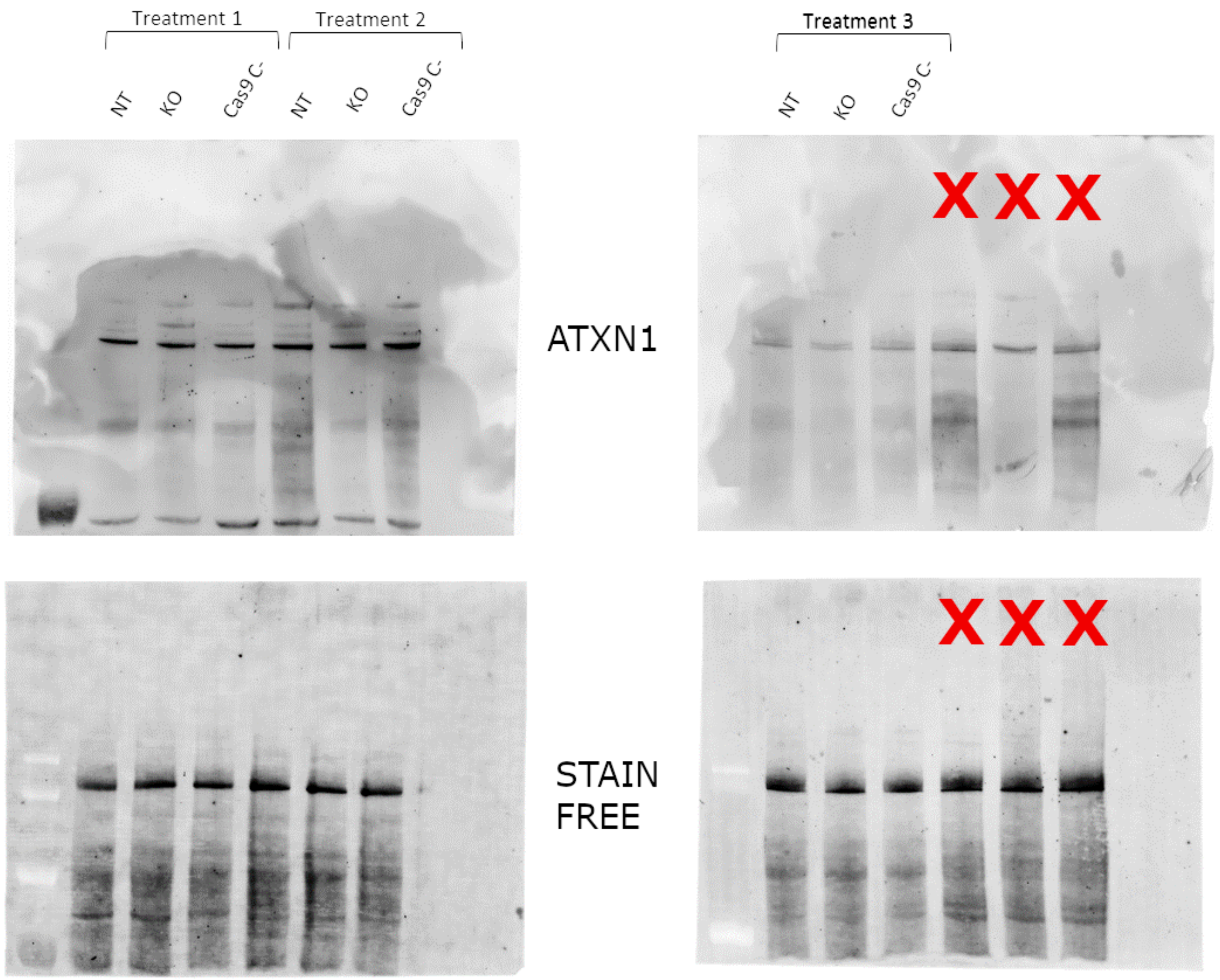

Figure S5a

Lanes marked with X correspond to samples transfected with other sgRNAs, not included in the manuscript.

SCA1N5

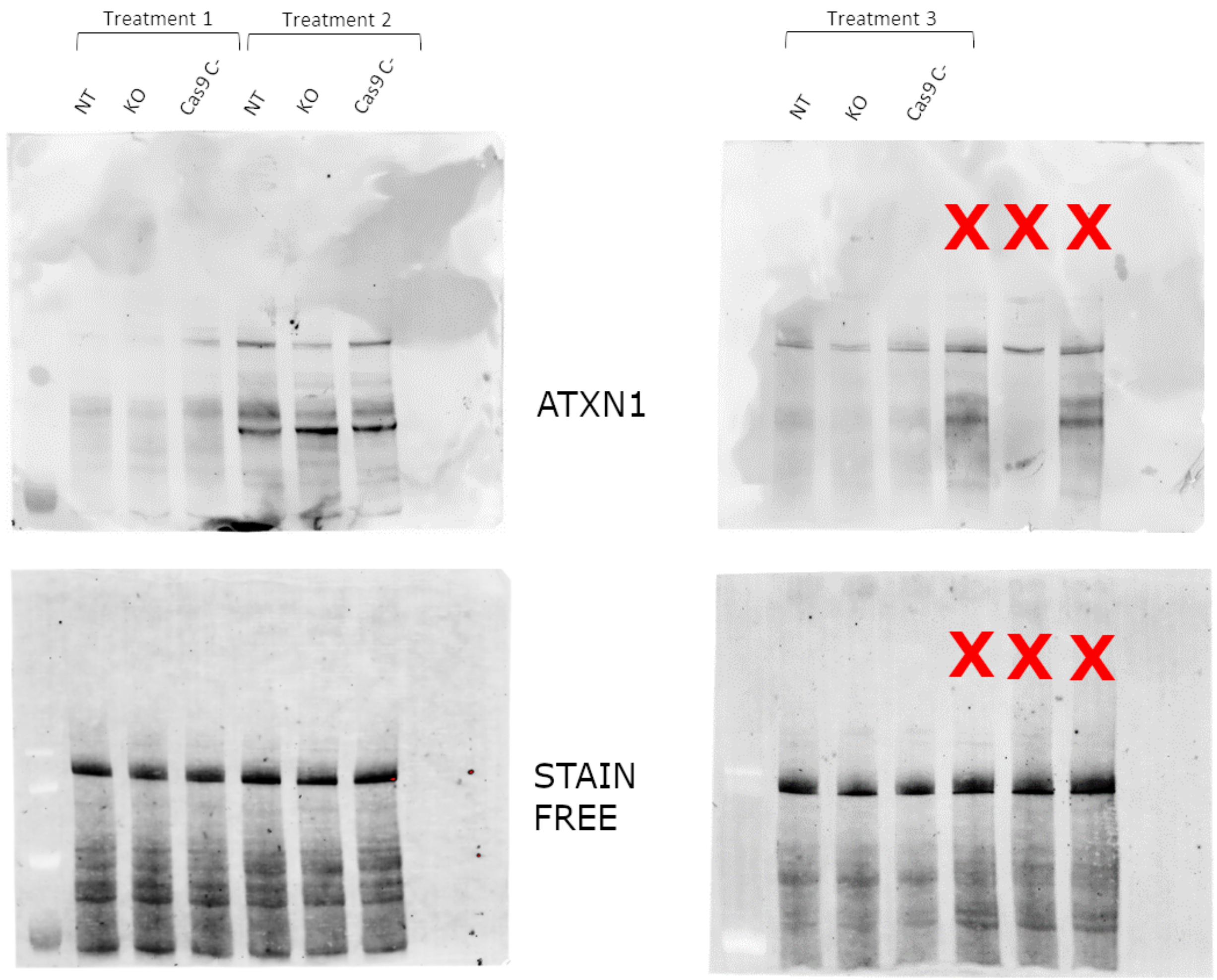

Figura S5c

Lanes marked with X correspond to samples transfected with other sgRNAs, not included in the manuscript.

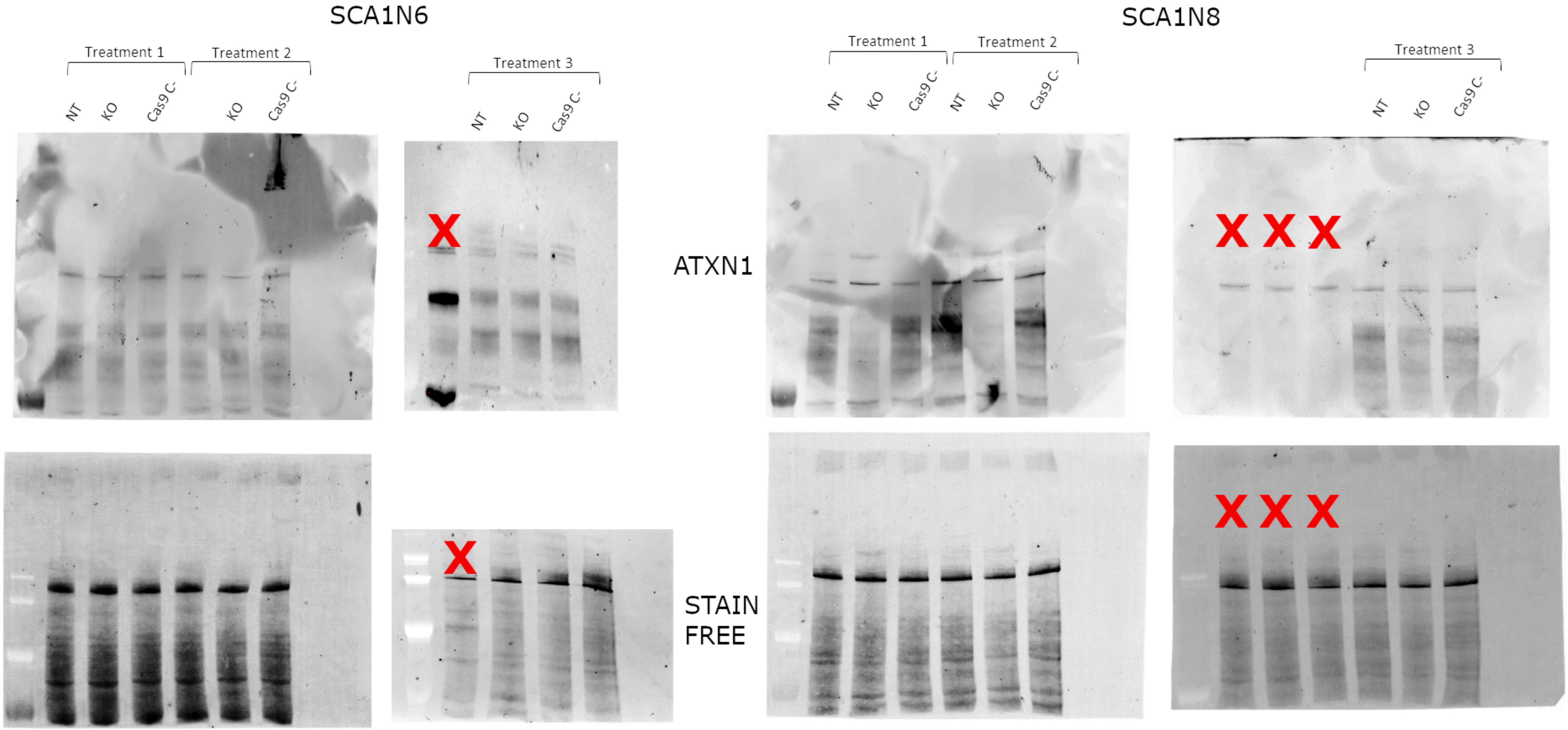

A protein extract of healthy fibroblasts was loaded into the lane indicated with the X above.

Figure S5e-g

Lanes marked with X correspond to samples transfected with other sgRNAs, not included in the manuscript.

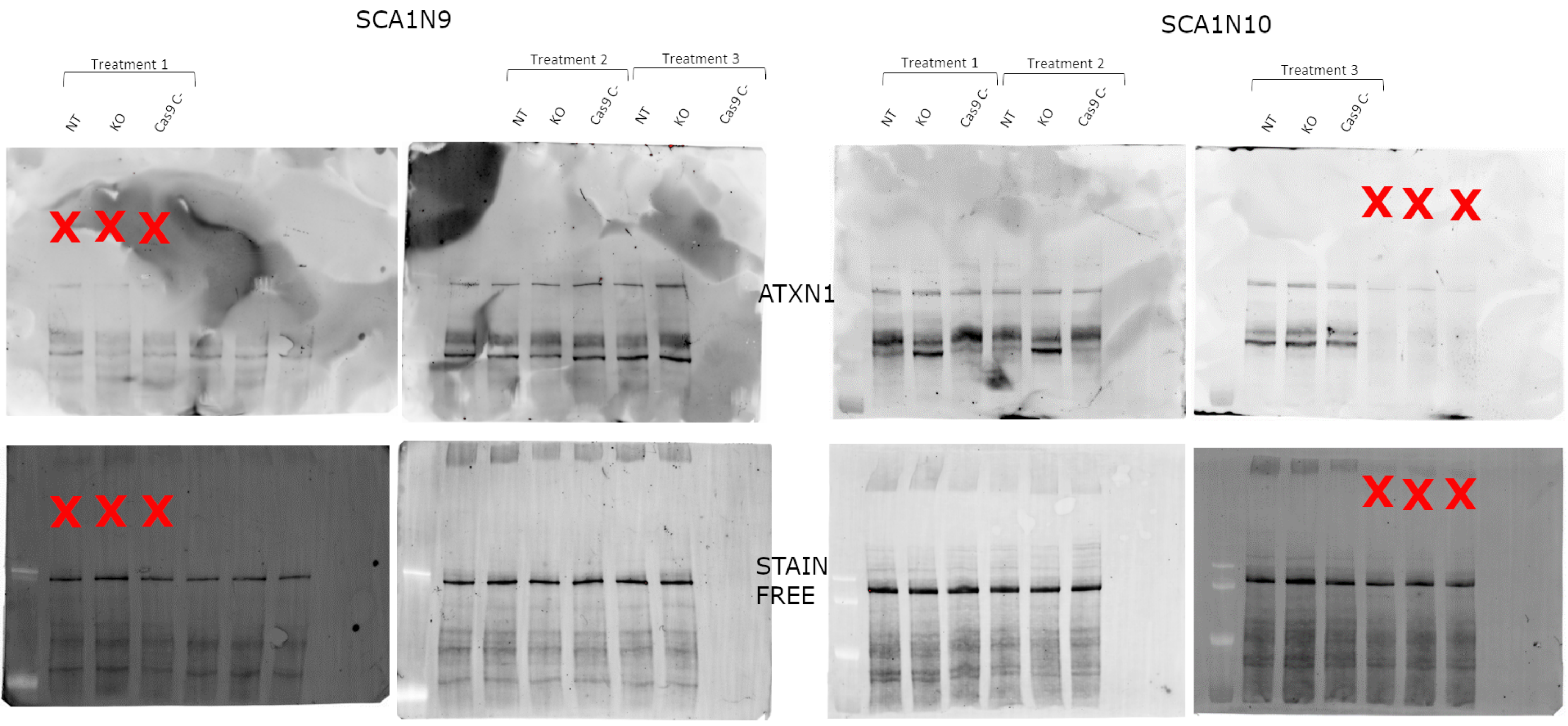

Figure S5i-m

Lanes marked with X correspond to samples transfected with other sgRNAs, not included in the manuscript.

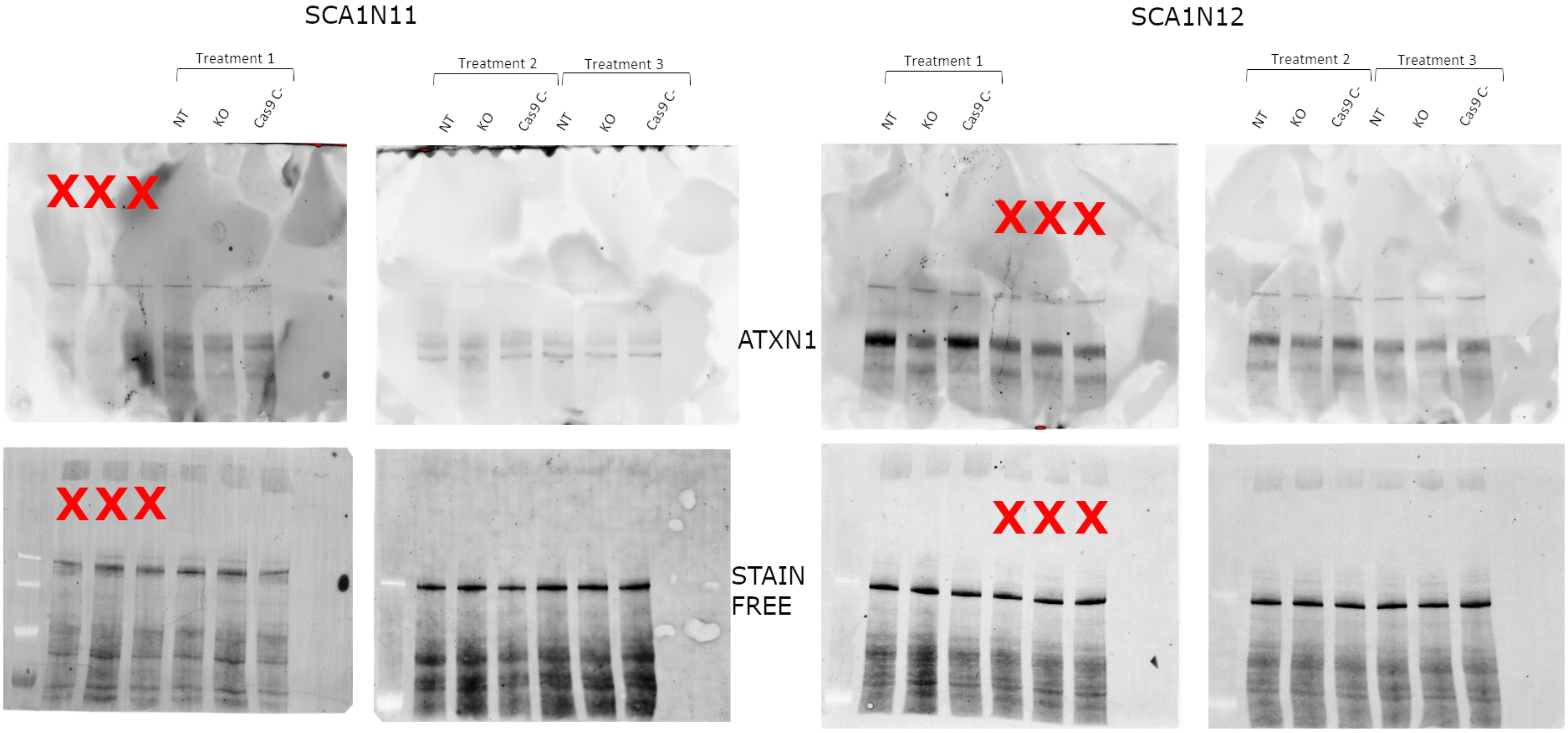

Lanes marked with X correspond to samples transfected with other sgRNAs, not included in the manuscript.

Figure S5o-q

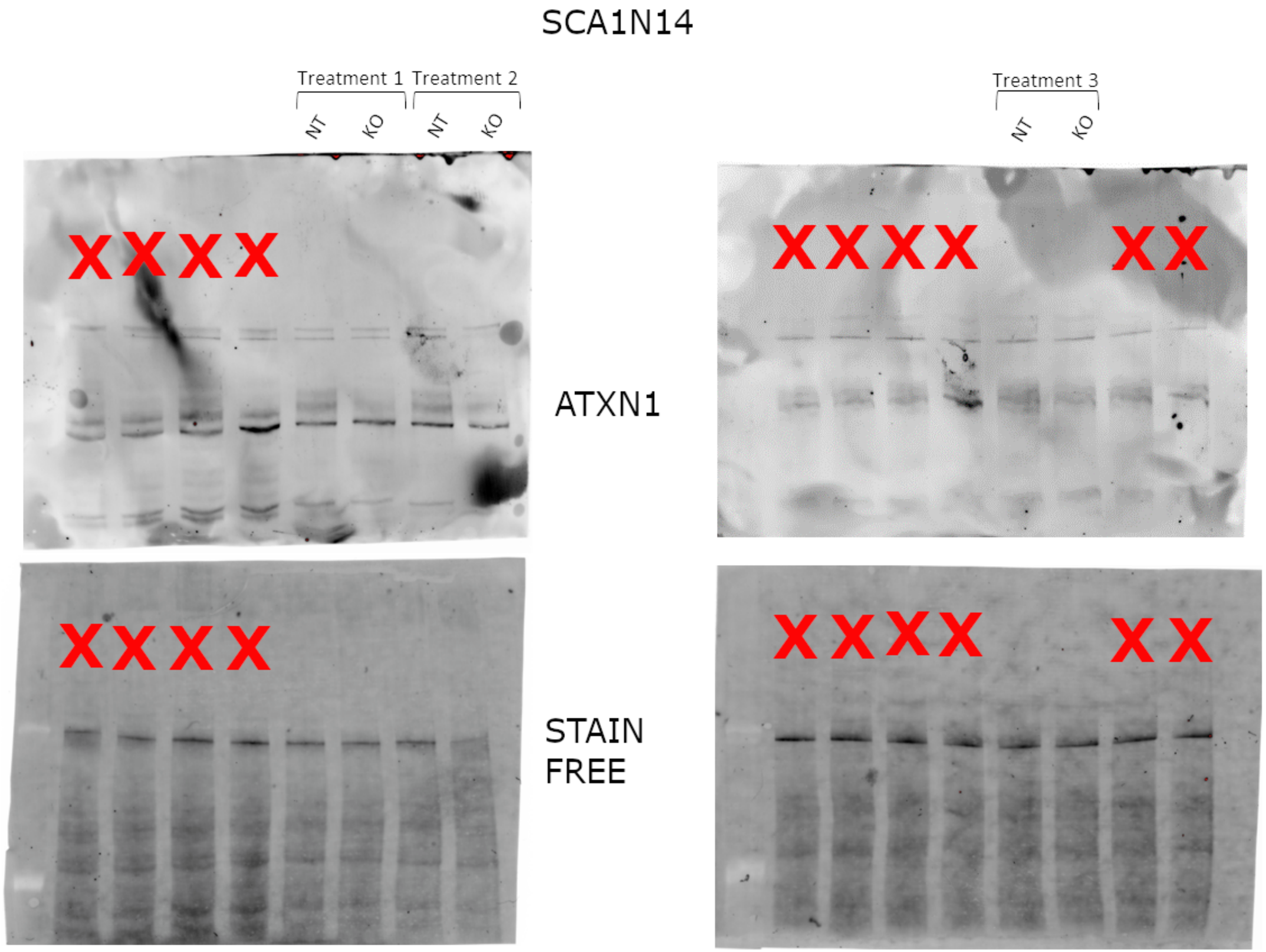

Figure S5s

Lanes indicated with X correspond to samples transfected with a gene editing approach designed to replace part of the ATXN1 gene or with other sgRNAs not reported in the manuscript.

SCA1N17

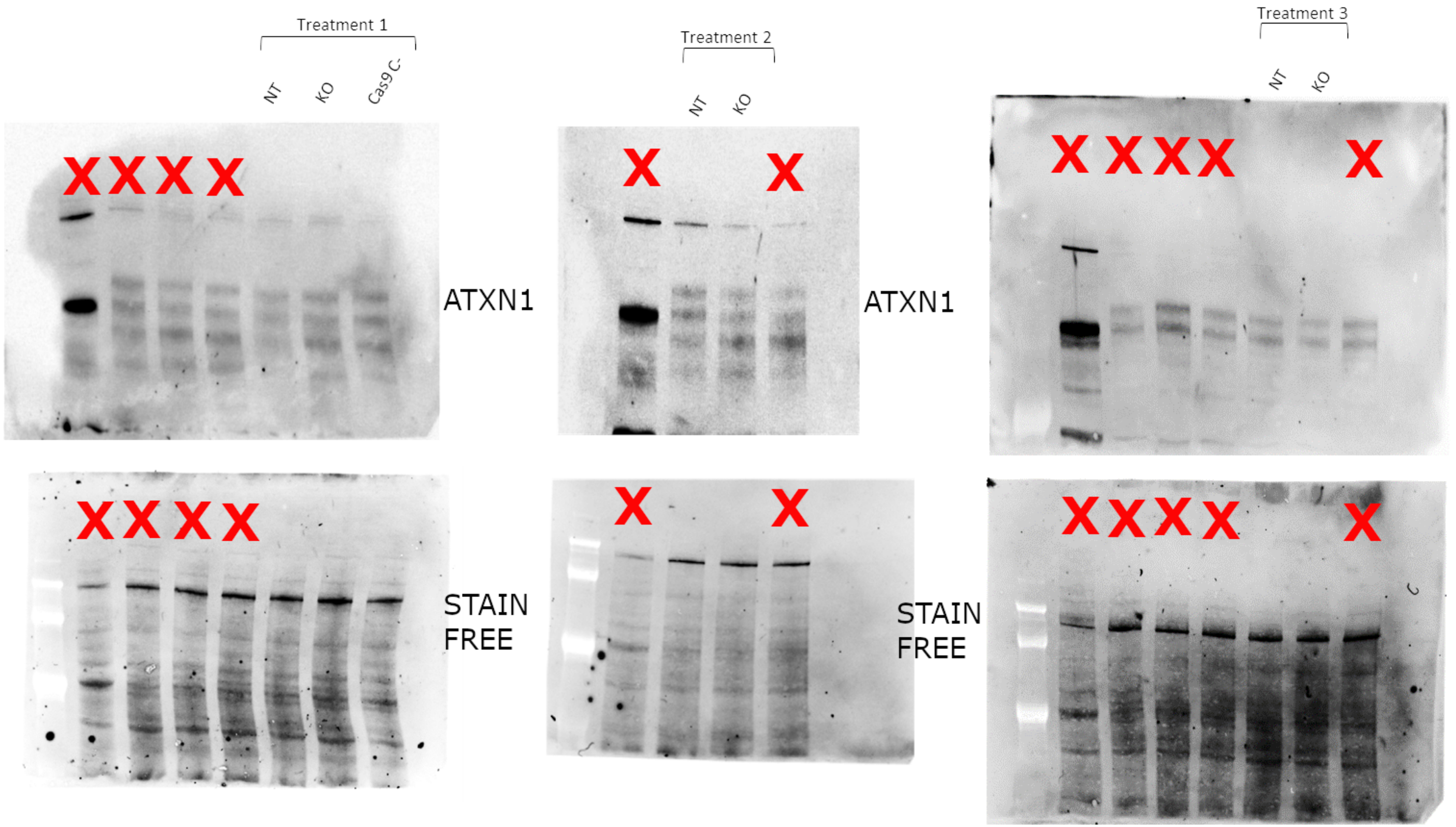

Figure S5u

Lanes indicated with X correspond to samples transfected with a gene editing approach designed to replace part of the ATXN1 gene or with other sgRNAs not reported in the manuscript.

SCA1N14

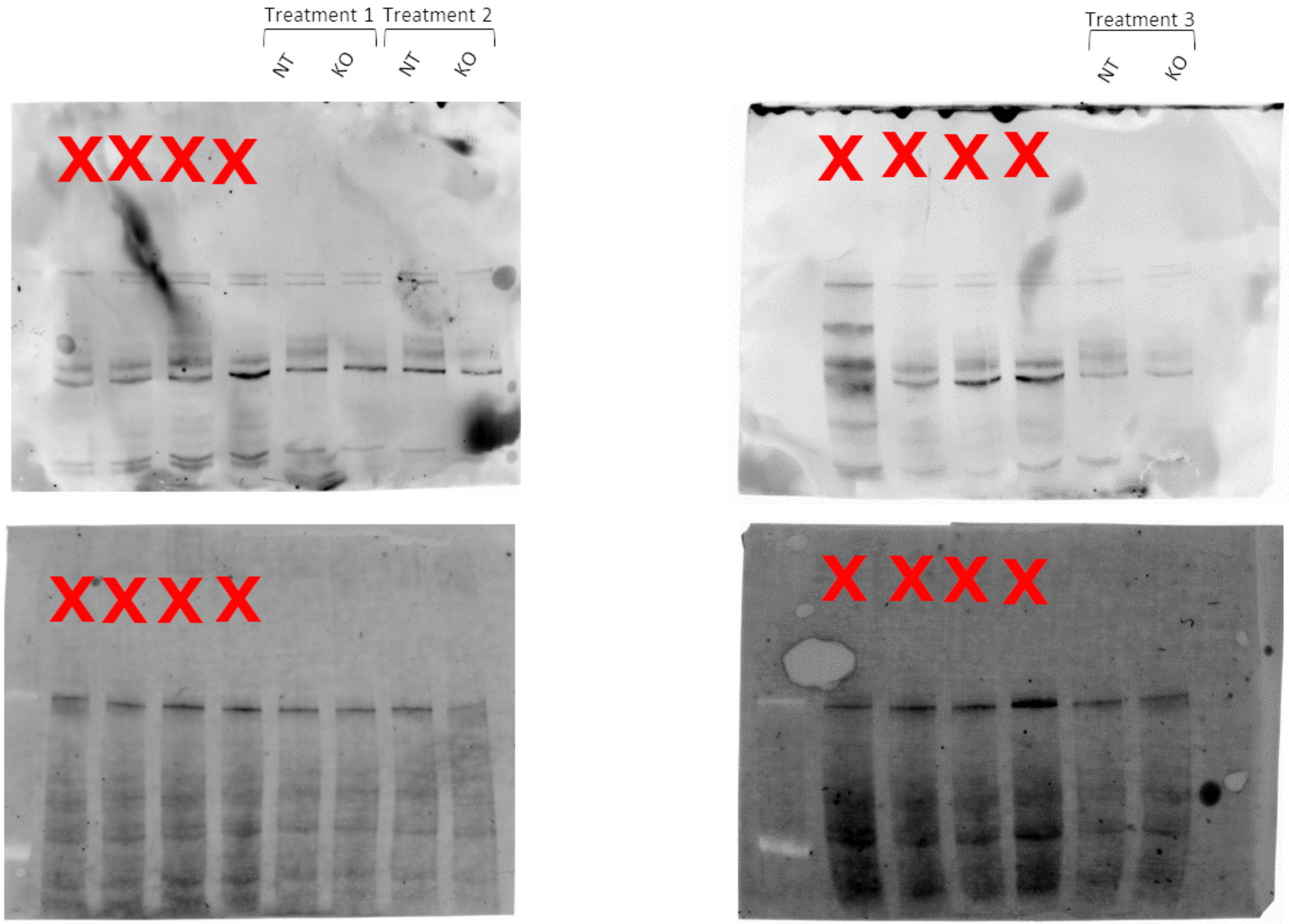

Figure S6a

Lanes marked with X correspond to samples transfected with other sgRNAs, not included in the manuscript.

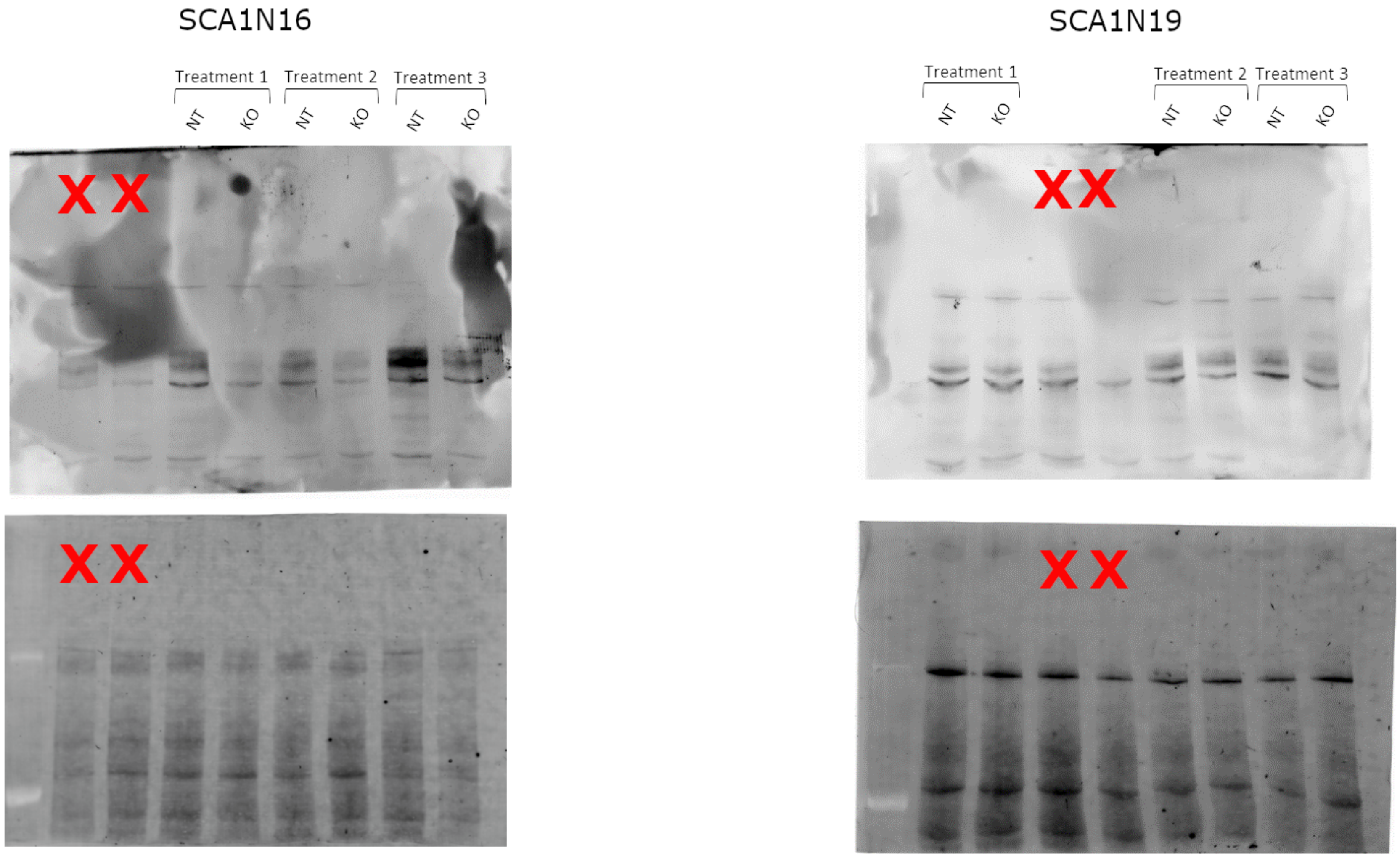

Figura S6c-e

Lanes marked with X correspond to samples transfected with other sgRNAs, not included in the manuscript.

Figure S8

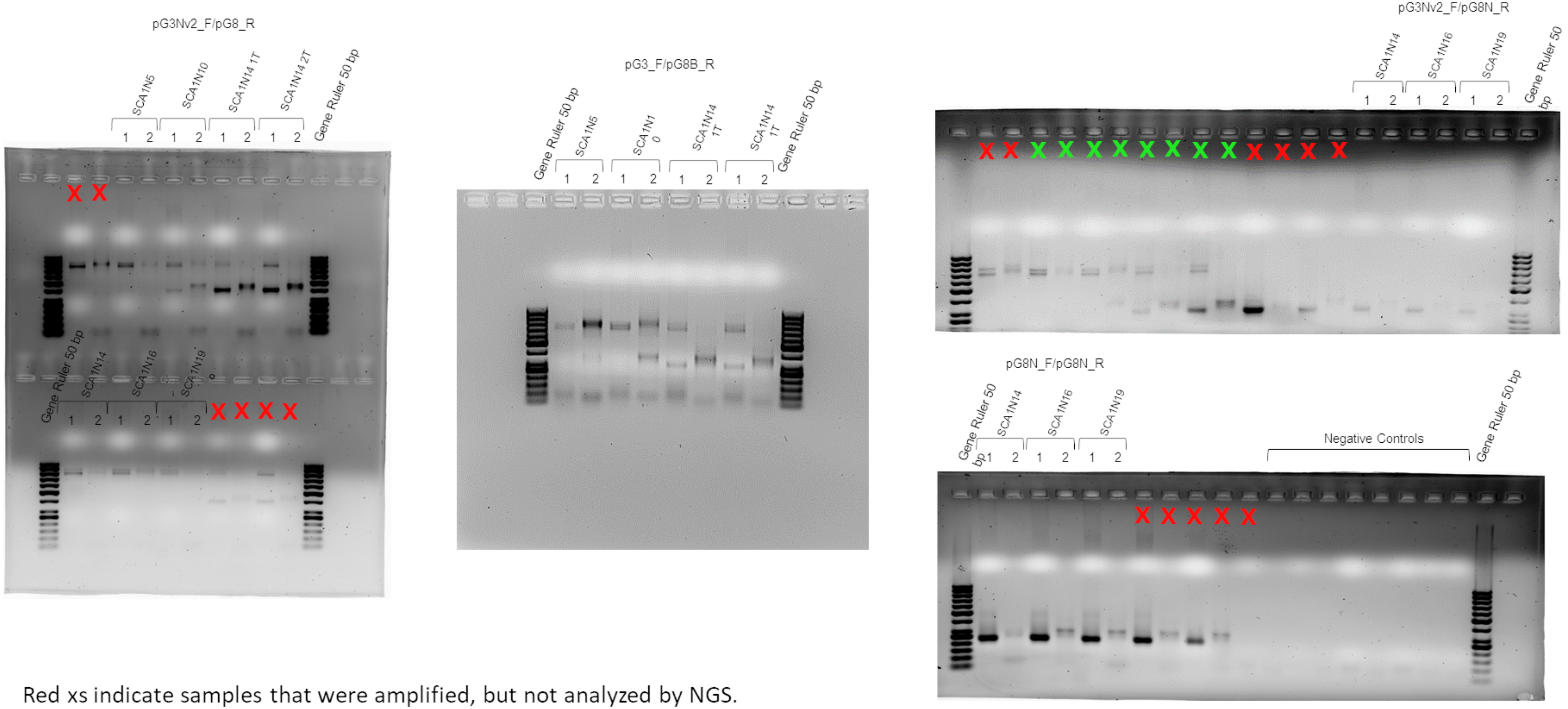

Red xs indicate samples that were amplified, but not analyzed by NGS.
Green xs indicate amplicons remade due to problems during PCRs.
